# External Catalyst- and Additive-Free Photo-Oxidation of Aromatic Alcohols to Carboxylic Acids or Ketones Using Air/O_2_

**DOI:** 10.3390/molecules28073031

**Published:** 2023-03-28

**Authors:** Meng Xu, Jinhua Ou, Kejun Luo, Rongtao Liang, Jian Liu, Ni Li, Bonian Hu, Kaijian Liu

**Affiliations:** 1 School of Materials Science and Engineering, Hunan Institute of Technology, Hengyang 421002, China; 2 Analytical Testing Center, Changsha Research Institute of Mining and Metallurgy Co., Ltd., Changsha 410012, China

**Keywords:** aromatic alcohols, air/O_2_, additive-free, catalyst-free, photo-oxidation

## Abstract

We present an environment-friendly and highly efficient method for the oxidation of aromatic alcohols to carboxylic acids or ketones in air via light irradiation under external catalyst-, additive-, and base-free conditions. The photoreaction system exhibits a wide substrate scope and the potential for large-scale applications. Most of the desired products are easily obtained via recrystallization and separation from low-boiling reaction medium acetone in good yields, and the products can be subsequent directly transformed without further purification.

## 1. Introduction

The selective oxygenation of aromatic alcohols to acids, which are significant raw materials and intermediates for the production of food preservatives, dyes, plasticizers, and sugars, is a fundamental reaction in organic synthesis [1,2,3,4,5,6]. For example, 1, 4-terephthalic acid is an important monomer in the synthesis of polyethylene terephthalate (PET, commonly known as polyester resin), whose annual global consumption amounts to about 13 million tons. Currently, various oxidizing agents, such as chromium [7] and manganese [8,9] compounds, hypervalent iodine reagents [10,11], and activated dimethyl sulfoxide (DMSO) [12], have been extensively used for this transformation. However, problems, such as toxicity, high cost, and metal waste formed by these oxidants, have limited their potential in practical applications. In comparison to other agents, O_2_ has received substantial attention because of its advantages of low cost, high atom efficiency, and minimal byproducts [13,14,15,16,17,18,19,20,21,22]. The selective oxygenation of alcohols to acids using O_2_ or air as the oxidant remains a significant challenge, possibly because aerobic oxidation of alcohols stops at the aldehyde stage and only a small fraction of the aldehydes are converted to carboxylic acids [23,24]. The oxygenation of alcohols to acids with O_2_ has been conducted in thermal reaction systems with external additives, such as strong bases [25], nonmetallic oxidants [26], or the adoption of transition metal catalysts [14,15,16,17,18,19,20,27,28]. In 2018, we developed a bis(methoxypropyl) ether-promoted oxidation system without an external initiator, catalyst, or base [29]. The protocol is eco-friendly and practical. However, it requires costly ether and high temperatures.

**Scheme 1:**
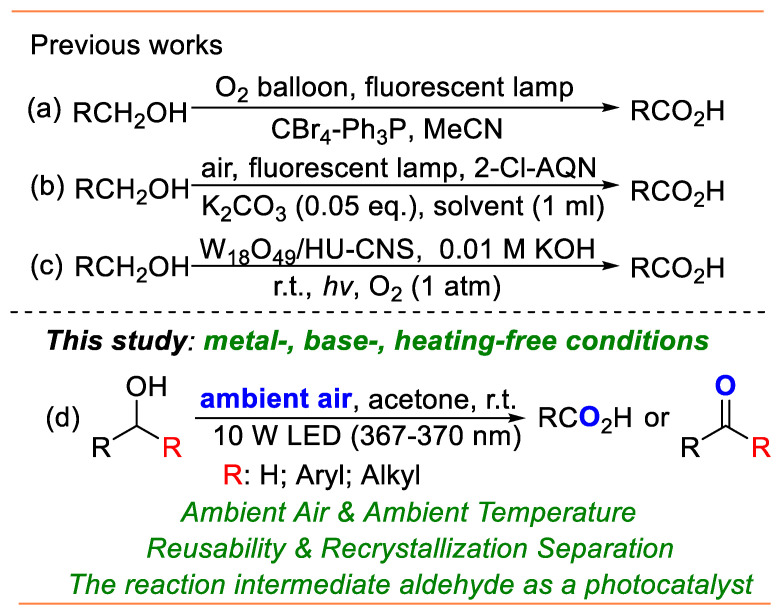
Oxidation of alcohols via photo-oxidation of alcohols (**a**) CBr_4_–Ph_3_P catalyzed oxidation of alcohols to the acids, (**b**) 2-chloroanthraquinone catalyzed oxidation of alcohols to the acids, (**c**) W_18_O_49_/g-C_3_N_4_ promoted the transformation of benzyl alcohol to target acids, (**d**) the oxygenation of aromatic alcohols to the target acids or ketones without external additives.

Compared to the thermal system, the photo-oxidation of aromatic alcohols to acids with air/O_2_ as an oxidant has attracted significant attention from those interested in the sustainable and environmentally friendly syntheses of chemicals [30,31,32,33,34,35] because O_2_ can be activated to form reactive oxygen species using photocatalysts [36,37]. To date, several metal-based [30] and inorganic semiconductor [32,33,34,35] photocatalysts and small molecular- or macromolecular-based organic semiconductor [31,38,39] photocatalysts have been explored for the oxygenation of alcohols to acids with O_2_ as the oxidant. For instance, Sugai et al. [33] reported a CBr_4_–Ph_3_P catalyzed system for the oxygenation of alcohols to the corresponding acids with a fluorescent lamp using O_2_ as the oxidant (Scheme 1a). In 2013, Itoh et al. [31] revealed a method for the 2-chloroanthraquinone (2-Cl-AQN)-catalyzed photo-oxidation of alcohols to obtain carboxylic acids in air under visible light irradiation (Scheme 1b). Recently, Xiao et al. [34] prepared W_18_O_49_/g-C_3_N_4_ photocatalysts to promote the transformation of benzyl alcohol to target acids with O_2_ (Scheme 1c). Although the systems described above exhibited several excellent characteristics, all of them require an external catalytic photosensitizer, which is obtained through either complex synthesis or expensive commercial purchase. Toxic additives, high costs, and isolation difficulties render these methods unfeasible for practical application or mass production.

Recently, several simple and eco-friendly methods for synthesizing aromatic acids by photoinduced oxidation of aldehydes were developed at room temperature [40,41]. However, there is no literature on preparation methods of aromatic acid from aromatic alcohols using O_2_ as the sole oxidant under external catalyst-, additive-, and base-free conditions. As the continuation of our interest in selective oxidation utilizing O_2_ as the oxidant and environmentally friendly synthesis protocol [29,42,43,44,45,46,47,48], we report an efficient and practical photocatalytic system for the oxygenation of aromatic alcohols to the target acids or ketones without external additives (Scheme 1d). Compared with previously reported systems, the protocol was performed successfully with excellent yields at mild reaction conditions, which exhibit a simple post-treatment and could also be applied on one-pot sequential transformation.

## 2. Results and Discussion

First, benzyl alcohol (**1a**) was used as a test substrate with O_2_ as the oxidant, and a 63% yield of the desired benzoic acid (**3a**) was placed under LED irradiation (390–395 nm, 10 W) for 24 h in MeCN (Table 1, entry 1). Based on the GC–MS results, the reaction medium had a significant impact on oxidation efficiency (entries 1–7), and the oxidation reaction produced a 74% yield of benzoic acid (**3a**) with acetone. As the light source has a significant influence on the oxygenation of aromatic alcohols to the corresponding acids, we investigated the effect of varying the irradiation wavelength of the light source (entries 8–15). The oxidation reaction afforded a 98% yield of (**3a**) at an irradiation wavelength of 367–370 nm over 24 h. Notably, there was no difference in yield when O_2_ was replaced with air (entry 16). However, the target product was not obtained when O_2_ was substituted with N_2_ (entry 17). In addition, the reaction was ineffective in the absence of illumination (entry 18).

The photocatalytic system is also highly efficient for the selective oxidation of a range of aromatic alcohols to produce the desired acids or ketones under the above-mentioned conditions (Table 2). In particular, benzyl alcohols substituted with electron-withdrawing or electron-donating groups (**3a**–**3p**) could be oxidized to acids in high yields, except for the strong electron-withdrawing cyano group (**3j**), which was obtained in a moderate yield. (This may be due to the strong electron-withdrawing effect of the cyano group, which reduces the electron cloud density of the benzene ring and benzyl radical activity, resulting in a decreased yield.) The oxidation reaction was also unaffected with the ortho-Cl (or -Me) group, and synthetically useful orthosubstituted compounds (**3l** and **3m**) were tolerated. Notably, neither the metasubstituted compounds (**3n** and **3o**), nor the polysubstituted compounds (**3p**) had a significant influence on the conversion, and all generated the target products in good yields. In addition, the oxidation of the bireactive functional substrate 1,4-phenylenedimethanol proceeded smoothly with O_2_ and provided the target acid in a 71% yield (**3q**). Significantly, various heteroaromatic and fused-aromatic alcohols also reacted smoothly to produce the expected products (**3r**–**3u**) in moderate to excellent yields. In particular, 1,2-phenylenedimethanol was also suitable for the system and offered the target product isobenzofuran-1(3*H*)-one (**3v**) in a moderate yield. Regrettably, the oxidation of fatty alcohol produced only a trace amount of the target products (**3w** and **3x**), which may be due to the fact that the stability of primary carbon radicals produced with aliphatic alcohols is less than that of benzyl radicals from aromatic alcohols. In addition, the scope of the aromatic secondary alcohol oxidation reaction was studied under the aforementioned standard conditions. A wide range of 1-phenethylalcohols (**4a**–**4e**) and benzhydrols (**4f**–**4j**) with electron-rich or electron-poor group-substituted aromatic rings performed adequately with O_2_ and smoothly formed the target ketones in excellent yields. β-Substituted 1-phenethylalcohols (**4k**–**4m**) with Cl, Br, or CH_2_Cl groups also reacted efficiently to provide the corresponding acid in 83–90% yields. Furthermore, mandelonitrile can also be efficiently converted to benzoyl cyanide (**4n**), an intermediate of the herbicide metamitron. More importantly, the oxidation of cyclic secondary alcohols was effective, and the target products (**4o**–**4q**) were formed in good to excellent yields. The results presented above demonstrate the generality of the photocatalytic reaction with O_2_ as a reagent for the oxidation of various alcohols to the target acid.

**Scheme 2:**
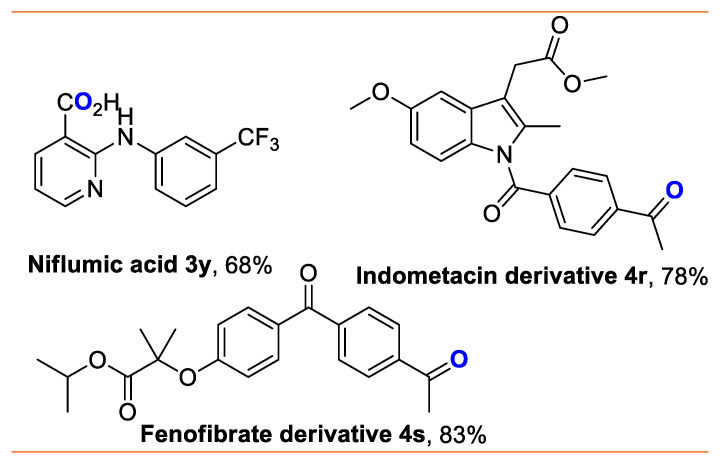
Gram-stage modification of bioactive alcohol.

Pharmaceutical companies show a significant interest in the late-stage structural modification of bioactive natural products. Thus, three bioactive alcohol compounds were synthesized using the proposed photocatalytic oxidation method (Scheme 2). The introduction of a carboxylic acid group to niflumic acid (**3y**), a nonsteroidal anti-inflammatory drug, was easily achieved through this reaction. The oxidation of bioactive secondary alcohol also proceeded smoothly with O_2_ under light irradiation and afforded the target natural product derivatives in good yields, including the antiphlogistic drug indometacin **4r** and antilipemic agent fenofibrate **4s**.

**Scheme 3:**
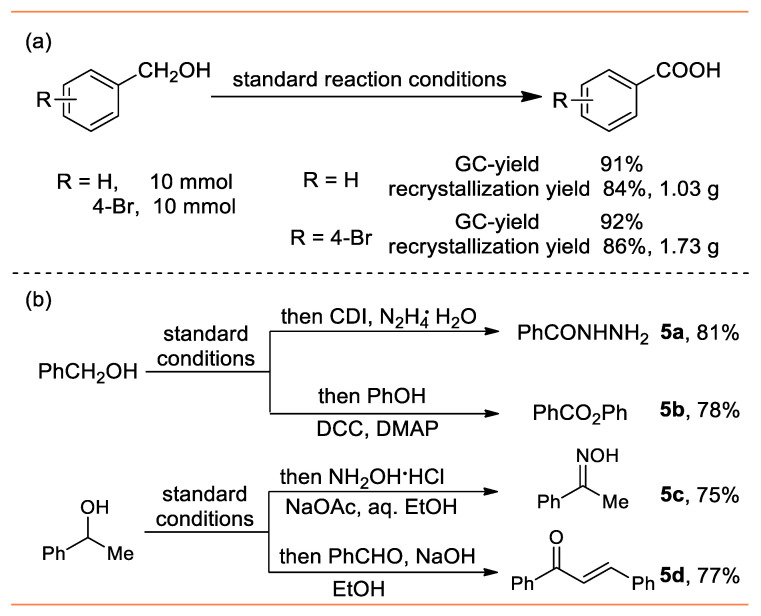
Large-scale and one-pot synthesis of different derivatives (**a**) Gram-stage synthesis of **3a** and **3i**, (**b**) one-pot synthesis of Benzoic acid and acetophenone.

After successfully exploring the oxygenation of different aromatic alcohols, we decided to conduct large-scale oxidation experiments with 10 mmol of aromatic alcohols under the optimal standard conditions (Scheme 3a) to understand the potential synthetic value and practicability of the facile synthesis protocol. Remarkably, the target acid was obtained with the desired GC yield, which was comparable to that of the small-scale reaction. Notably, 84% and 86% yields of benzoic acid (**3a**) (1.03 g) and 4-bromobenzoic acid (**3i**) (1.73 g) were achieved, respectively, via simple recrystallization and separation. Catalyst-free photocatalytic systems are generally more popular from a green energy and industrial point of view and can satisfy demand without purification to remove byproducts and catalyst residues and achieve subsequent direct synthesis under light-driven conditions. To further demonstrate the practicality of the photocatalytic oxidation, four one-pot sequential organic syntheses starting from benzyl alcohol or 1-phenethylalcohols were performed (Scheme 3b). The crude alcohol underwent smooth hydrazidation (**1a → 5a**) [49], esterification (**1a → 5b**) [50], oximation (**1a → 5c**) [51], and Claisen–Schmidt condensation (**1a → 5d**) [52] in the desired yields.

**Figure 1:**
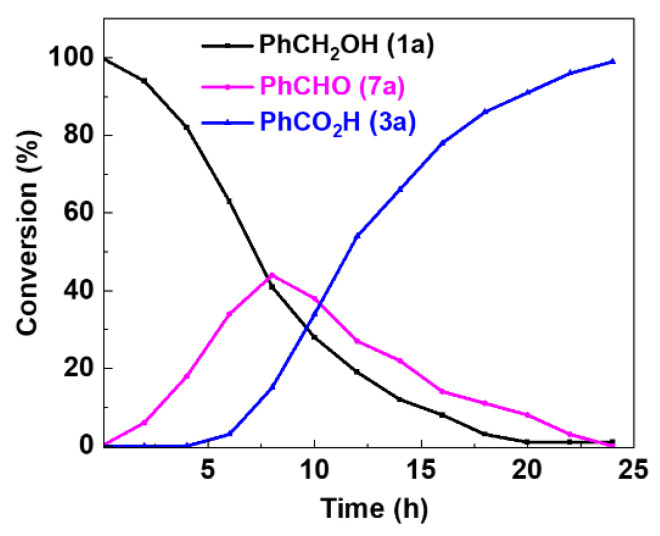
Time course of the oxidation of **1a** to **3a**.

The conversion of phenylmethanol under light irradiation over time was investigated under standard conditions (Figure 1). Phenylmethanol was converted gradually over 24 h, and only a 3% GC yield of benzoic acid was generated within the initial 6 h oxidation stage, after which phenylmethanol was added rapidly until consumed. A yield of 44% (by GC) benzaldehyde was obtained in the first 8 h, before being exhausted at a later stage of the oxidation reaction. The variation can possibly be ascribed to the fact that the alcohol was first transformed to aldehyde, and the obtained aldehyde was then oxidized to acid. In addition, the conversion rate of benzyl alcohol (**1a**) is slow in the early stages and then gradually increases with the formation of benzaldehyde (Figure 1). This phenomenon may be ascribed to the fact that the intermediate benzaldehyde, a carbonyl compound, can act as a triplet state photosensitizer abstracting H from the substrate to form free radicals and promote the conversion of benzyl alcohol [53].

**Figure 2:**
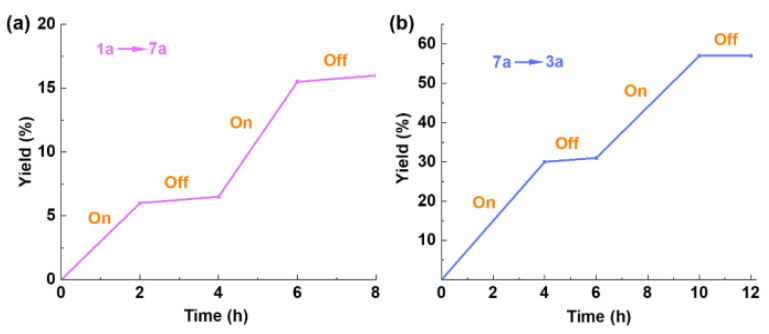
Light irradiation on–off control experiments (**a**) the oxidation of benzyl alcohol to benzaldehyde, (**b**) the oxidation of benzaldehyde to benzyl acid.

To demonstrate the effect of light irradiation on the present method, on–off control experiments were conducted for the oxidation from benzyl alcohols to benzaldehyde and benzaldehyde to benzoic acid. As shown in Figure 2, the oxidation reaction was seriously hindered by the lack of light irradiation, indicating the light dependency of the transformations.

**Scheme 4:**
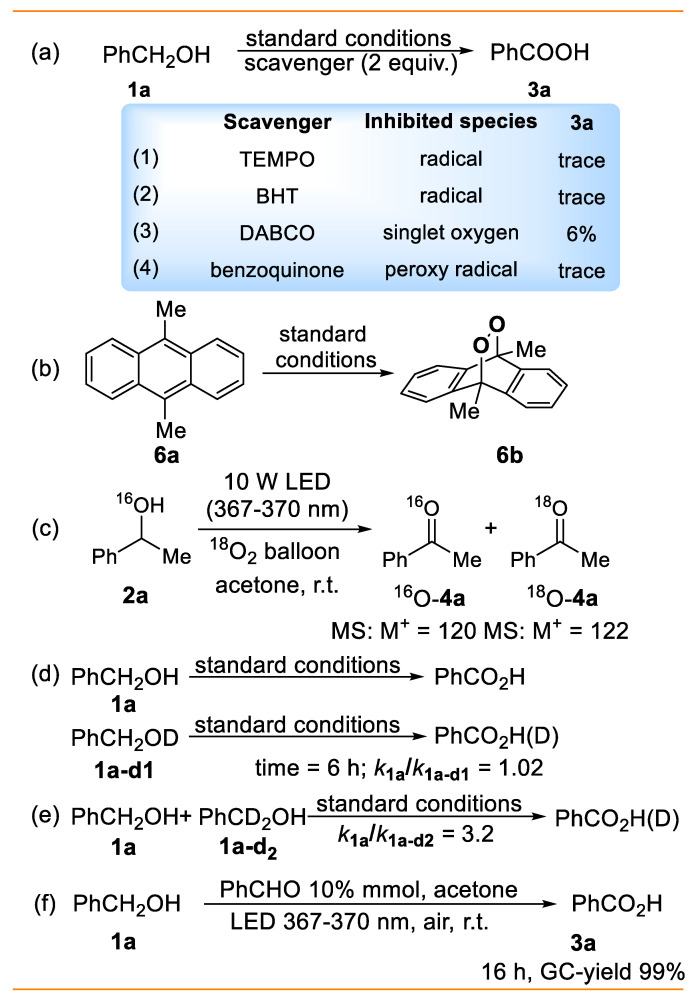
Control experiments (**a**) Radical trapped experiment; (**b**) Role of oxidant; (**c**) ^18^O_2_-labeling experiment; (**d**) Preparation of benzyl alcohol-OD; (**e**) Preparation of α, α-dideuteriobenzyl alcohol; (**f**) Catalytic experiment.

To further monitor the mechanism of the oxidation method, several control experiments were performed under light illumination (Scheme 4). First, the oxidation was performed with benzyl alcohols as starting materials, and the radical scavengers TEMPO or BHT were added. Only a trace amount of target product was produced ((1), (2) in Scheme 4a), which indicated that free radicals may have been generated during oxidation. Subsequently, we conducted an oxygenation with a singlet oxygen scavenger, 1,4-diazabicyclo [2.2.2]octane (DABCO), under the standard reaction conditions as oxygen molecules can be activated to singlet oxygen by photocatalysts ((3) in Scheme 4a). Oxidation only afforded benzoic acid (**3a**) in a 6% yield. When the reaction was trapped by the peroxy radical scavenger benzoquinone, the reaction process was also severely hampered and only a trace amount of desired product was observed ((4) in Scheme 4a). Subsequently, 9,10-dimethylanthracene (**4a**) underwent [4+2] cycloaddition under standard reaction conditions to achieve the target product (Scheme 4b), which further demonstrated that the oxidation of alcohols to carboxylic acid results from singlet oxygen. To further demonstrate this, electron paramagnetic resonance (EPR) experiments (see Supplementary Materials) were performed with benzyl alcohols in acetone under the standard reaction conditions, and both singlet oxygen and peroxy radical signals were trapped, confirming that singlet oxygen and peroxy radical mechanisms were involved in the oxidation system. We executed the oxidation reaction with ^18^O_2_ as the sole oxidant and found that a mixture of ^16^O-labeled and ^18^O-labeled ketone (**4a**) was produced (Scheme 4c), which demonstrated that both atmospheric oxygen and oxygen atoms of the C(sp3)–O bond of the alcohol were involved in the formation of the carbonyl group. To further investigate the rate determining step of the reaction system, the kinetic isotope effect of alcoholic O–H/D bonds and benzylic C(sp3)–H/D bonds were studied. The oxidation reaction was conducted with phenylmethanol (**1a**) and phenylmethanol-d1 (**1a**-**d1**) as the starting materials, and the k_1a_/k_1a–d1_ value was 1.02 (Scheme 4d). A mixture of phenylmethanol (**1a**) and phenylmethanol-d2 (**1a**-**d2**) was also oxidized, and the intermolecular k_H_/k_D_ value was 3.2 (Scheme 4e). Based on the above data, we can infer that the cleavage of the benzylic C(sp3)–H bond determines the rate of the reaction system. Considering that the oxidation rate may involve the intermediate benzaldehyde (**7a**) formed from the oxidation of benzyl alcohols, we attempted to add a small quantity of benzaldehyde to the initial reactive reactants (Scheme 4f). The target products were obtained in a 99% GC yield within 16 h, a shorter reaction time than for previous experiments, indicating that benzaldehyde acts as a catalyst for the reaction system.

**Scheme 5:**
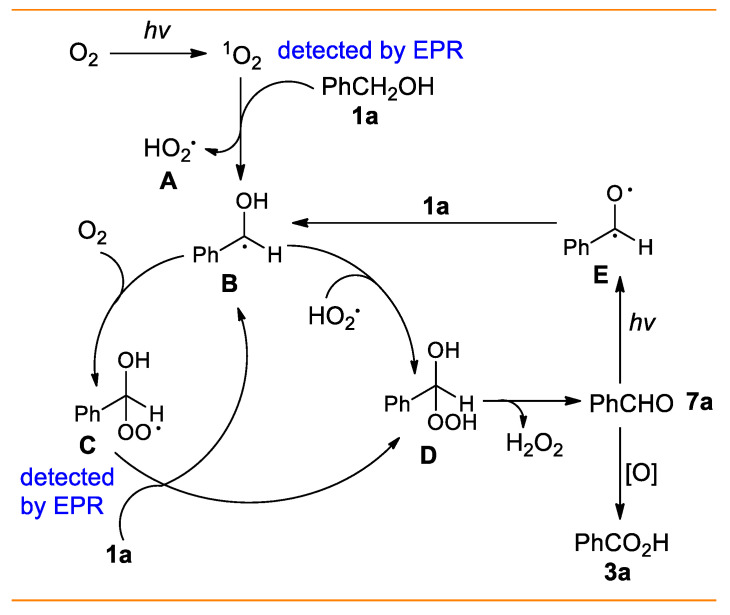
Proposed mechanism.

Based on the aforementioned results and reports in the literatures [53,54,55,56], a plausible mechanism for the photocatalytic oxygenation of alcohols to corresponding acids or ketones is proposed in Scheme 5. First, the ground-state triplet O_2_ tends to form singlet oxygen under light irradiation (see EPR experiments in the Supplementary Materials), subsequently extracting an electron from benzyl alcohol (**1a**), which initiates the formation of hydroperoxyl radical **A** and carbon-centered radical **B**. Then, the carbon-centered radical **B** transforms into peroxy radical **C** (see EPR experiments in the Supplementary Materials) under O_2_ conditions, and **C** reacts with benzyl alcohol (**1a**) to afford the peroxy compound **D**. The peroxy compound **D** was also obtained by the reaction of hydroperoxyl radical **A** and carbon-centered radical **B**. Subsequently, the peroxy compound **D** removes H_2_O_2_ and produces benzaldehyde. Benzaldehyde functions as a photocatalyst and transforms into a photoexcited intermediate **E**, which promotes the generation of carbon-centered radical **B** by extracting an electron from benzyl alcohol, thereby accelerating the rate of oxidation. Finally, the target product, benzoic acid (**3a**), was obtained via the oxidation of benzaldehyde.

## 3. Experimental Section

### 3.1. General Information

Commercially available starting materials were purchased and used without further purification. GC–MS was performed on a Shimadzu GC-MS 2010 (Kyoto, Japan). ^1^H NMR spectra were recorded on 400 MHz and referenced to the internal solvent signals (^1^H NMR: CDCl_3_ 7.26 ppm, ^1^H NMR: DMSO 2.50 ppm). ^13^C NMR spectra were recorded on 101 MHz spectrometers referenced to the internal solvent signals (^13^C NMR: CDCl_3_ 77.0 ppm, ^13^C NMR: 40.0 ppm). The peak information was described as brs = broad singlet, m = multiplet, q = quartet, t = triplet, d = doublet, and s = singlet. A Biotage Isolera four instrument (Tokyo, Japan) was used to purify (**4a**–**4e**, **4h**–**4j**, **4n,** and **5b**).

### 3.2. Typical Procedure for the Synthesis of Benzoic Acid (***3a***)

A mixture of phenylmethanol **1a** (0.5 mmol) and acetone (1.0 mL) was added to a 10 mL quartz tube with an air balloon at room temperature under the irradiation of 10 W LED lamps (367–370 nm) for 24 h. The progress was monitored by TLC or GC–MS. Upon completion, the mixture was cooled down to room temperature and transferred into a 10 mL heart-shaped bottle and concentrated in vacuum to obtain the crude products. Subsequently, a mixture of ethyl acetate and petroleum ether (1:60) was carefully drip-added to the crude product for recrystallization. After the crude product was completely dissolved, the solution was cooled to room temperature and an appropriate amount of cold petroleum ether was added under an ice bath to precipitate the product. Afterwards, the mixture was centrifuged and dried to obtain the benzoic acid **3a**.

### 3.3. Characterization Data of Products ***3a***–***3y***, ***4a***–***4s***, and ***5a***–***5d***

*Benzoic acid* (**3a**) [57]: White solid (58 mg, 95%); ^1^H NMR (400 MHz, DMSO-*d6*) δ 12.96 (s, 1H), 7.95 (d, *J* = 7.2 Hz, 2H), 7.62 (t, *J* = 7.2 Hz, 1H), 7.49 (t, *J* = 7.6 Hz, 2H); ^13^C NMR (101 MHz, DMSO-*d6*) δ 167.8, 133.4, 131.3, 129.8, 129.1.

*[1,1′-biphenyl]-4-carboxylic acid* (**3b**) [58]: White solid (94 mg, 95%); ^1^H NMR (400 MHz, DMSO-*d6*) δ 13.00 (s, 1H), 8.03 (d, *J* = 8.4 Hz, 2H), 7.80 (d, *J* = 8.4 Hz, 2H), 7.73 (d, *J* = 8.4 Hz, 2H), 7.50 (t, *J* = 7.6 Hz, 2H), 7.42 (t, *J* = 7.6 Hz, 1H); ^13^C NMR (101 MHz, DMSO-*d6*) δ 167.7, 144.8, 139.5, 130.5, 130.1, 129.6, 128.8, 127.5, 127.3.

*4-methylbenzoic acid* (**3c**) [57]: White solid (59 mg, 87%); ^1^H NMR (400 MHz, DMSO-*d6*) δ 12.80 (s, 1H), 7.83 (d, *J* = 8.4 Hz, 2H), 7.29 (d, *J* = 8.4 Hz, 2H), 2.35 (s, 3H); ^13^C NMR (101 MHz, DMSO-*d6*) δ 167.9, 143.6, 129.9, 129.7, 128.6, 21.7.

*4-isopropylbenzoic acid* (**3d**) [57]: White solid (75 mg, 90%); ^1^H NMR (400 MHz, DMSO-*d6*) δ 12.81 (s, 1H), 7.87 (d, J = 8.4 Hz, 2H), 7.36 (d, *J* = 8.4 Hz, 2H), 3.00–2.89 (m, 1H), 1.21 (d, *J* = 7.2 Hz, 6H); ^13^C NMR (101 MHz, DMSO-*d6*) δ 154.1, 130.0, 127.0, 34.0, 24.1.

*4-(tert-butyl)benzoic acid* (**3e**) [59]: White solid (82 mg, 92%); ^1^H NMR (400 MHz, DMSO-*d6*) δ 12.79 (s, 1H), 7.87 (d, *J* = 8.4 Hz, 2H), 7.50 (d, *J* = 8.4 Hz, 2H), 1.28 (s, 9H); ^13^C NMR (101 MHz, DMSO-*d6*) δ 167.8, 156.3, 129.7, 128.5, 125.9, 35.3, 31.4.

*4-(trifluoromethoxy)benzoic acid* (**3f**) [60]: Slight yellow solid (94 mg, 91%); ^1^H NMR (400 MHz, DMSO-*d6*) δ 13.19 (s, 1H), 8.06 (d, *J* = 8.4 Hz, 2H), 7.46 (d, *J* = 8.0 Hz, 2H); ^13^C NMR (101 MHz, DMSO-*d6*) δ 166.7, 152.0, 132.2, 130.4, 121.2, 120.5 (q, *J* = 255.9 Hz). ^19^F NMR (376 MHz, CDCl_3_) δ −56.7.

*4-fluorobenzoic acid* (**3g**) [57]: White solid (66 mg, 94%); ^1^H NMR (400 MHz, DMSO-*d6*) δ 13.06 (s, 1H), 8.02–7.98 (m, 2H), 7.34–7.29 (m, 2H); ^13^C NMR (101 MHz, DMSO-*d6*) δ 166.9, 165.4 (d, *J* = 249.0 Hz), 132.7 (d, *J* = 9.4 Hz), 127.9 (d, *J* = 2.8 Hz), 116.2 (d, *J* = 21.9 Hz); ^19^F NMR (376 MHz, DMSO-*d6*) δ −106.9.

*4-chlorobenzoic acid* (**3h**) [57]: White solid (73 mg, 93%); ^1^H NMR (400 MHz, DMSO-*d6*) δ 13.20 (s, 1H), 7.94 (d, *J* = 8.8 Hz, 2H), 7.57 (d, *J* = 8.8 Hz, 2H); ^13^C NMR (101 MHz, DMSO-*d6*) δ 167.0, 138.3, 131.7, 130.1, 129.3.

*4-bromobenzoic acid* (**3i**) [61]: White solid (95 mg, 95%); ^1^H NMR (400 MHz, DMSO-*d6*) δ 13.19 (s, 1H), 7.86 (d, *J* = 8.4 Hz, 2H), 7.70 (d, *J* = 8.4 Hz, 2H); ^13^C NMR (101 MHz, DMSO-*d6*) δ 167.1, 132.2, 131.8, 130.5, 127.4.

*4-cyanobenzoic acid* (**3j**) [58]: Gray white solid (55 mg, 75%); ^1^H NMR (400 MHz, DMSO-*d6*) δ 13.28 (s, 1H), 8.06 (s, 2H), 8.00 (s, 2H), ^13^C NMR (101 MHz, DMSO-*d6*) δ 195.9, 133.6, 118.7, 115.5.

*4-(methoxycarbonyl)benzoic acid* (**3k**) [60]: White solid (84 mg, 93%); ^1^H NMR (400 MHz, DMSO-*d6*) δ 13.36 (s, 1H), 8.05 (s, 4H), 3.87 (s, 3H); ^13^C NMR (101 MHz, DMSO-*d6*) δ 167.1, 166.1, 135.3, 133.7, 130.1, 129.9, 53.0.

*2-chlorobenzoic acid* (**3l**) [62]: White solid (73 mg, 94%); ^1^H NMR (400 MHz, DMSO-*d6*) δ 13.37 (s, 1H), 7.78 (d, *J* = 7.6 Hz, 1H), 7.55–7.50 (m, 2H), 7.45–7.39(m, 1H); ^13^C NMR (101 MHz, DMSO-*d6*) δ 167.3, 133.1, 132.1, 132.0, 131.3, 131.1, 127.7.

*2-methylbenzoic acid* (**3m**) [60]: White solid (58 mg, 86%); ^1^H NMR (400 MHz, DMSO-*d6*) δ 12.79 (s, 1H), 7.80 (d, *J* = 8.0 Hz, 1H), 7.43–7.39 (m, 1H), 7.26(t, *J* = 7.6 Hz, 2H); ^13^C NMR (101 MHz, DMSO-*d6*) δ 169.2, 139.5, 132.2, 132.0, 131.0, 130.7, 126.3, 21.8.

*3-chlorobenzoic acid* (**3n**) [60]: White solid (72 mg, 92%); ^1^H NMR (400 MHz, DMSO-*d6*) δ 13.34 (s, 1H), 7.90–7.88 (m, 2H), 7.70–7.67 (m, 1H), 7.53 (t, *J* = 8.0 Hz, 1H); ^13^C NMR (101 MHz, DMSO-*d6*) δ 166.6, 133.9, 133.4, 133.2, 131.2, 129.4, 128.4.

*3-methylbenzoic acid* (**3o**) [60]: Slight yellow solid (55 mg, 81%); ^1^H NMR (400 MHz, DMSO-*d6*) δ 12.89 (s, 1H), 7.76–7.73 (m, 2H), 7.44–7.36 (m, 2H), 2.36 (s, 3H); ^13^C NMR (101 MHz, DMSO-*d6*) δ 167.9, 138.4, 134.0, 131.2, 130.2, 129.0, 127.0, 21.3.

*3,4-dichlorobenzoic acid* (**3p**) [62]: White solid (92 mg, 96%); ^1^H NMR (400 MHz, DMSO-*d6*) δ 13.50 (s, 1H), 8.04 (d, *J* = 2.0 Hz, 1H), 7.87 (dd, *J* = 8.4, 2.0 Hz, 1H), 7.76 (d, *J* = 8.4 Hz, 1H); ^13^C NMR (101 MHz, DMSO-*d6*) δ 165.9, 136.3, 132.0, 131.9, 131.5, 131.5, 129.8.

*terephthalic acid* (**3q**) [57]: White solid (59 mg, 71%); ^1^H NMR (400 MHz, DMSO-*d6*) δ 13.29 (s, 2H), 8.03 (s, 4H); ^13^C NMR (101 MHz, DMSO-*d6*) δ 167.3, 135.1, 130.1.

*1-naphthoic acid* (**3r**) [57]: White solid (65 mg, 78%); ^1^H NMR (400 MHz, DMSO-*d6*) δ 13.17 (s, 1H), 8.86 (d, *J* = 8.4 Hz, 1H), 8.17–8.14 (m, 2H), 8.02 (d, *J* = 7.6 Hz, 1H), 7.66–7.57 (m, 3H); ^13^C NMR (101 MHz, DMSO-*d6*) δ 169.2, 134.0, 133.5, 131.2, 130.4, 129.1, 128.2, 128.1, 126.7, 126.0, 125.4.

*anthracene-9-carboxylic acid* (**3s**) [63]: Yellow solid (97 mg, 87%); ^1^H NMR (400 MHz, DMSO-*d6*) δ 13.94 (s, 1H), 8.73 (s, 1H), 8.16 (d, *J* = 8.4 Hz, 2H), 8.05 (d, *J* = 8.4 Hz, 2H), 7.64–7.57 (m, 4H); ^13^C NMR (101 MHz, DMSO-*d6*) δ 170.7, 131.0, 130.2, 129.1, 128.8, 127.6, 127.4, 126.2, 125.4.

*isonicotinic acid* (**3t**) [62]: White solid (45 mg, 74%); ^1^H NMR (400 MHz, DMSO-*d6*) δ 13.64 (s, 1H), 8.78 (dd, *J* = 4.4, 1.6 Hz, 2H), 7.81 (dd, *J* = 4.4 1.6 Hz, 2H); ^13^C NMR (101 MHz, DMSO-*d6*) δ 166.7, 151.1, 138.6, 123.3.

*thiophene-2-carboxylic acid* (**3u**) [57]: Gray white solid (54 mg, 85%); ^1^H NMR (400 MHz, DMSO-*d6*) δ 13.06 (s, 1H), 7.88 (dd, *J* = 4.8, 1.2 Hz, 1H), 7.73 (dd, *J* = 3.6, 1.2 Hz, 1H), 7.18 (dd, *J* = 4.8, 3.6 Hz, 1H); ^13^C NMR (101 MHz, DMSO-*d6*) δ 163.4, 135.2, 133.8, 133.7, 128.7.

*isobenzofuran-1(3H)-one* (**3v**) [57]: White solid (48 mg, 71%); ^1^H NMR (400 MHz, CDCl_3_) δ 7.92 (d, *J* = 8.0 Hz, 1H), 7.70–7.67 (m, 1H), 7.55–7.49 (m, 2H), 5.32 (s, 2H); ^13^C NMR (101 MHz, CDCl_3_) δ 171.1, 146.5, 134.0, 129.0, 125.7, 125.7, 122.1, 69.6.

*2-((3-(trifluoromethyl)phenyl)amino)nicotinic acid* (**3y**) [64]: Gray white solid (96 mg, 68%); ^1^H NMR (400 MHz, DMSO-*d6*) δ 10.64 (s, 1H), 8.42 (dd, *J* = 4.8, 2.0 Hz, 1H), 8.28–8.26 (m, 2H), 7.84 (d, *J* = 8.0 Hz, 1H), 7.50 (t, *J* = 8.0 Hz, 1H), 7.30 (d, *J* = 8.0 Hz, 1H), 6.92 (dd, *J* = 7.6, 4.8 Hz, 1H); ^13^C NMR (101 MHz, DMSO-*d6*) δ 169.5, 155.7, 153.0, 141.2 (d, *J*C-F = 7.5 Hz), 130.2, 130.1 (d, *J*C-F = 93.5 Hz), 129.9, 124.8 (q, *J*C-F = 270.7 Hz), 123.9, 118.7 (*J*C-F = 3.9 Hz), 116.2 (*J*C-F = 4.0 Hz), 115.3, 108.9; ^19^F NMR (376 MHz, CDCl_3_) δ −61.2.

*acetophenone* (**4a**) [65]: Colorless liquid (55 mg, 91%); ^1^H NMR (400 MHz, DMSO-*d6*) δ 7.93 (d, *J* = 8.0 Hz, 2H), 7.53 (t, *J* = 7.8 Hz, 1H), 7.43 (t, *J* = 8.0 Hz, 2H), 2.57 (s, 3H); ^13^C NMR (101 MHz, DMSO-*d6*) δ 198.0, 136.9, 133.0, 128.4, 128.1, 26.4.

*1-(p-tolyl)ethanone* (**4b**) [65]: Colorless liquid (55 mg, 82%); ^1^H NMR (400 MHz, DMSO-*d6*) δ 7.84 (d, *J* = 8.0 Hz, 2H), 7.24 (d, *J* = 8.0 Hz, 2H), 2.56 (s, 3H), 2.39 (s, 3H); ^13^C NMR (101 MHz, DMSO-*d6*) δ 197.8, 143.8, 134.6, 129.1, 128.3, 26.4, 21.5.

*1-(4-fluorophenyl)ethanone* (**4c**) [65]: Colorless liquid (63 mg, 91%); ^1^H NMR (400 MHz, DMSO-*d6*) δ 7.99–7.93 (m, 2H), 7.13–7.07 (m, 2H), 2.57 (s, 3H); ^13^C NMR (101 MHz, DMSO-*d6*) δ 196.4, 165.7 (d, *J*C-F = 253.1), 133.5, 130.9 (d, *J*C-F = 9.3 Hz), 115.6 (d, *J*C-F = 21.7), 26.4. ^19^F NMR (376 MHz, CDCl_3_) δ −105.3.

*1-(4-chlorophenyl)ethanone* (**4d**) [65]: Colorless liquid (74 mg, 96%);^1^H NMR (400 MHz, DMSO-*d6*) δ 7.87 (d, *J* = 8.4 Hz, 2H), 7.40 (d, *J* = 8.4 Hz, 2H), 2.56 (s, 3H); ^13^C NMR (101 MHz, DMSO-*d6*) δ 196.7, 139.5, 135.3, 129.6, 128.8, 26.5.

*1-(4-bromophenyl)ethanone* (**4e**) [65]: Colorless liquid (97 mg, 97%); ^1^H NMR (400 MHz, DMSO-*d6*) δ 7.82 (d, *J* = 8.4 Hz, 2H), 7.60 (d, *J* = 8.4 Hz, 2H), 2.58 (s, 3H); ^13^C NMR (101 MHz, DMSO-*d6*) δ 197.0, 135.8, 131.9, 129.8, 128.3, 26.5.

*benzophenone* (**4f**) [66]: White solid (87 mg, 96%); ^1^H NMR (400 MHz, DMSO-*d6*) δ 7.80 (d, *J* = 7.2 Hz, 4H), 7.57 (t, *J* = 7.2 Hz, 2H), 7.47 (t, *J* = 7.6 Hz, 4H); ^13^C NMR (101 MHz, DMSO-*d6*) δ 196.6, 137.4, 132.3, 129.9, 128.1.

*phenyl(p-tolyl)methanone* (**4g**) [67]: White solid (88 mg, 90%); ^1^H NMR (400 MHz, DMSO-*d6*) δ 7.80–7.77 (m, 2H), 7.73 (d, J = 8.0 Hz, 2H), 7.60–7.56 (m, 1H), 7.47 (t, *J* = 7.6 Hz, 2H), 7.28 (d, *J* = 8.0 Hz, 2H), 2.44 (s, 3H); ^13^C NMR (101 MHz, DMSO-*d6*) δ 196.5, 143.2, 137.9, 134.8, 132.1, 130.3, 129.9, 128.9, 128.2, 21.6.

*(4-fluorophenyl)(phenyl)methanone* (**4h**) [67]: Colorless liquid (93 mg, 93%); ^1^H NMR (400 MHz, DMSO-*d6*) δ 7.87–7.82 (m, 2H), 7.78–7.76 (m, 2H), 7.62–7.58 (m, 1H), 7.19–7.13 (m, 2H); ^13^C NMR (101 MHz, DMSO-*d6*) δ 195.3, 165.4 (d, *J*C-F = 252.6 Hz), 137.5, 133.8, 133.7, 132.7, 132.6, 132.5, 129.9, 128.3, 115.5, 115.3. ^19^F NMR (376 MHz, CDCl_3_) δ −105.9.

*(4-chlorophenyl)(phenyl)methanone* (**4i**) [68]: Colorless liquid (103 mg, 95%); ^1^H NMR (400 MHz, DMSO-*d6*) δ 7.78–7.74 (m, 4H), 7.62–7.58 (m, 1H), 7.51–7.44 (m, 4H); ^13^C NMR (101 MHz, DMSO-*d6*) δ 195.5, 138.9, 137.2, 135.8, 132.6, 131.4, 129.9, 128.6, 128.4.

*(4-bromophenyl)(phenyl)methanone* (**4j**) [68]: Colorless liquid (123 mg, 94%); ^1^H NMR (400 MHz, DMSO-*d6*) δ 7.78–7.76 (m, 2H), 7.69–7.67 (m, 2H), 7.64–7.61 (m, 3H), 7.49 (t, *J* = 7.6 Hz, 2H); ^13^C NMR (101 MHz, DMSO-*d6*) δ 195.7, 137.1, 136.3, 132.7, 131.6, 131.6, 129.9, 128.4, 127.5.

*2-chloro-1-phenylethanone* (**4k**) [69]: White solid (70 mg, 90%); ^1^H NMR (400 MHz, DMSO-*d6*) δ 7.96 (d, *J* = 7.6 Hz, 2H), 7.62 (t, *J* = 7.6 Hz, 2H), 7.50 (t, *J* = 7.6 Hz, 2H), 4.72 (s, 2H); ^13^C NMR (101 MHz, DMSO-*d6*) δ 191.0, 134.2, 134.0, 128.9, 128.5, 46.0.

*2-bromo-1-phenylethanone* (**4l**) [69]: Grayish white solid (88 mg, 88%); ^1^H NMR (400 MHz, DMSO-*d6*) δ 7.98 (d, *J* = 8.4 Hz, 2H), 7.62 (t, *J* = 7.6 Hz, 1H), 7.50 (t, *J* = 7.6 Hz, 2H), 4.46 (s, 2H); ^13^C NMR (101 MHz, DMSO-*d6*) δ 191.3, 133.9, 133.9, 128.9, 128.8, 30.9.

*3-chloro-1-phenylpropan-1-one* (**4m**) [70]: Grayish white solid (70 mg, 83%); ^1^H NMR (400 MHz, DMSO-*d6*) δ 7.95 (d, *J* = 7.6 Hz, 2H), 7.59 (t, *J* = 7.6 Hz, 1H), 7.47 (t, *J* = 7.6 Hz, 2H), 3.92 (t, *J* = 6.8 Hz, 2H), 3.45 (t, *J* = 6.8 Hz, 2H); ^13^C NMR (101 MHz, DMSO-*d6*) δ 196.6, 136.3, 133.5, 128.7, 128.0, 41.2, 38.6.

*benzoyl cyanide* (**4n**) [71]: Colorless liquid (60 mg, 92%); ^1^H NMR (400 MHz, DMSO-*d6*) δ 8.14 (d, *J* = 7.6 Hz, 2H), 7.79 (t, *J* = 7.6 Hz, 1H), 7.61 (t, *J* = 7.6 Hz, 2H); ^13^C NMR (101 MHz, DMSO-*d6*) δ 167.8, 136.8, 133.3, 130.4, 129.5, 112.7.

*9H-fluoren-9-one* (**4o**) [66]: White solid (82 mg, 91%); ^1^H NMR (400 MHz, DMSO-*d6*) δ 7.65 (d, *J* = 7.6 Hz, 2H), 7.52–7.46 (m, 4H), 7.31–7.27 (m, 2H); ^13^C NMR (101 MHz, DMSO-*d6*) δ 193.9, 144.4, 134.7, 134.1, 129.0, 124.3, 120.3.

*9H-xanthen-9-one* (**4p**) [72]: Grayish white solid (88 mg, 90%); ^1^H NMR (400 MHz, DMSO-*d6*) δ 8.35 (dd, *J* = 8.0, 1.6 Hz, 2H), 7.73 (td, *J* = 7.6, 1.6 Hz, 2H), 7.49 (d, *J* = 8.0 Hz, 2H), 7.40–7.36 (m, 2H); ^13^C NMR (101 MHz, DMSO-*d6*) δ 177.2, 156.1, 134.8, 126.7, 123.9, 121.8, 118.0.

*9H-thioxanthen-9-one* (**4q**) [73]: Light yellow solid (93 mg, 88%); ^1^H NMR (400 MHz, DMSO-*d6*) δ 8.62 (dd, *J* = 8.0, 1.2 Hz, 2H), 7.65–7.57 (m, 4H), 7.51–7.47 (m, 2H); ^13^C NMR (101 MHz, DMSO-*d6*) δ 180.0, 137.3, 132.3, 129.9, 129.2, 126.3, 126.0.

*methyl 2-(1-(4-acetylbenzoyl)-5-methoxy-2-methyl-1H-indol-3-yl)acetate* (**4r**) [44]: Grayish white solid (148 mg, 78%); ^1^H NMR (400 MHz, DMSO-*d6*) δ 8.06 (d, *J* = 8.4 Hz, 2H), 7.79 (d, *J* = 8.4 Hz, 2H), 6.96 (d, *J* = 2.4 Hz, 1H), 6.85 (d, *J* = 9.2 Hz, 1H), 6.65 (dd, *J* = 9.2, 2.4 Hz, 1H), 3.83 (s, 3H), 3.71 (s, 3H), 3.67 (s, 2H), 2.68 (s, 3H), 2.36 (s, 3H); ^13^C NMR (101 MHz, DMSO-*d6*) δ 197.3, 171.3, 168.5, 156.2, 139.8, 139.6, 135.9, 130.8, 130.7, 129.7, 128.6, 115.1, 112.9, 111.7, 101.4, 55.7, 52.2, 30.1, 26.9, 13.5.

*isopropyl 2-(4-(4-acetylbenzoyl)phenoxy)-2-methylpropanoate* (**4s**) [44]: White solid (153 mg, 83%); ^1^H NMR (400 MHz, DMSO-*d6*) δ 8.04 (d, *J* = 8.0 Hz, 2H), 7.80 (d, *J* = 8.0 Hz, 2H), 7.75 (d, *J* = 8.0 Hz, 2H), 6.86 (d, *J* = 8.4 Hz, 2H), 5.11–5.05 (m, 1H), 2.66 (s, 3H), 1.66 (s, 6H), 1.20 (d, *J* = 6.0 Hz, 6H); ^13^C NMR (101 MHz, DMSO-*d6*) δ 197.6, 194.7, 173.0, 160.0, 142.0, 139.2, 132.1, 129.9, 129.7, 128.1, 117.2, 79.4, 69.4, 26.8, 25.3, 21.5, 1.0.

*benzohydrazide* (**5a**) [49]: Grayish white solid (55 mg, 81%); ^1^H NMR (400 MHz, DMSO-*d6*) δ 9.79 (s, 1H), 7.83 (d, *J* = 7.2 Hz, 2H), 7.52–7.42 (m, 3H), 4.51 (s, 2H); ^13^C NMR (101 MHz, DMSO-*d6*) δ 166.5, 133.8, 131.6, 128.8, 127.5.

*phenyl benzoate* (**5b**) [50]: Colorless liquid (77 mg, 78%); ^1^H NMR (400 MHz, DMSO-*d6*) δ 8.23 (d, *J* = 7.2 Hz, 2H), 7.65 (t, *J* = 7.2 Hz, 1H), 7.53 (t, *J* = 7.6 Hz, 2H), 7.45 (t, *J* = 8.0 Hz, 2H), 7.31–7.23 (m, 3H); ^13^C NMR (101 MHz, DMSO-*d6*) δ 165.2, 150.9, 133.5, 130.1, 129.5, 129.5, 128.5, 125.9, 121.7.

*acetophenone oxime* (**5c**) [51]: White solid (51 mg, 75%); ^1^H NMR (400 MHz, DMSO-*d6*) δ 10.30 (s, 1H), 6.74–6.72 (m, 2H), 6.48–6.43 (m, 3H), 1.24 (s, 3H); ^13^C NMR (101 MHz, DMSO-*d6*) δ 153.4, 137.5, 129.1, 128.9, 126.1, 12.1.

*(E)-chalcone* (**5d**) [52]: Light yellow solid (80 mg, 77%); ^1^H NMR (400 MHz, DMSO-*d6*) δ 8.03 (d, *J* = 7.2 Hz, 2H), 7.82 (d, *J* = 16.0 Hz, 1H), 7.66–7.49 (m, 6H), 7.46–7.41 (m, 3H); ^13^C NMR (101 MHz, DMSO-*d6*) δ 190.5, 144.8, 138.2, 134.8, 132.8, 130.5, 128.9, 128.6, 128.5, 128.4, 122.1.

## 4. Conclusions

The results of this study present a highly efficient and practical system for the transformtion of aromatic alcohols to desired acids or ketones via light irradiation under external catalyst-, additive-, and base-free conditions. The following are notable characteristics of the developed system: (1) the photoreaction system is economical and environmentally friendly, owing to the use of air as a terminal oxidant and the reaction intermediate aldehydes as a photocatalyst. (2) A one-pot sequential transformation from aromatic alcohols to carboxylic acids or ketones was performed successfully in excellent yield, which exhibited a wide substrate scope and could also be applied on a large scale. (3) The most desired products are easily obtained via recrystallization and separation from low-boiling reaction medium acetone. More importantly, the crude products were available for subsequent direct synthesis without further purification. We believe that the developed method provides a practical light-initiated oxidation approach and could attract interest from those working in pharmaceutical chemistry and synthetic natural products chemistry.

## Figures and Tables

**Table 1:** Reaction conditions *^a^*.

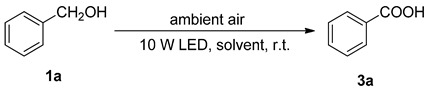				
Entry	Light Source (nm)	Solvent *^b^*	Atmosphere	Yield *^c^* (%)
1	390–395	MeCN	Oxygen	63
2	390–395	EtOH	Oxygen	54
3	390–395	1,4-dioxane	Oxygen	21
4	390–395	DCE	Oxygen	56
5	390–395	DMF	Oxygen	62
6	390–395	DMSO	Oxygen	63
7	390–395	Acetone	Oxygen	74
8	385–390	Acetone	Oxygen	75
9	380–385	Acetone	Oxygen	76
10	375–380	Acetone	Oxygen	80
11	370–375	Acetone	Oxygen	78
12	367–370	Acetone	Oxygen	98
13	365–367	Acetone	Oxygen	71
14	395–400	Acetone	Oxygen	64
15	400–405	Acetone	Oxygen	57
16	367–370	Acetone	Air	98
17	367–370	Acetone	N_2_	0
18		Acetone	air	0

*^a^* Reaction conditions: **1a** (0.5 mmol), solvent (1 mL), 24 h; *^b^* DCE: 1,2-dichloroethane; DMF: *N*,*N*-dimethylacetamide; DMSO dimethyl sulfoxide; *^c^* Yields were estimated by GC–MS.

**Table 2:** Reaction scope *^a^*.

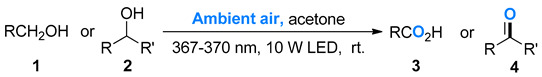				
	R			
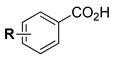	4-H	**3a**	95%	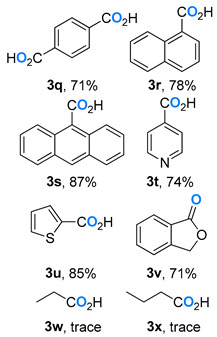
	4-Ph	**3b**	95%	
	4-Me	**3c**	87%	
	4-*^i^*Pr	**3d**	90%	
	4-*^t^*Bu	**3e**	92%	
	4-OCF_3_	**3f**	91%	
	4-F	**3g**	94%	
	4-Cl	**3h**	93%	
	4-Br	**3i**	95%	
	4-CN	**3j**	75%	
	4-CO_2_Me	**3k**	93%	
	2-Cl	**3l**	94%	
	2-Me	**3m**	86%	
	3-Cl	**3n**	92%	
	3-Me	**3o**	81%	
	3,4-*^dl^*-Cl	**3p**	96%	
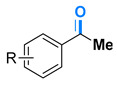	4-H	**4a**	91%	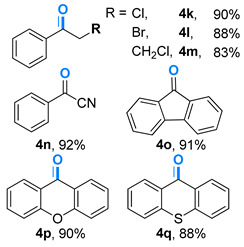
	4-Me	**4b**	82%	
	4-F	**4c**	91%	
	4-Cl	**4d**	96%	
	4-Br	**4e**	97%	
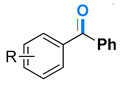	4-H	**4f**	96%	
	4-Me	**4g**	90%	
	4-F	**4h**	93%	
	4-Cl	**4i**	95%	
	4-Br	**4j**	94%	

*^a^* Reaction conditions: **1** or **2** (0.5 mmol), acetone (1 mL), 367–370 nm 10 W LED, air balloon, 24 h.

## Data Availability

The data presented in this study are available on request from the corresponding author.

## Supplementary Materials

The following supporting information can be downloaded at: https://www.mdpi.com/article/10.3390/molecules28073031/s1, Section S1: Experimental procedure; Section S2: Mechanism research; Figure S1: MS spectra of ^16^O-**4a** and ^18^O-**4a**; Figure S2. UV-Vis Spectroscopic; Figure S3: The X-band electron paramagnetic resonance (EPR) spectra of the singlet oxygen captured by TMPD; Figure S4: The X-band electron paramagnetic resonance (EPR) spectra of the peroxy radical captured by DMPO; Section S3: Comparison of methodology; Table S1: Comparison of reaction conditions and yields between this methodology and other schemes reported in the literature; Section S4: Copies of the ^1^H NMR and ^13^C NMR for compounds **3a**–**3y** and **4a**–**4s**. Section S5: References.

## Sample Availability

Samples of the compounds **3a**–**3y** and **4a**–**4s** are available from the authors.
