# Supplementary material for: External Catalyst- and Additive-Free Photo-Oxidation of Aromatic Alcohols to Carboxylic Acids or Ketones Using Air/O2
Source: Molecules. 2023 Mar 28;28(7):3031. doi: 10.3390/molecules28073031 (PMC10096038; doi:10.3390/molecules28073031)
Supplement: Supplementary file 1 [file molecules-28-03031-s001.zip › molecules-2266942-supplementary.pdf]

Supplementary Materials

External Catalyst- and Additive-Free Photo-Oxidation of Aromatic Alcohols to Carboxylic Acids or Ketones Using Air/O_2

Meng Xu [1], Jinhua Ou [1,*], Kejun Luo [2], Rongtao Liang [1], Jian Liu [1], Ni Li [1], Bonian Hu [1] and Kaijian Liu [1,*]

[1] School of Materials Science and Engineering, Hunan Institute of Technology, Hengyang 421002, China

[2] Analytical Testing Center, Changsha Research Institute of Mining and Metallurgy Co., Ltd., Changsha 410012, China

* Correspondence: oujinhua@hnu.edu.cn (J.O.); 2013001783@hnit.edu.cn (K.L.)

Table of Contents

S1.Experimental procedure S2

S2. Mechanism Research S4

S3. Comparison of methodology S10

S4. ^{1}H and ^{13}C NMR spectra of products S11

S5. References S60

S1. Experimental Procedure

(a) One-pot synthesis of benzohydrazide

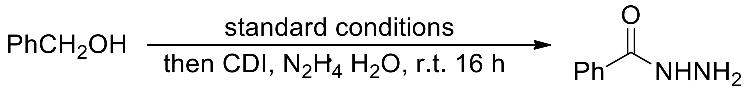

1a **5a**

A mixture of phenylmethanol **1a** (0.5 mmol) and acetone (1.0 mL) was added to a 10 mL quartz tube with an air balloon at room temperature under the irradiation of 10 W LED lamps (367 – 370 nm) for 24 h. Upon completion, CDI (106 mg, 0.65 mmol) was added. The mixture was stirred at room temperature with the dropwise addition of acetone (2 ml) solution containing $N_2H_4{\cdot}H_2O$ 80 % (95 mg, 1.5 mmol) over a period of 5 min. The mixture was magnetically stirred at room temperature for 16 hours. The reaction was quenched by adding water and extracted with EtOAc. The organic layer was washed with water and brine, dried over $MgSO_4$, filtered and evaporated under reduced pressure to afford the crude product， which was further purified by silica gel column chromatography with petroleum ether/ethyl acetate as eluent. The overall yield was 81 %.

(b) One-pot synthesis of phenyl benzoate

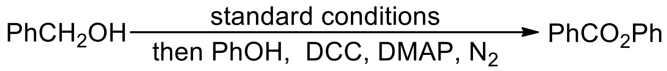

1a **5b**

A mixture of phenylmethanol **1a** (0.5 mmol) and acetone (1.0 mL) was added to a 10 mL quartz tube with an air balloon at room temperature under the irradiation of 10 W LED lamps (367 – 370 nm) for 24 h. Upon completion, phenol (0.5 mmol) was added under N_2 atmosphere, DCC (0.75 mmol) and DMAP (0.25 mmol) were slowly added and stirred for 8 hours at room temperature. The reaction was quenched by adding water and extracted with EtOAc. The organic layer was washed with water and brine, dried over $MgSO_4$, filtered and evaporated under reduced pressure to afford the crude product， which was further purified by silica gel column chromatography with petroleum ether/ethyl acetate as eluent. The overall yield was 78 %.

(c) One-pot synthesis of acetophenone oxime

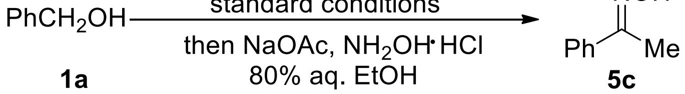

1a **5c**

A mixture of phenylmethanol **1a** (0.5 mmol) and acetone (1.0 mL) was added to a 10 mL quartz tube with an air balloon at room temperature under the irradiation of 10 W LED lamps (367 – 370 nm) for 24 h. Upon completion, hydroxylamine hydrochloride (0.8 mmol) and NaOAc (1.0 mmol) in 80% aqueous EtOH (20 mL) was

stirred at room temperature for 30 min. Then, the standard reaction conditions mixture was added to above solution and the reaction mixture was heated gently to reflux for 2 h. After the completion of reaction by TLC monitoring, the reaction mass was cooled to room temperature followed by removal of excess ethanol under vacuum. The crude product which was further purified by silica gel column chromatography with petroleum ether/ethyl acetate as eluent. The overall yield was 75 %.

(d) One-pot synthesis of (*E*)-chalcone

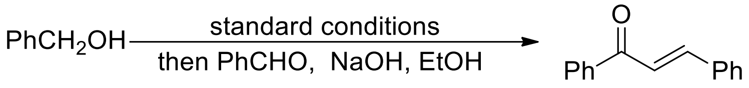

1a **5d**

A mixture of phenylmethanol **1a** (0.5 mmol) and acetone (1.0 mL) was added to a 10 mL quartz tube with an air balloon at room temperature under the irradiation of 10 W LED lamps (367 – 370 nm) for 24 h. Upon completion, benzaldehyde (0.5 mmol), EtOH (3 mL) , sodium hydroxide (0.05 mmol) was added and stirred for 5 hours at room temperature. The progress of the reaction was monitored by TLC. Upon completion of the reaction, the crude product was filtered and re-crystallised from ethanol. The overall yield was 77 %.

(e) Large-scale oxidation of 1a to 3a

A mixture of phenylmethanol **1a** (10.0 mmol) and acetone (20.0 mL) was added to a 50 mL round-bottomed flask with an air bag under room temperature with the irradiation of 10 W LED lamps (367 – 370 nm) for 54 h. The progress was monitored by TLC or GC-MS. Upon completion, the mixture was cooled down to room temperature and concentrated in vacuum to obtain the crude products. The crude product was recrystallized to give benzoic acid **3a** (recrystallization yield 84%, 1.03 g).

(f) Large-scale oxidation of 1i to 3i

A mixture of (4-bromophenyl)methanol **1i** (10.0 mmol) and acetone (20.0 mL) was added to a 50 mL round-bottomed flask with an air bag under room temperature with the irradiation of 10 W LED lamps (367 – 370 nm) for 60 h. The progress was monitored by TLC or GC-MS. Upon completion, the mixture was cooled down to room temperature and concentrated in vacuum to obtain the crude products. The crude product was recrystallized to give benzoic acid **3i** (recrystallization yield 86%, 1.73 g).

S2. Mechanism Research

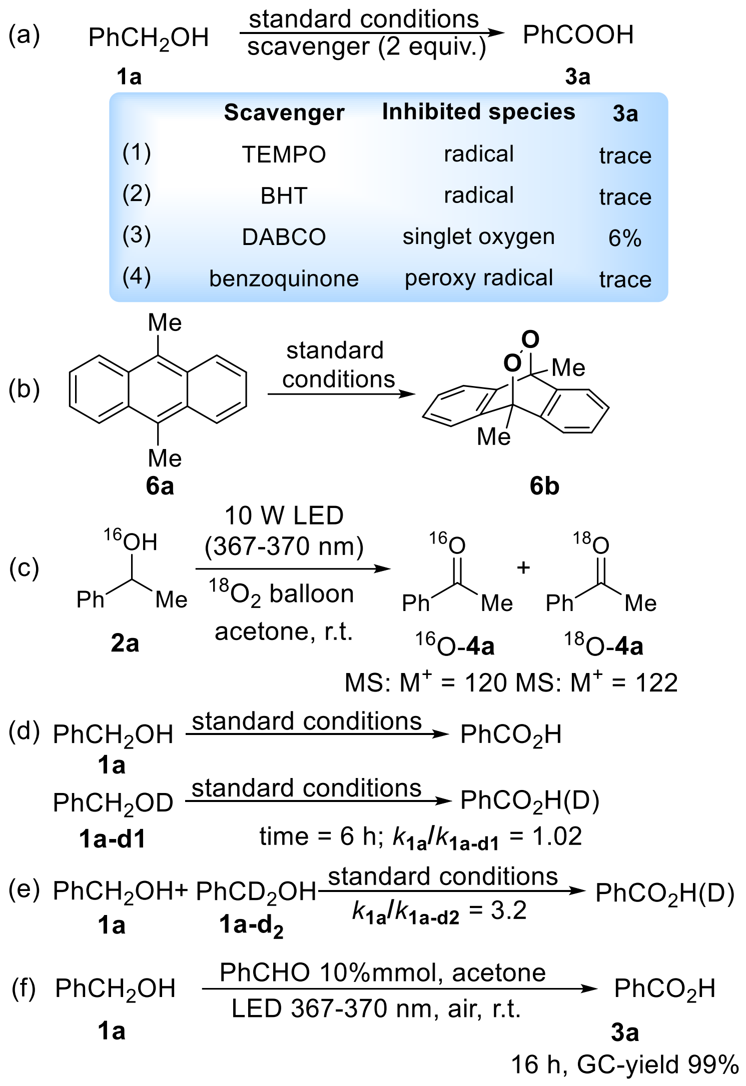

(a)-1 Radical trapped experiment (TEMPO):

A mixture of phenylmethanol **1a** (0.5 mmol), TEMPO (156 mg, 1.0 mmol) and acetone (1.0 mL) was added to a 10 mL quartz tube with an air balloon at room temperature under the irradiation of 10 W LED lamps (367 – 370 nm) for 24 h. GC-MS analysis of the reaction mixture showed that only a trace amount of **3a** was formed.

(a)-2 Radical trapped experiment (BHT):

A mixture of phenylmethanol **1a** (0.5 mmol), BHT (220 mg, 1 mmol) and acetone (1.0 mL) was added to a 10 mL quartz tube with an air balloon at room temperature under the irradiation of 10 W LED lamps (367 – 370 nm) for 24 h. GC-MS analysis of the reaction mixture showed that only a trace amount of **3a** was formed.

(a)-3 Radical trapped experiment (DABCO):

A mixture of phenylmethanol **1a** (0.5 mmol), 1,4-diazabicyclo[2.2.2]octane (DABCO, 113 mg, 1.0 mmol) and acetone (1.0 mL) was added to a 10 mL quartz tube with an air balloon at room temperature under the irradiation of 10 W LED lamps (367 – 370 nm) for 24

h. GC-MS analysis of the reaction mixture showed that only 6% yield of **3a** was obtained.

(a)-4 Radical trapped experiment (benzoquinone):

A mixture of phenylmethanol **1a** (0.5 mmol), 1,4- benzoquinone (108 mg, 1.0 mmol) and acetone (1.0 mL) was added to a 10 mL quartz tube with an air balloon at room temperature under the irradiation of 10 W LED lamps (367 – 370 nm) for 24 h. GC-MS analysis of the reaction mixture showed that only a trace amount of **3a** was formed.

(b) Role of oxidant:

A mixture of 9,10-dimethylanthracene **6a** (42.0 mg, 0.2 mmol) and acetone (0.5 mL) was added to a 10 mL quartz tube with an air balloon at room temperature under the irradiation of 10 W LED lamps (367 – 370 nm) for 24 h. The reaction was cooled down to room temperature and analyzed by MS showed **6b** product formation.

(c) $^{18}O_2$-labeling experiment:

A mixture of phenylmethanol **1a** (0.5 mmol) and acetone (1.0 mL) was added to a 10 mL quartz tube with an $^{18}O_2$ balloon at room temperature under the irradiation of 10 W LED lamps (367 – 370 nm) for 24 h. The reaction was cooled down to room temperature and the ^{16}O-**4a** and ^{18}O-**4a** was detected by GC-MS.

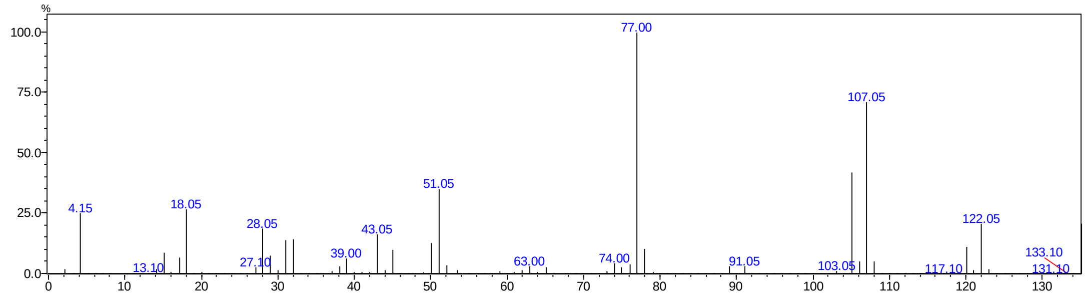

Figure S1. MS spectra of ^{16}O-**4a** and ^{18}O-**4a**

(d) Parallel experiment of KIE:

Preparation of benzyl alcohol-OD ($PhCH_2OD$) 1a-d1: In a 50 mL round-bottom flask benzyl alcohol **1a** (4.63 mmol, 500 mg) was taken in 5 ml D_2O. The mixture was stirred for 48 hours under room temperature. After concentrating in vacuum, the residue was purified by flash chromatography on a short sil-ica gel to afford 400 mg (98% of D) of $PhCH_2OD$[1].

A mixture of $PhCH_2OH$ (0.5 mmol) (or $PhCH_2OD$ (0.5 mmol)) and acetone (1.0 mL) was added to a 10 mL quartz tube with an air balloon at room temperature under the irradiation of 10 W LED lamps (367 – 370 nm) for 6 h. The reaction was cooled down to room temperature. The residual starting material ($PhCH_2OH$ or $PhCH_2OD$) was recovered by column chromatography on silica gel (200-300 mesh), the k_H/k_D = 1.02 was calculated by amount of substance of the isolated compounds of $PhCH_2OH$ **1a** and $PhCH_2OD$ **1a-d1**.

(e) Intermolecular competition experiment of KIE:

Preparation of α, α-dideuteriobenzyl alcohol ($PhCD_2OH$) 1a-d2: In a 50 mL round-bottom flask $LiAlD_4$ (150 mg, 3.6 mmol) was taken in THF (20 mL) at 0ºC and then, a solution of

methyl benzoate (5 mmol, 680 mg) was taken in THF (10 mL). The mixture was stirred for 2 hours at 0 ºC. The resulting solu-tion was quenched with HCl 1N and extracted with ether (3×20 mL), followed by concentrating in vacuum and the residue was purified by flash chromatography on a short silica gel to afford 118 mg (99% of D) of $PhCD_2OH$[1].

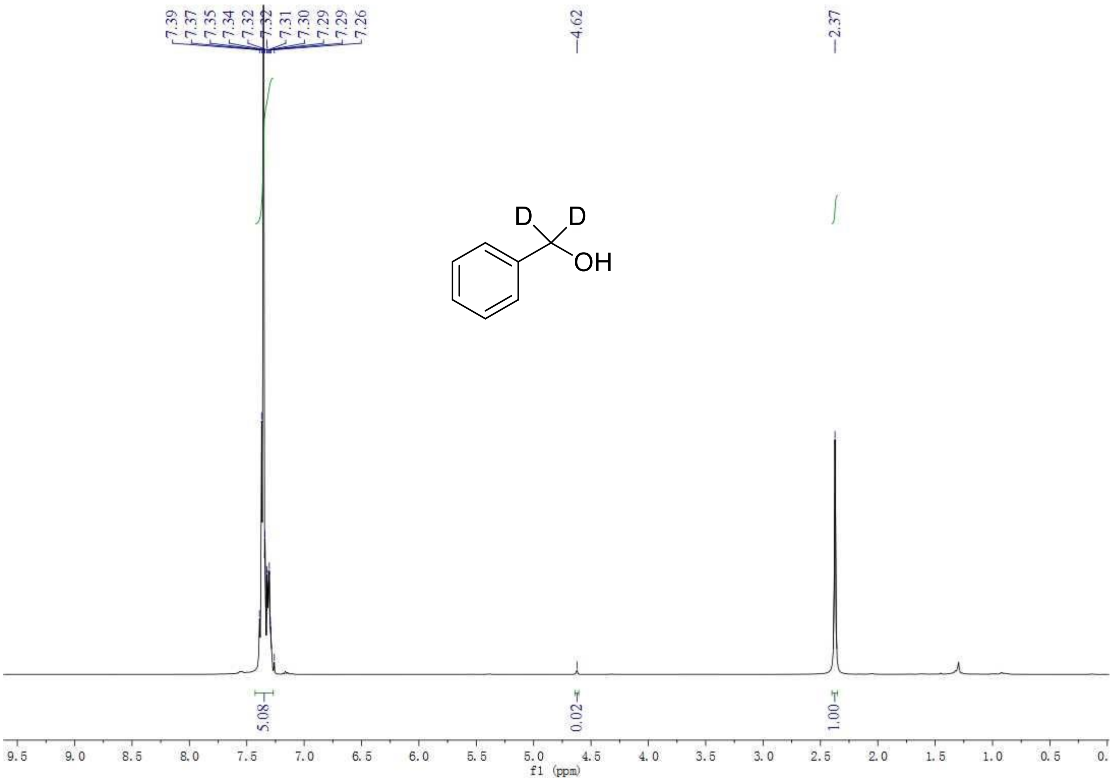

A mixture of $PhCH_2OH$ (0.25 mmol), $PhCD_2OH$ (0.25 mmol) and acetone (1.0 mL) was added to a 10 mL quartz tube with an air balloon at room temperature under the irradiation of 10 W LED lamps (367 – 370 nm) for 4 h. the residual starting material (mixture of phenylmethanol and α-d2-phenylmethanol) was recovered by column chromatography on silica gel (200-300 mesh), which was characterized by ^{1}H NMR spectroscopy. Peak areas at 7.39 - 7.29 ppm and 4.64 ppm were compared to give the ratio (0.45: 0.55) of phenylmethanol to α-d2-phenylmethanol in residual material.

$\boldsymbol{k_H / k_D}$ was calculated using the following expression:

$$\boldsymbol{K_H/K_D} = \frac{M / 2 - 0.45m}{M / 2 - 0.55m}$$

M, m represent the amount of phenylmethanol and *α-d2*-phenylmethanol in reaction staring material and residual material, espectively. Here, M = 0.5, m = 0.42, which corresponds to $\boldsymbol{k_H/k_D}$ = 3.2.

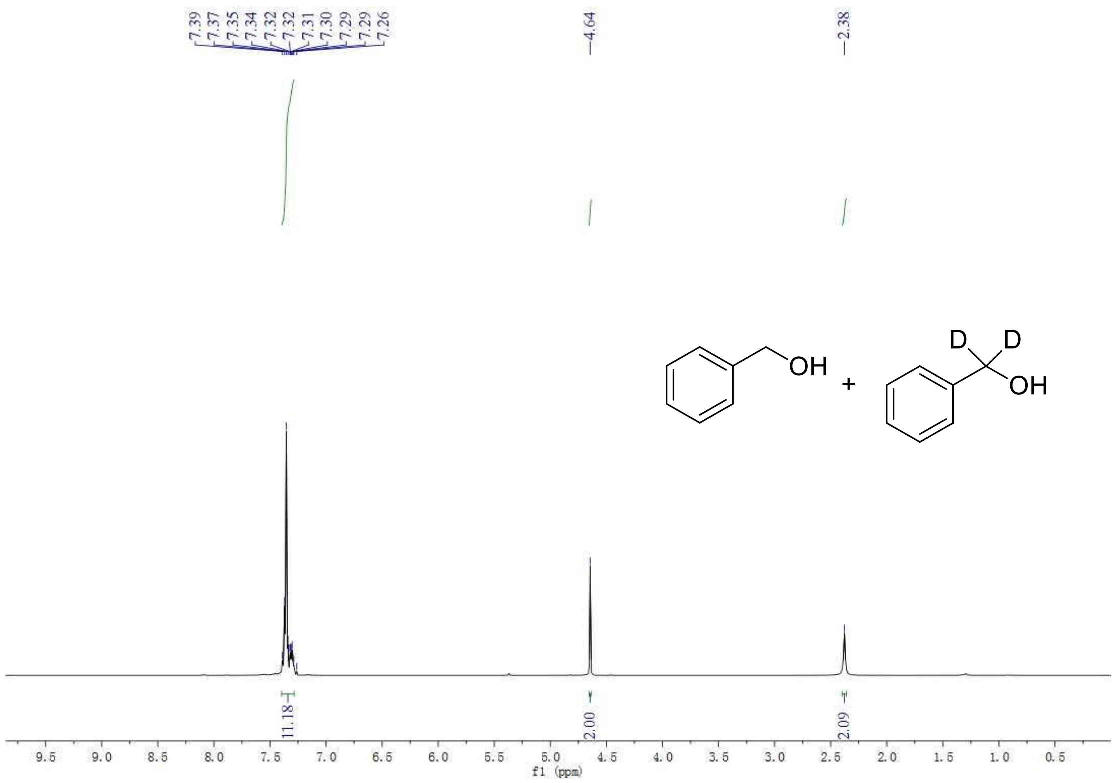

(f) Catalytic experiment:

A mixture of phenylmethanol **1a** (0.5 mmol), benzaldehyde (0.05 mmol) and acetone (1.0 mL) was added to a 10 mL quartz tube with an air balloon at room temperature under the irradiation of 10 W LED lamps (367 – 370 nm) for 16 h. GC-MS analysis of the reaction mixture showed that 99% yield of **3a** was obtained.

(g) UV/Vis Absorption Experiment

(1) The UV-vis absorption spectrum of phenylmethanol (0.5 mmol) in acetone (1.0 mL) were recorded in 1 cm path quartz cuvette by using a SHIMADZU UV-2600 UV-visible spectrophotometer. The obtained bands in UV/vis absorption spectra were shown in Figure S2.

(2) The UV-vis absorption spectrum of benzaldehyde (0.5 mmol) in acetone (1.0 mL) were recorded in 1 cm path quartz cuvette by using a SHIMADZU UV-2600 UV-visible spectrophotometer. The obtained bands in UV/vis absorption spectra were shown in Figure S2.

(3) The UV-vis absorption spectrum of benzoic acid (0.5 mmol) in acetone (1.0 mL) were recorded in 1 cm path quartz cuvette by using a SHIMADZU UV-2600 UV-visible spectrophotometer. The obtained bands in UV/vis absorption spectra were shown in Figure S2.

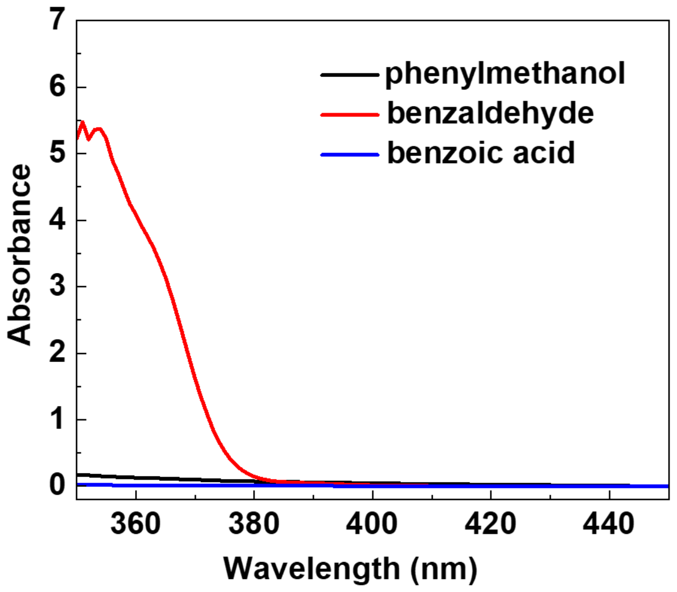

Figure S2. UV-Vis Spectroscopic

(h) Determination of singlet oxygen in the reaction

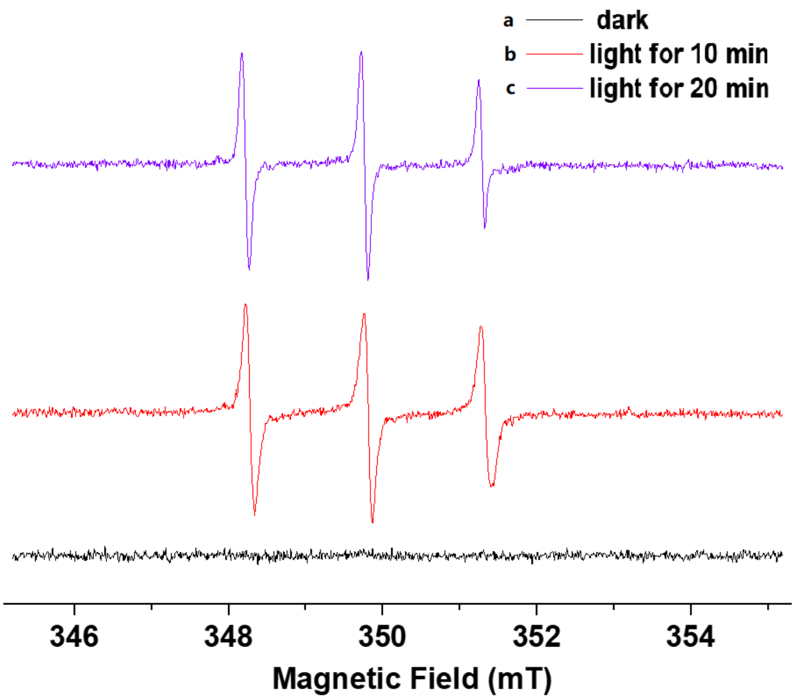

Figure S3. The X-band electron paramagnetic resonance (EPR) spectra of the singlet oxygen captured by TMPD. The g-value (g = 2.0065) is derived from the spectrum. (a) solution of TMPD and phenylmethanol (**1a**) in air-saturated acetone without

light irradiation; (b) solution of TMPD and phenylmethanol (**1a**) in air-saturated acetone with light irradiation for 10 min; (c) solution of TMPD and phenylmethanol (**1a**) in air-saturated acetone with light irradiation for 20 min.

In order to determine the active species of oxygen involved in the present reaction, 2,2,6,6-tetramethylpiperidin-4-one (TMPD) was employed to capture $^1O_2$. As shown in Figure 3a, no signal was detected when the acetone solution of TMPD and phenylmethanol (**1a**) without irradiation . However, when the same solution was irradiated with 367 – 370 nm wavelength light, a caracteristic signal of $^1O_2$ adduct with TMPD was observed (Figure S**3b** and S**3c**).

(i) Determination of peroxy radical in the reaction

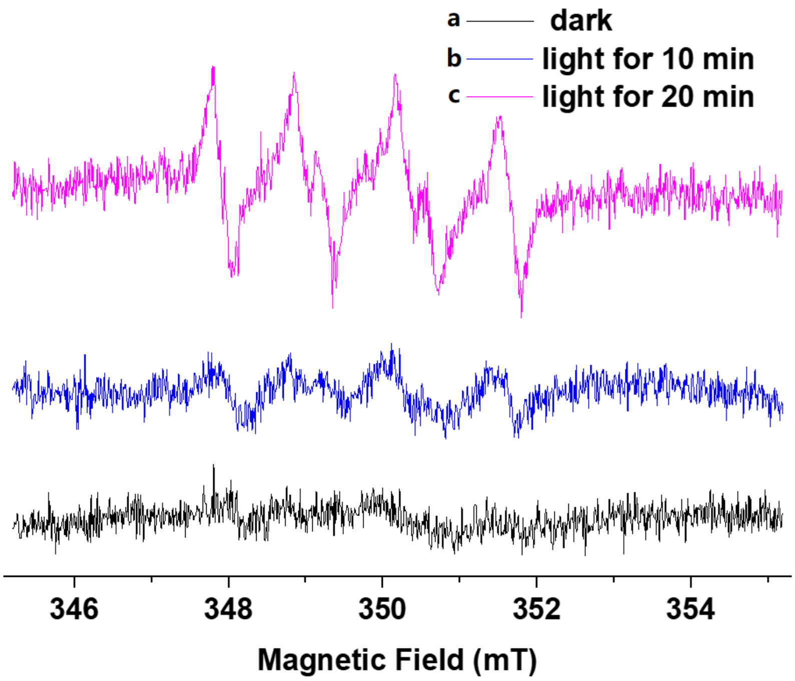

Figure S4. The X-band electron paramagnetic resonance (EPR) spectra of the peroxy radical captured by DMPO. (a) solution of DMPO and phenylmethanol (**1a**) in air-saturated acetone without light irradiation; (b) solution of DMPO and phenylmethanol (**1a**) in air-saturated acetone with light irradiation for 10 min; (c) solution of DMPO and phenylmethanol (**1a**) in air-saturated acetone with light irradiation for 20 min.

In order to determine the active species of oxygen involved in the present reaction, 5,5-dimethyl-1-pyrroline *N*-oxide (DMPO) was employed to capture peroxy radical. As shown in Figure S**4a**, no signal was detected when the acetone solution of DMPO and phenylmethanol (**1a**) without irradiation. However, when the solution was irradiated with 367 – 370 nm

wavelength light, a caracteristic signal of peroxy radical adduct with DMPO was observed for 10 min and 20 min (Figure S**4b** and S**4c**).

S3. Comparison of methodology

Table S1 Comparison of reaction conditions and yields between this methodology and other schemes reported in the literature

Entry	Substrate	Reaction condition	Yield (%)	Ref.
1	R-OH	$Fe(NO_3)_3$•$9H_2O$/TEMPO/KCl，DCE，O_2，r.t.	55-100	[1]
2	R-OH	Base, [$NiCl_2$(L3)], 150 ºC, Ar	40-90	[2]
3	R-OH	N-hydroxyphthalimide/tetramethylammonium bromide/CZJ-11, CH_3CN, O_2, 50 ºC	52-99	[3]
4	R-CHO	4-Acetamido-TEMPO, aqueous carbonate buffer (0.1 M $NaHCO_3$/0.1 M Na_2CO_3, pH 9.8−10.1), electrolysis at 0.7 V vs Ag/AgCl.	83-99	[4]
5	R-OH	30-$W_{18}O_{49}$/HU-CNS, H_2O	99	[5]
6	R-OH	MOF-TEMPO, tert-butyl nitrite, CH_3CN, O_2, r.t.	50-99	[6]
7	R-CH=CH_2	1,2-Dibutoxyethane, O_2, 110 ºC	73-97	[7]
8	R-CHO	Sunlight, O_2, acetone, r.t.	15-99	[8]
9	R-OH	Acetone, 367-370 nm 10 W LED, air, r.t.	71-95	This work

S4. ^{1}H and ^{13}C NMR spectra of products

^{1}H NMR spectrum of benzoic acid (3a, 400 MHz, DMSO-*d6*)

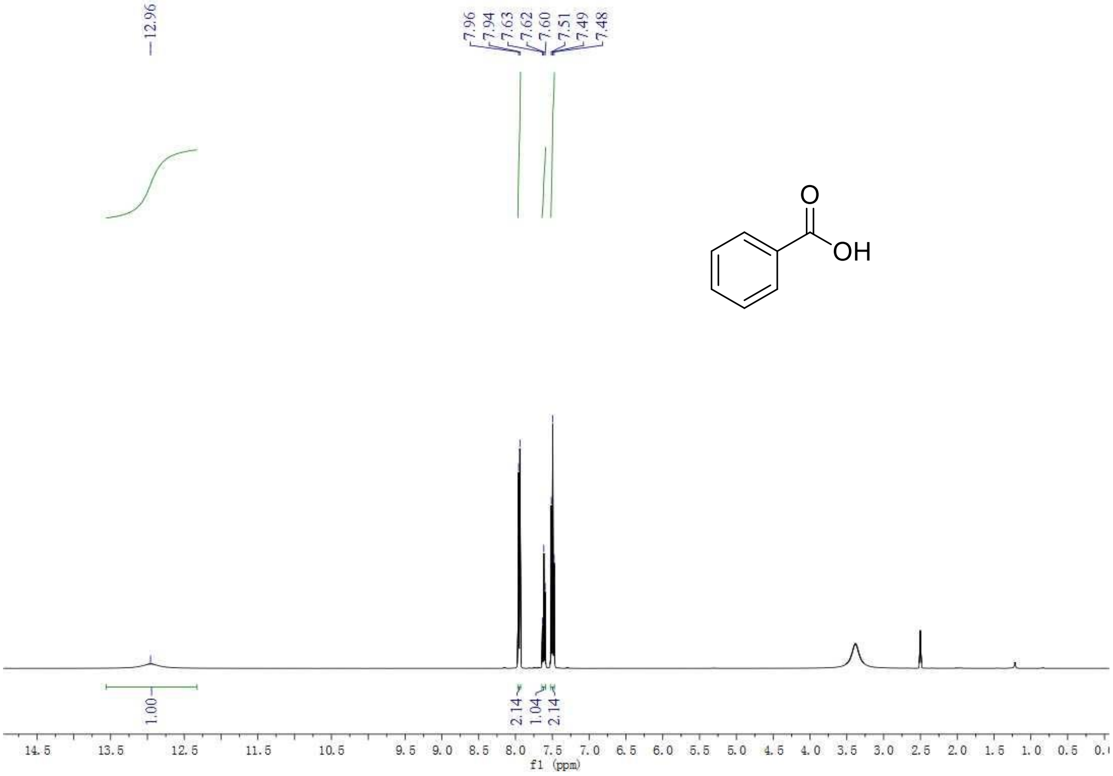

$^{13}C\{1H\}$ NMR spectrum of benzoic acid (3a, 101 MHz, DMSO-*d6*)

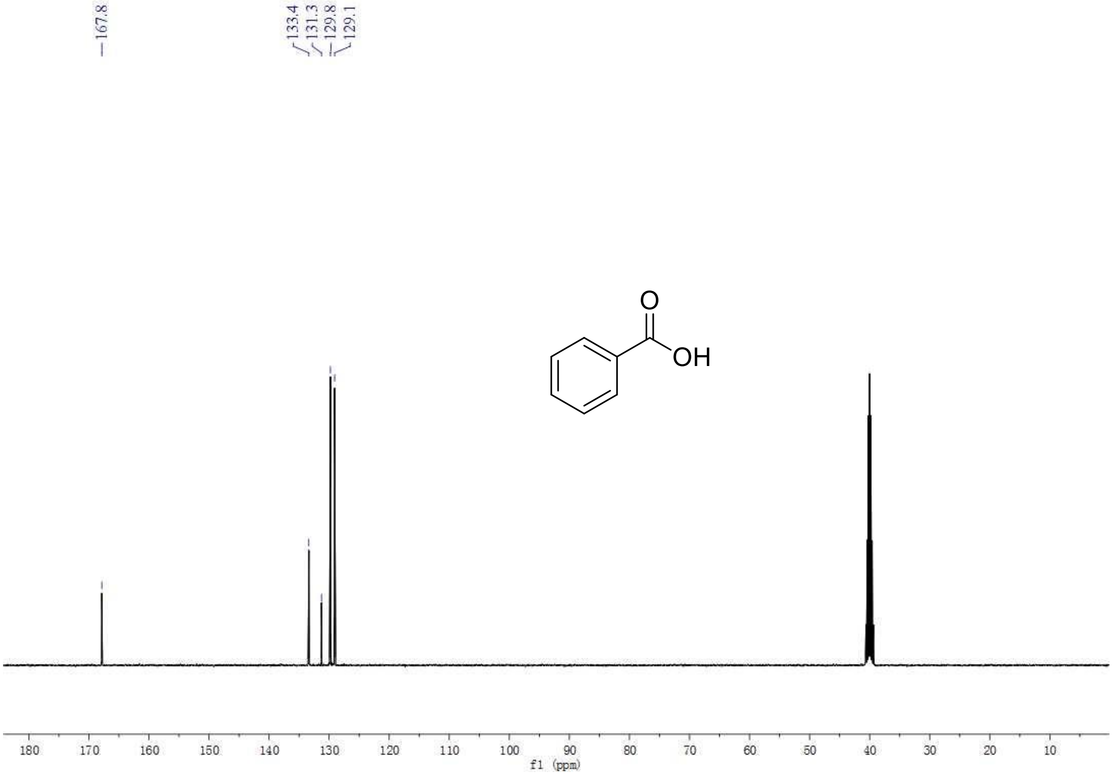

^{1}H NMR spectrum of [1,1'-biphenyl]-4-carboxylic acid (3b, 400 MHz, DMSO-*d6*)

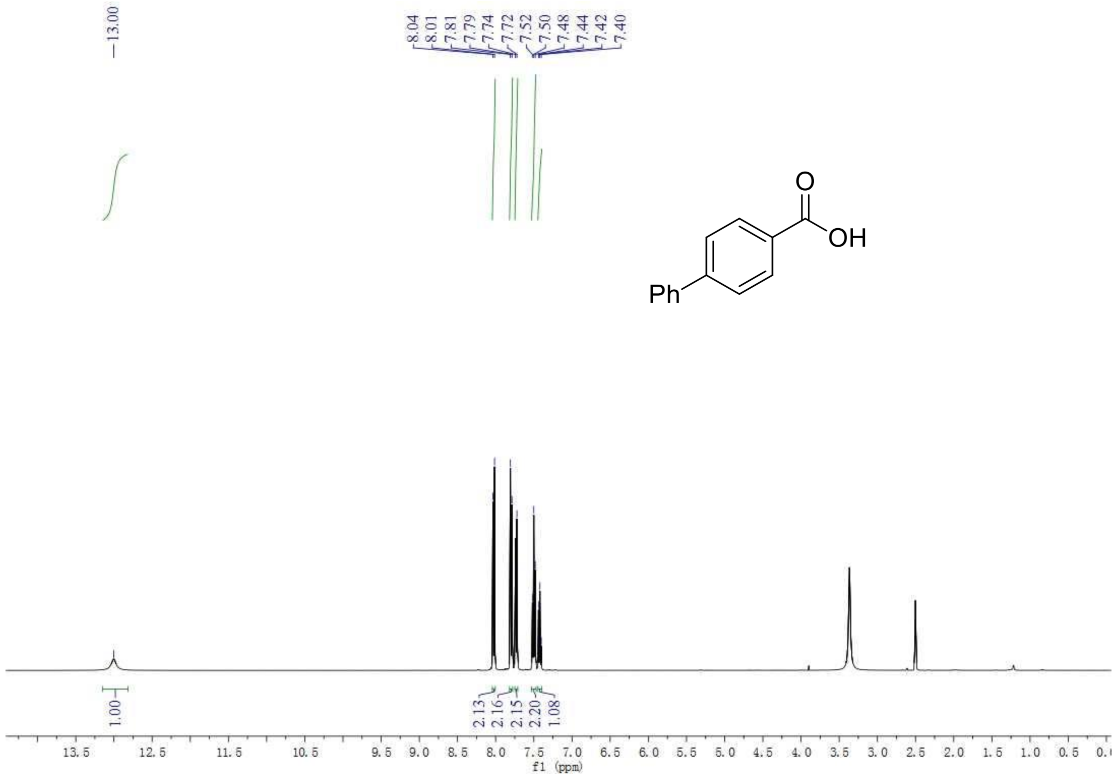

^{13}C{1H} NMR spectrum of [1,1'-biphenyl]-4-carboxylic acid (3b, 101 MHz, DMSO-*d6*)

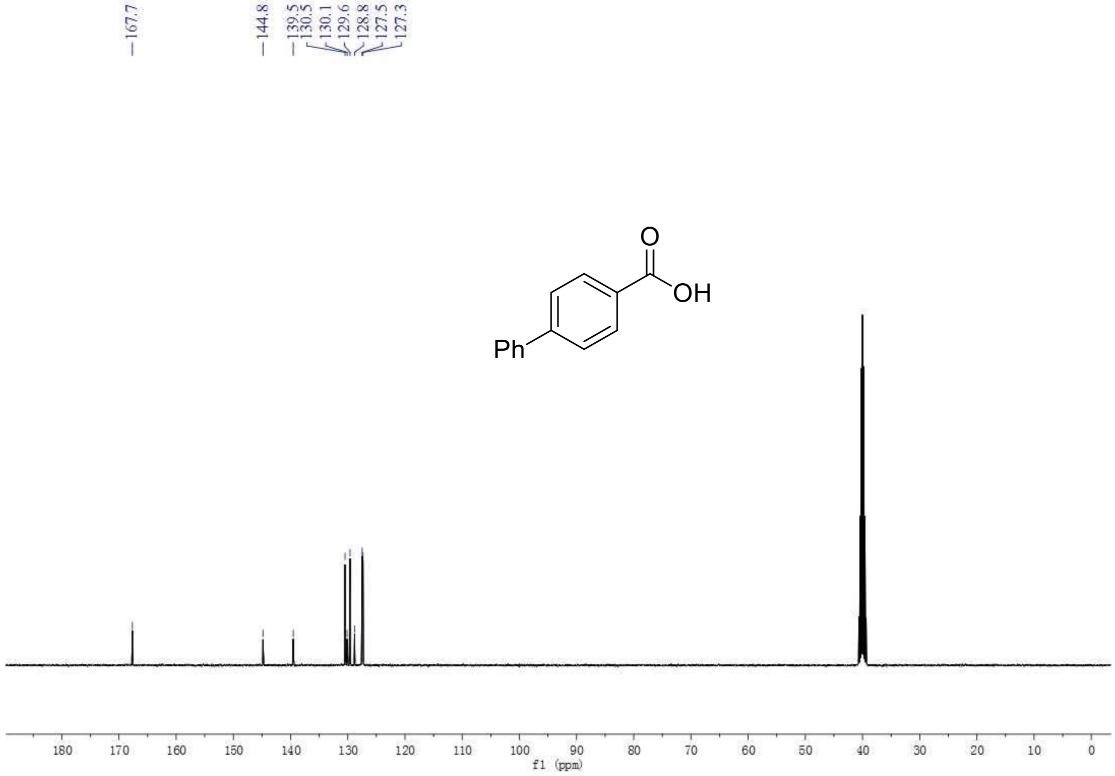

^{1}H NMR spectrum of 4-methylbenzoic acid (3c, 400 MHz, DMSO-*d6*)

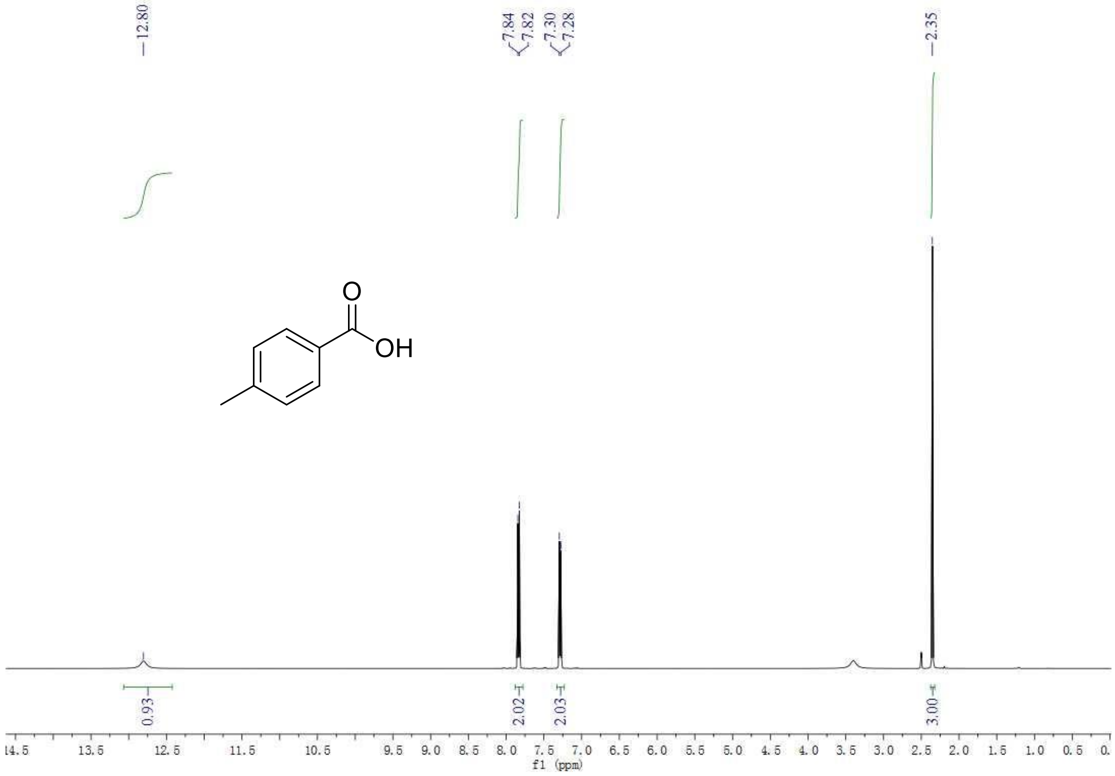

^{13}C{1H} NMR spectrum of 4-methylbenzoic acid (3c, 101 MHz, DMSO-*d6*)

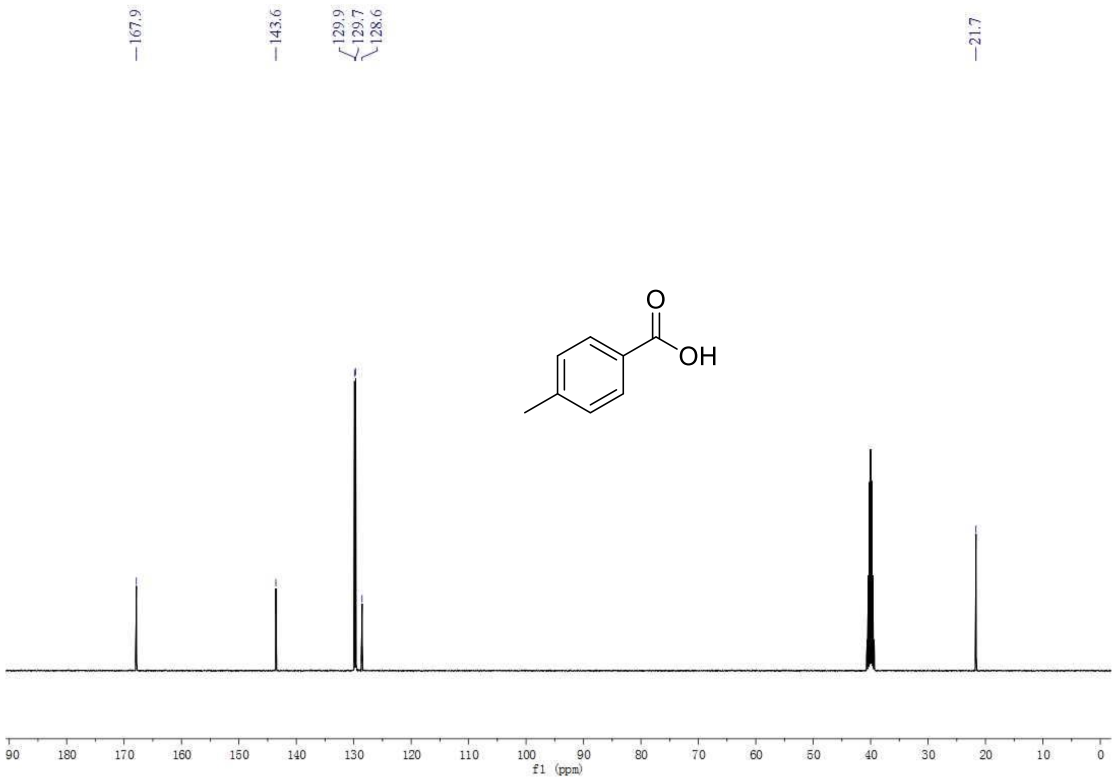

^{1}H NMR spectrum of 4-isopropylbenzoic acid (3d, 400 MHz, DMSO-*d6*)

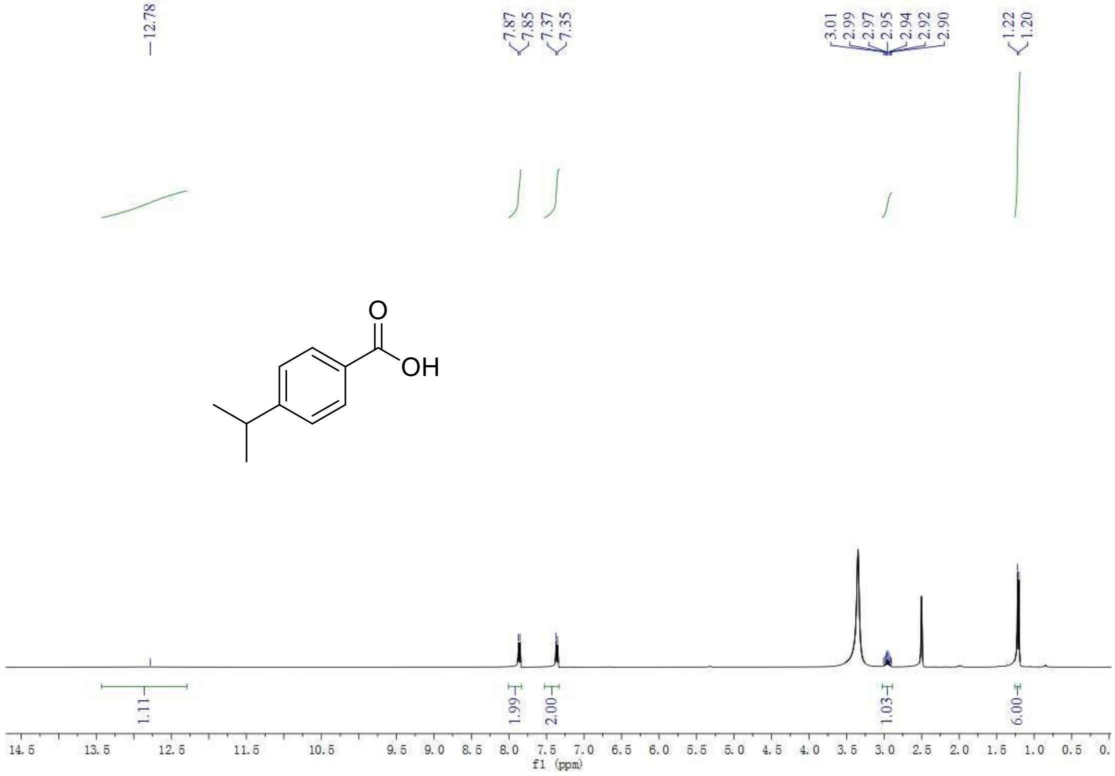

^{13}C{1H} NMR spectrum of 4-isopropylbenzoic acid (3d, 101 MHz, DMSO-*d6*)

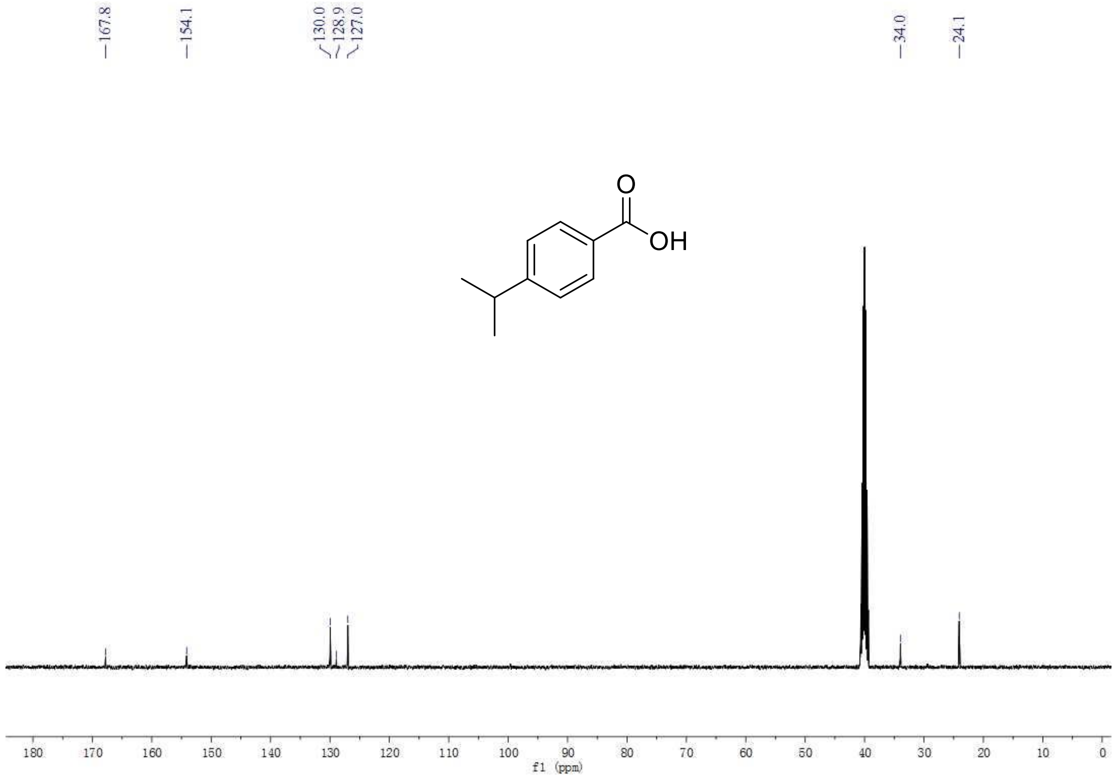

^{1}H NMR spectrum of 4-(*tert*-butyl)benzoic acid (3e, 400 MHz, DMSO-*d6*)

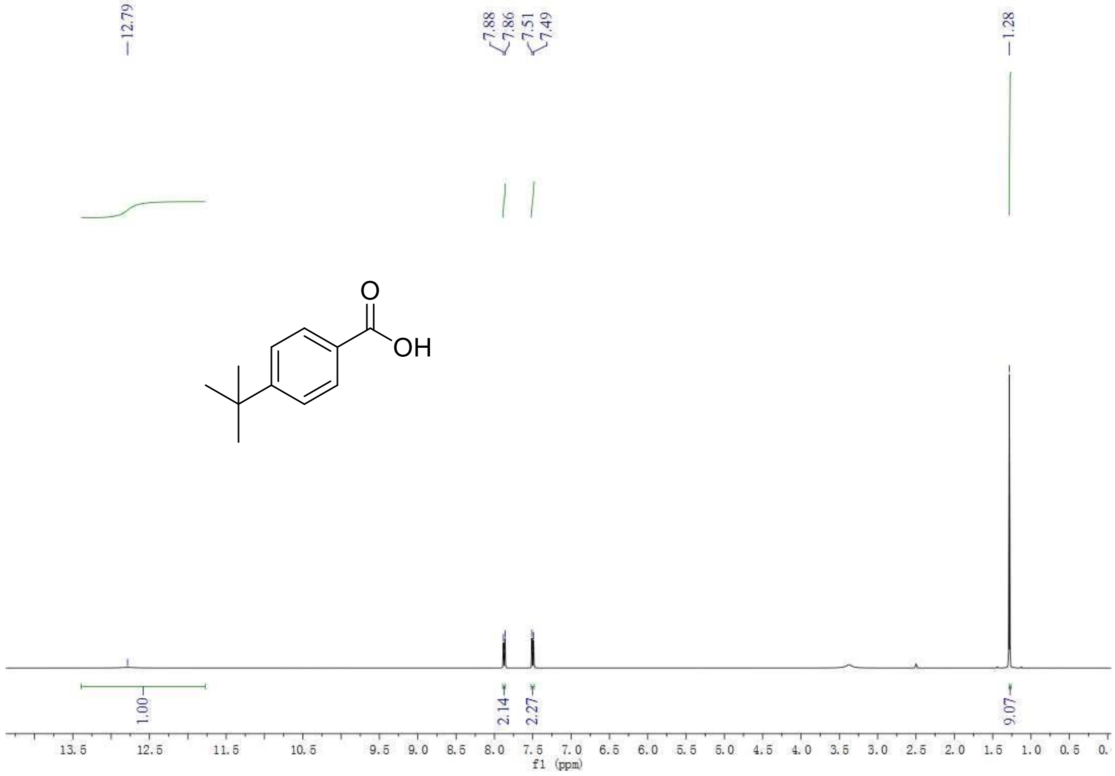

^{13}C{1H} NMR spectrum of 4-(*tert*-butyl)benzoic acid (3e, 101 MHz, DMSO-*d6*)

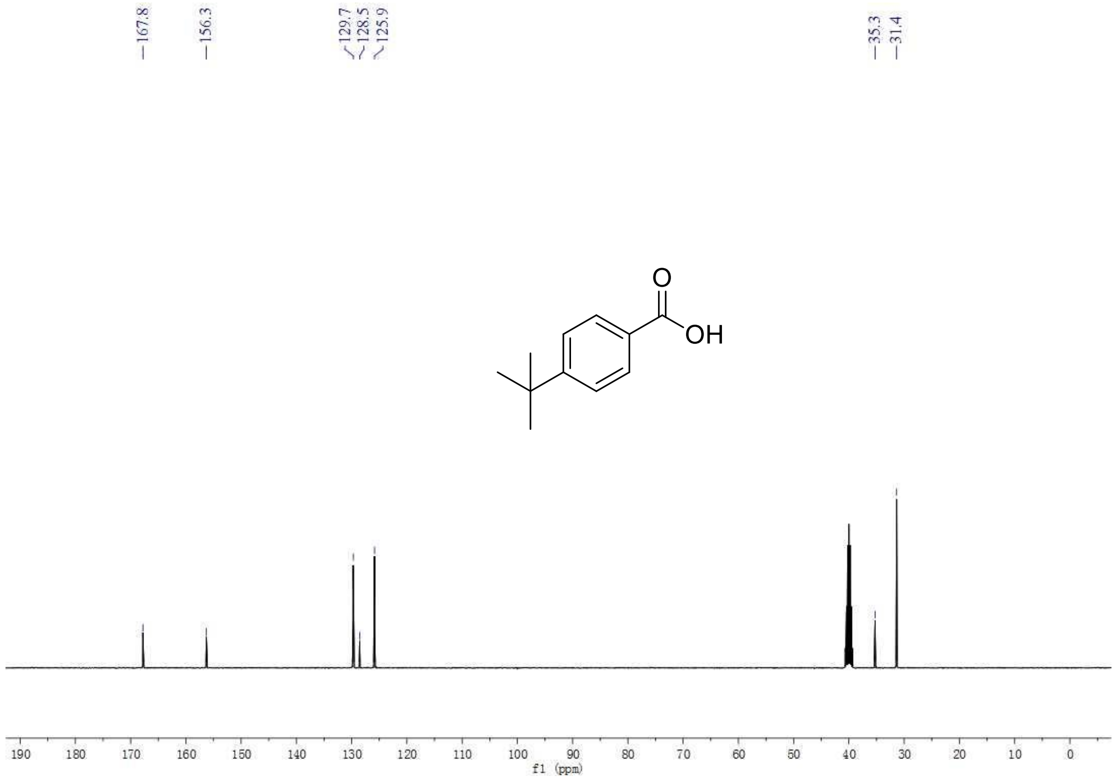

^{1}H NMR spectrum of 4-(trifluoromethoxy)benzoic acid (3f, 400 MHz, DMSO-*d6*)

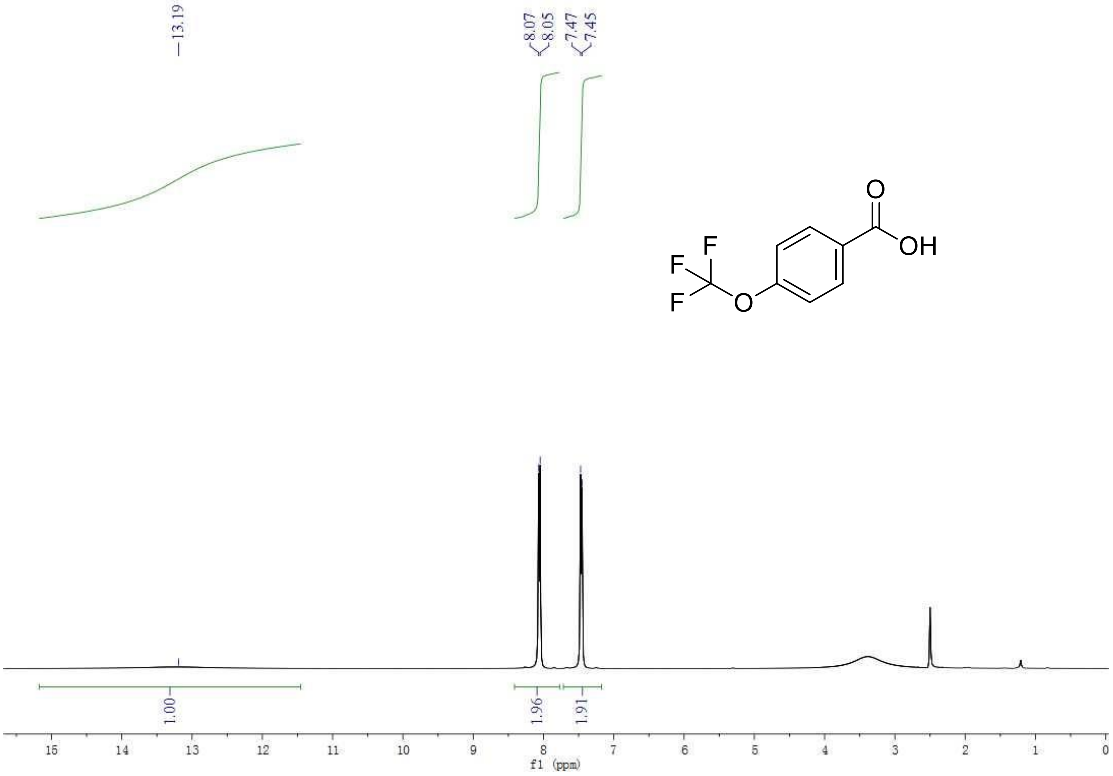

^{13}C{1H} NMR spectrum of 4-(trifluoromethoxy)benzoic acid (3f, 101 MHz, DMSO-*d6*)

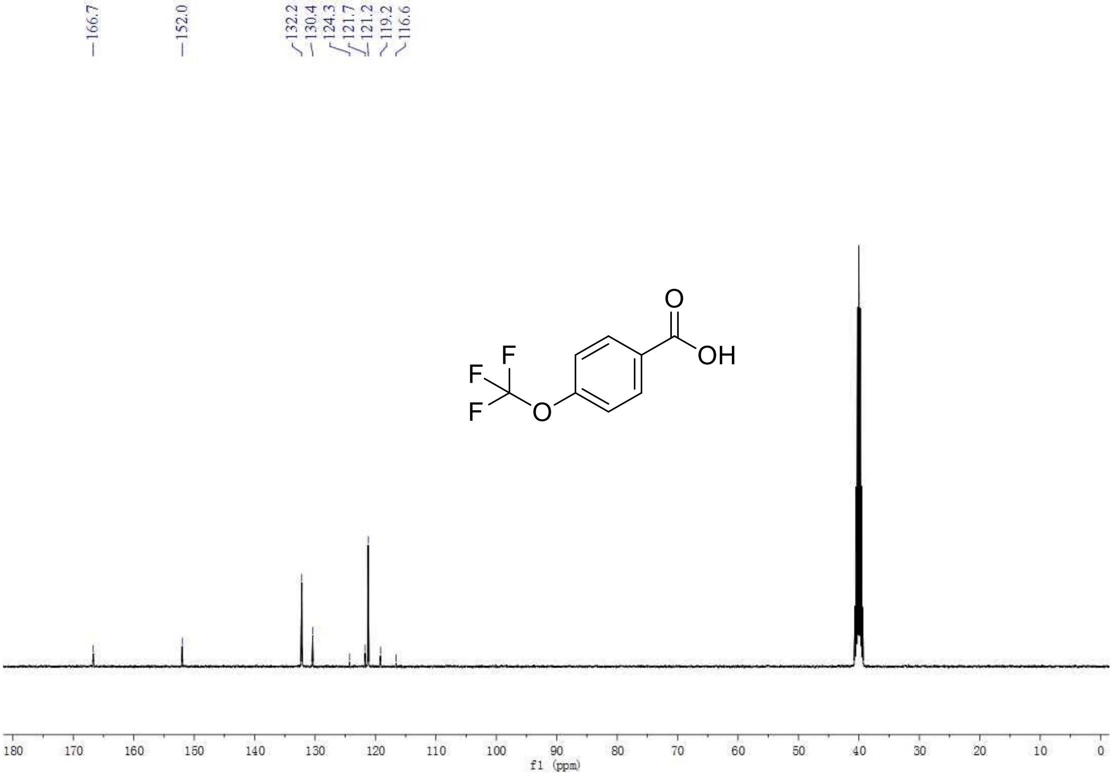

^{19}F NMR spectrum of 4-(trifluoromethoxy)benzoic acid (3f, 376 MHz, DMSO-*d6*)

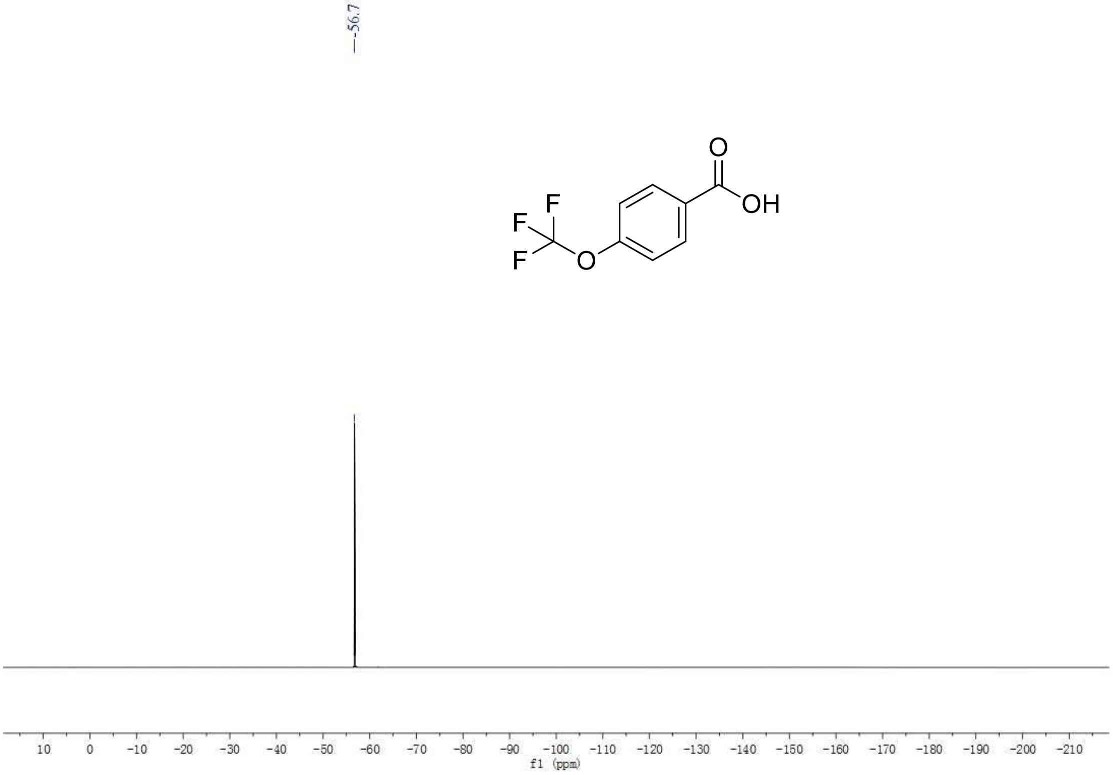

^{1}H NMR spectrum of 4-fluorobenzoic acid (3g, 400 MHz, DMSO-*d6*)

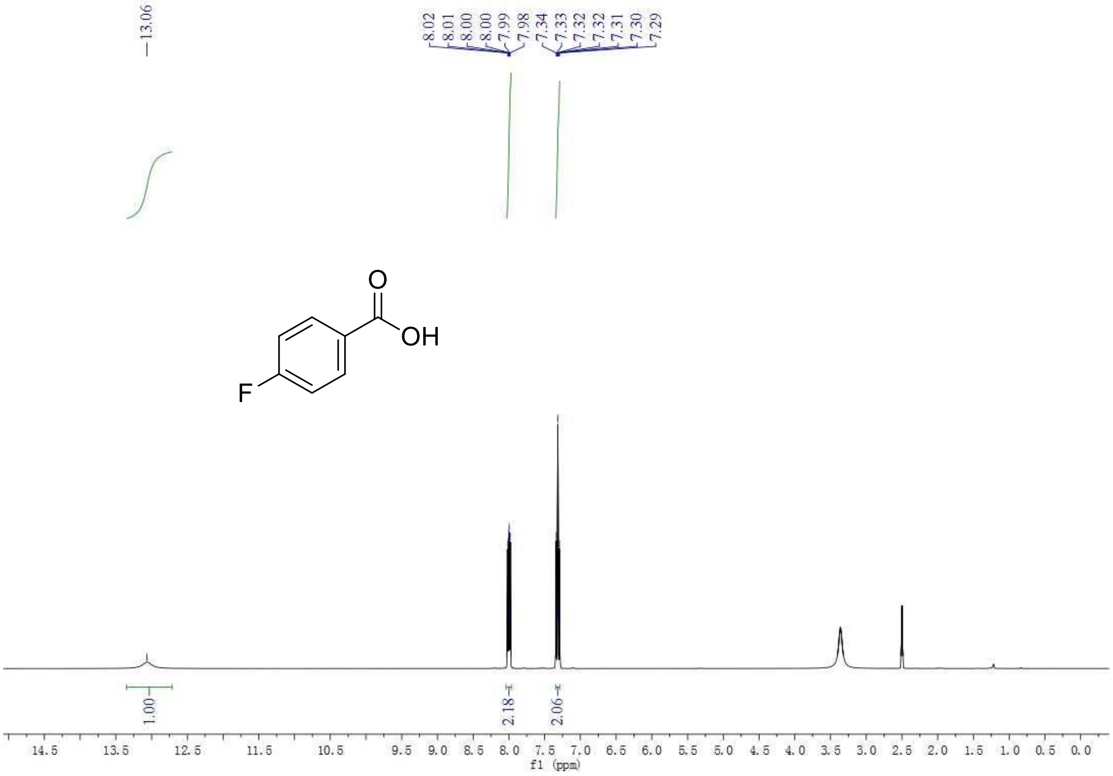

^{13}C{1H} NMR spectrum of 4-fluorobenzoic acid (3g, 101 MHz, DMSO-*d6*)

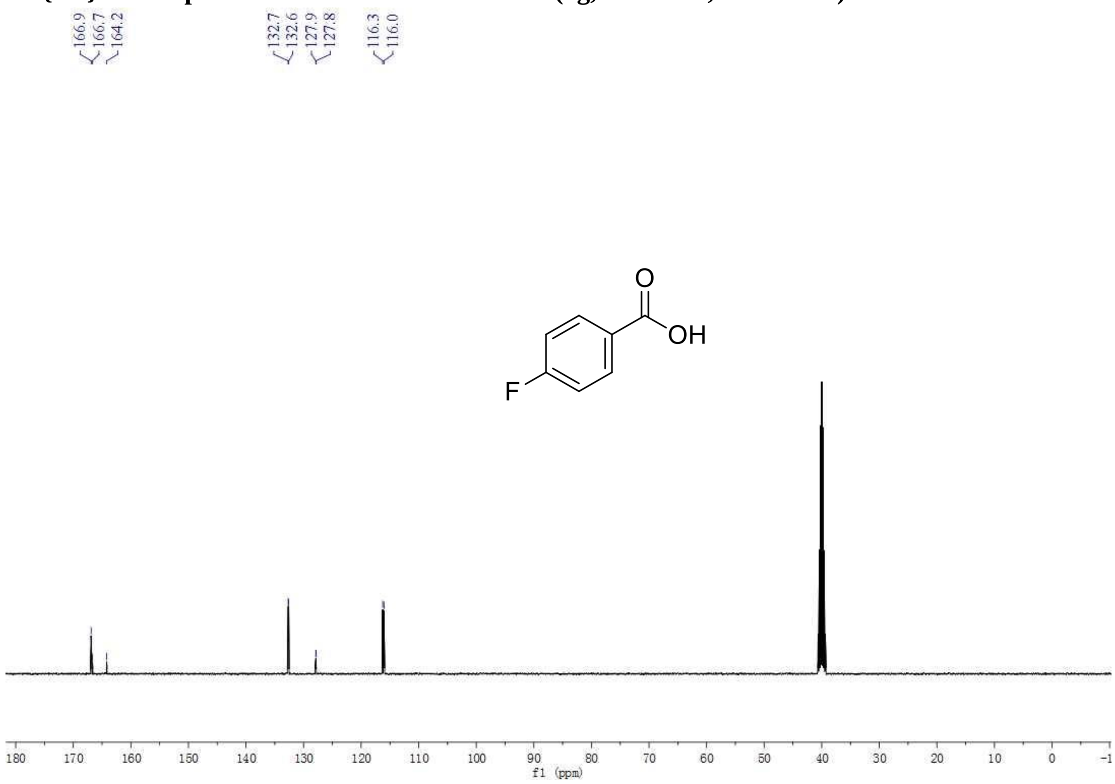

^{19}F NMR spectrum of 4-fluorobenzoic acid (3g, 376 MHz, DMSO-*d6*)

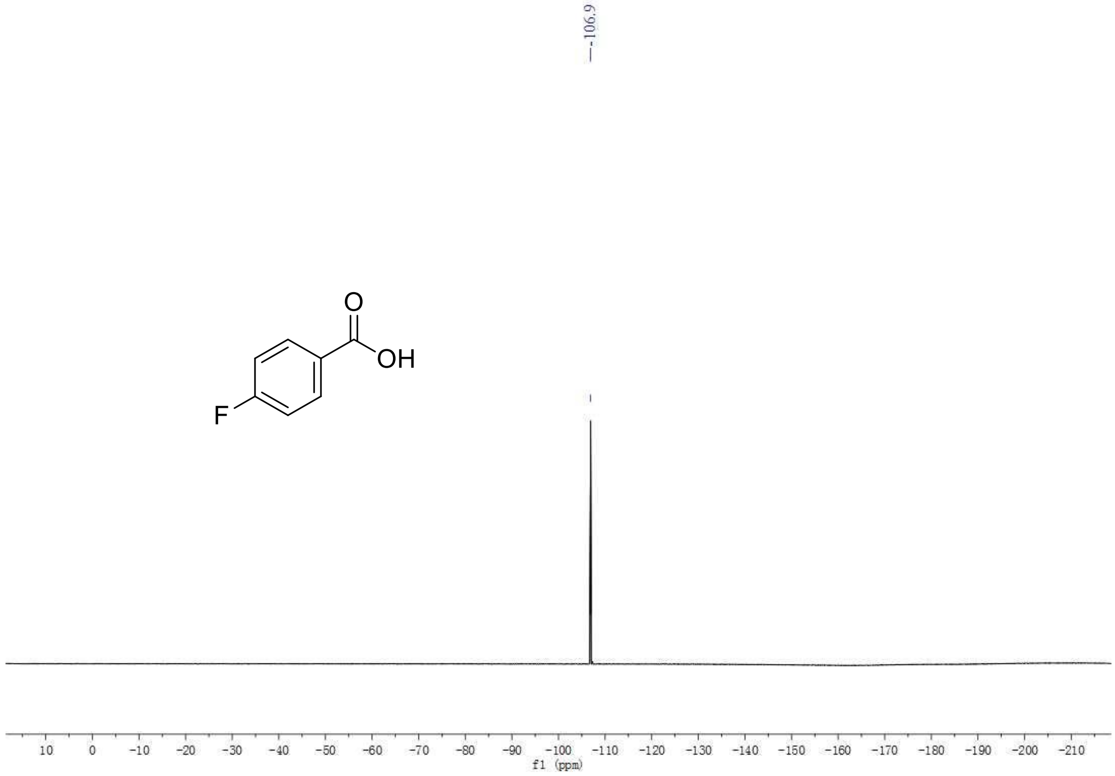

^{1}H NMR spectrum of 4-chlorobenzoic acid (3h, 400 MHz, DMSO-*d6*)

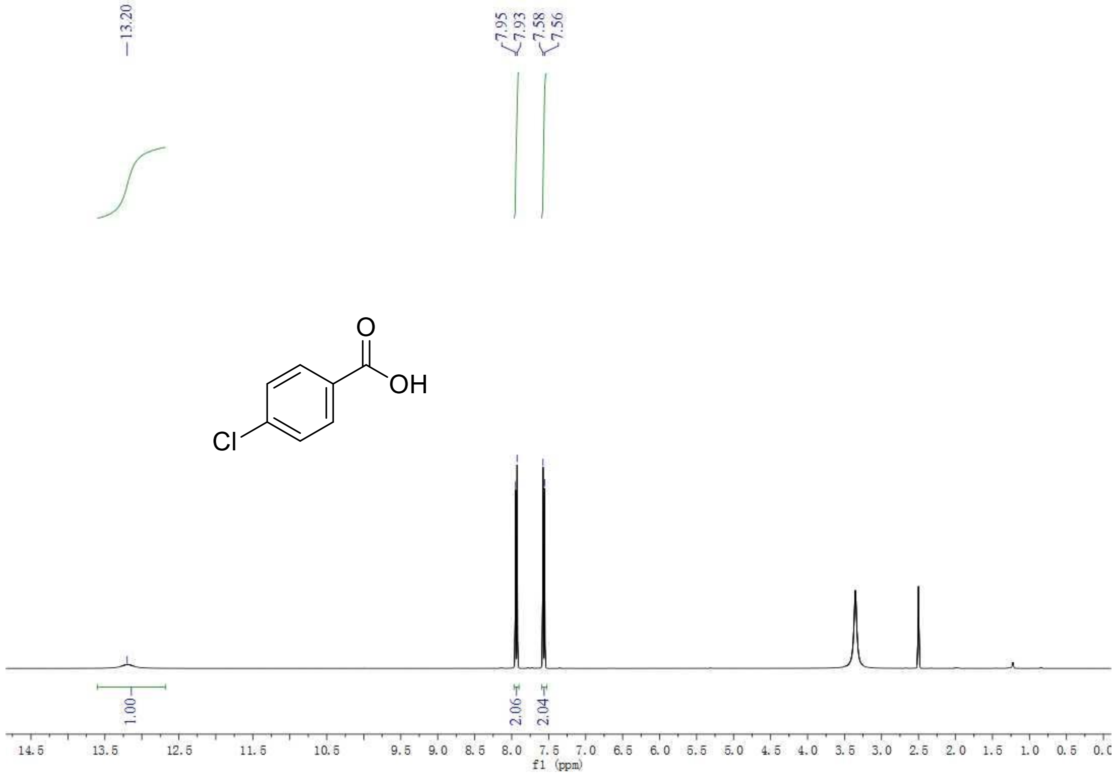

^{13}C{1H} NMR spectrum of 4-chlorobenzoic acid (3h, 101 MHz, DMSO-*d6*)

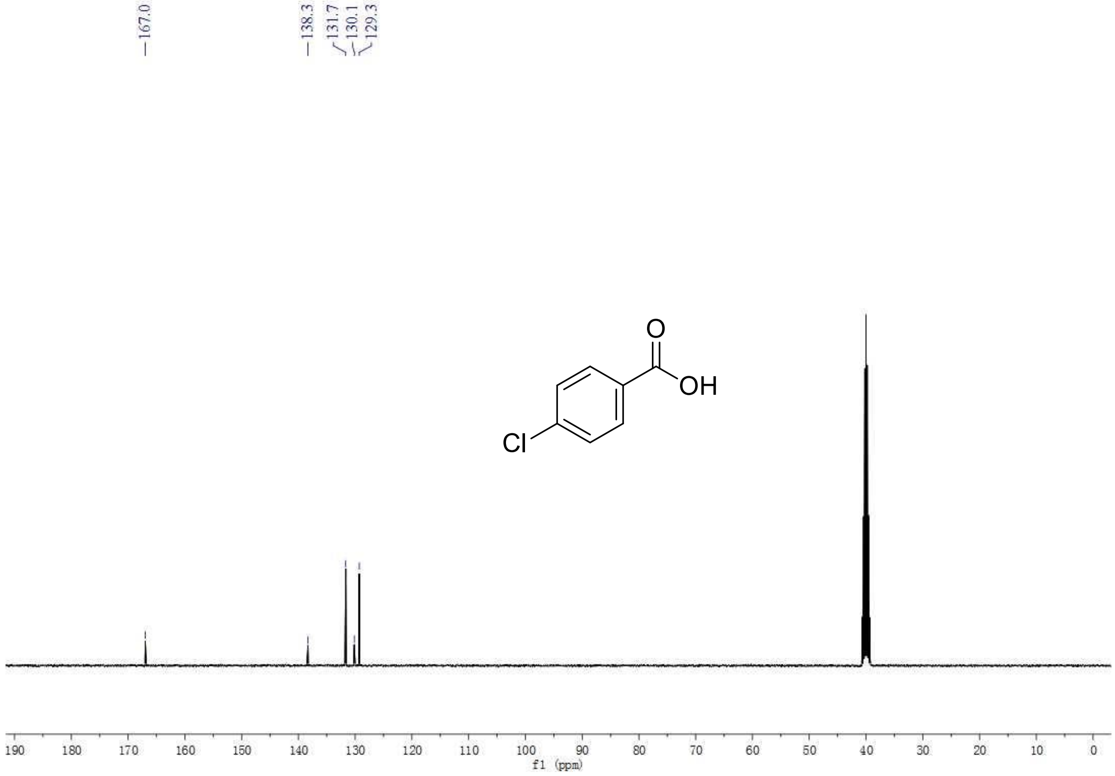

^{1}H NMR spectrum of 4-bromobenzoic acid (3i, 400 MHz, DMSO-*d6*)

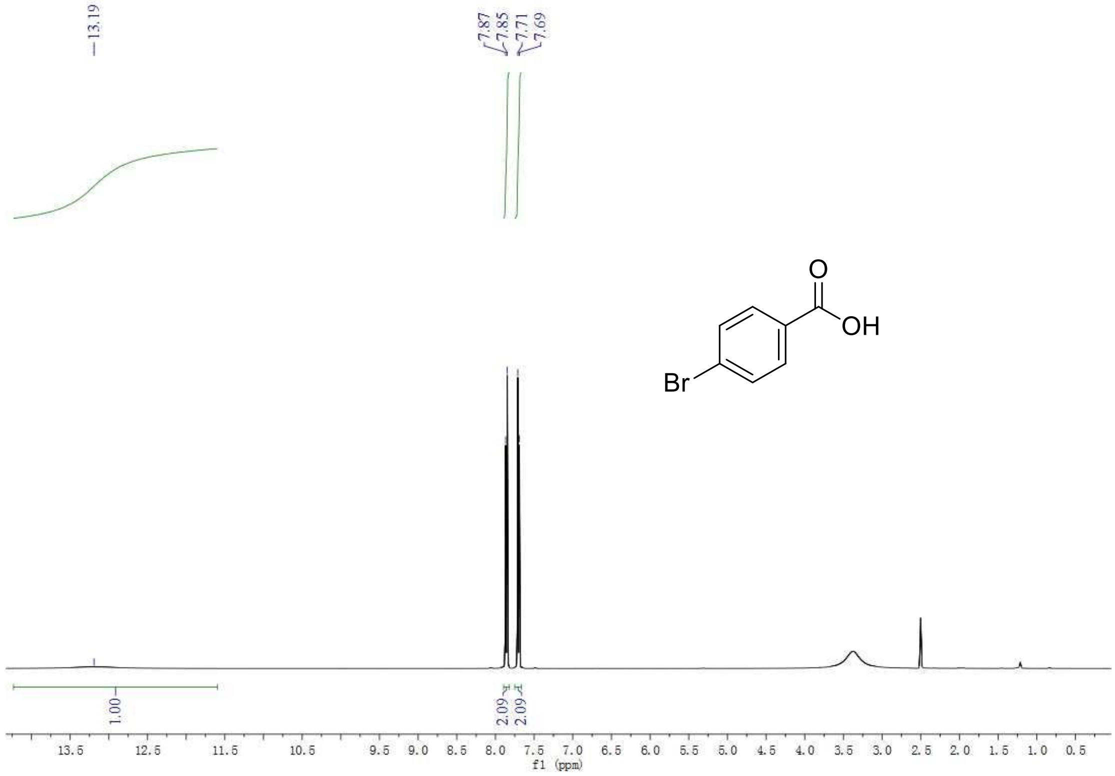

^{13}C{1H} NMR spectrum of 4-bromobenzoic acid (3i, 101 MHz, DMSO-*d6*)

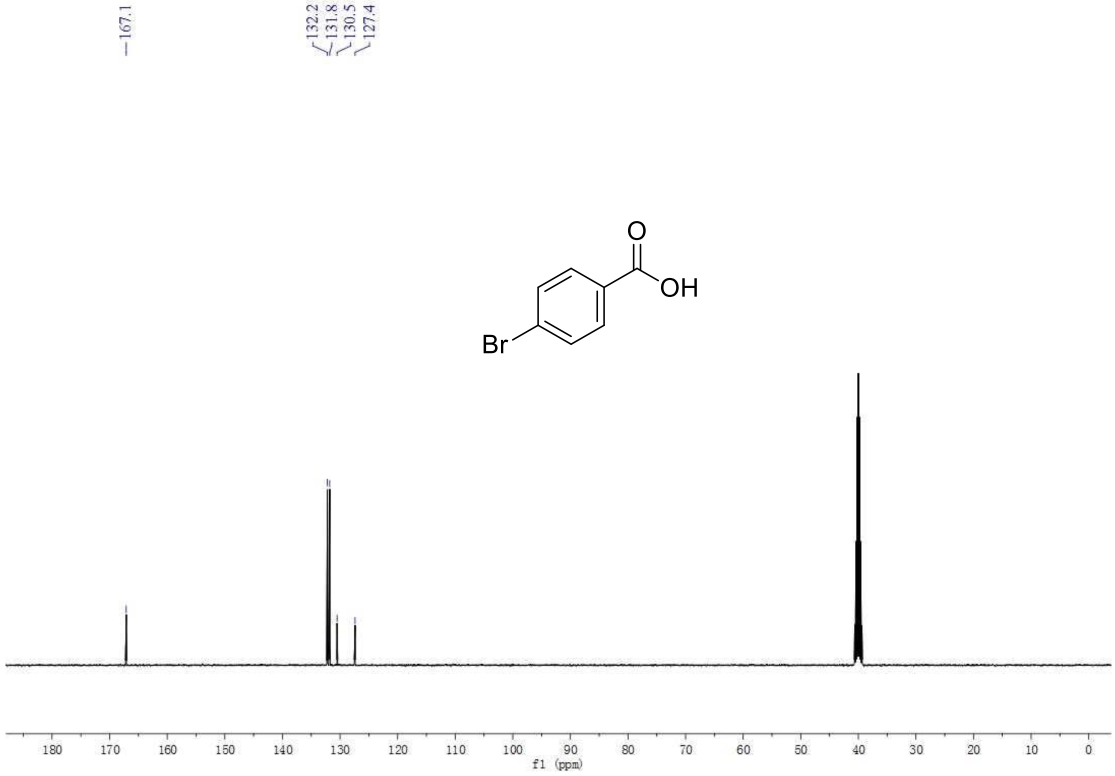

^{1}H NMR spectrum of 4-cyanobenzoic acid (3j, 400 MHz, DMSO-*d6*)

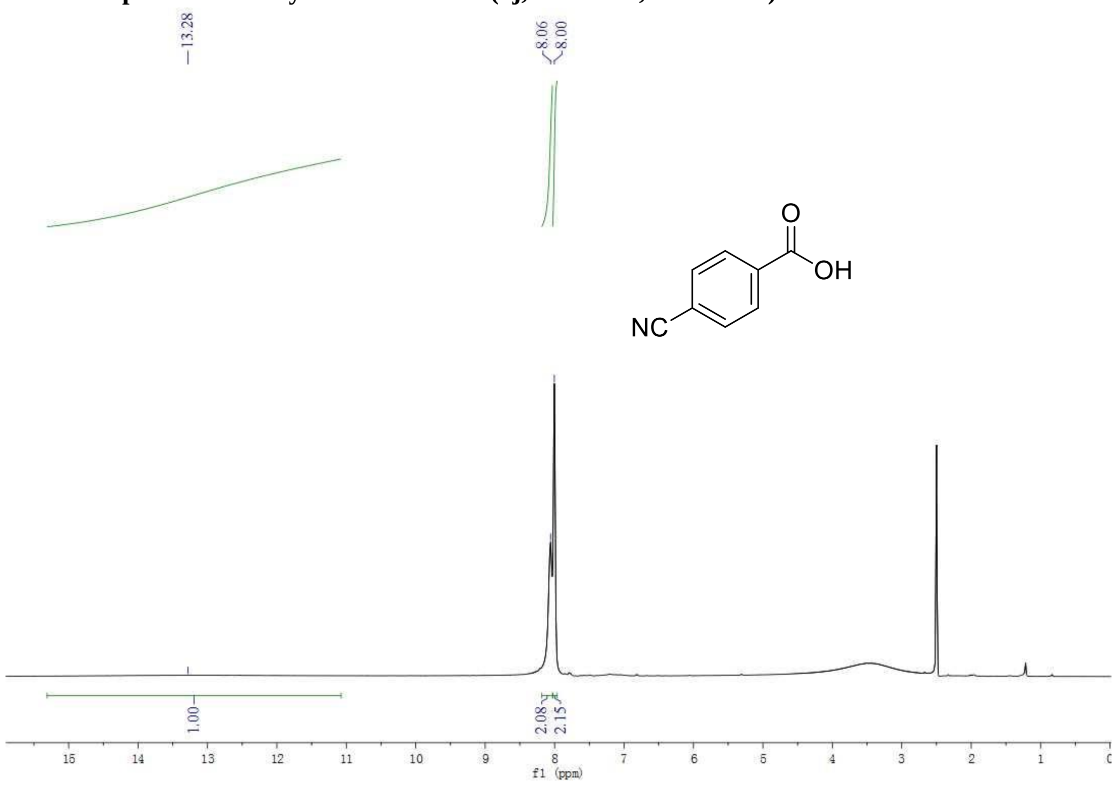

^{13}C{1H} NMR spectrum of 4-cyanobenzoic acid (3j, 101 MHz, DMSO-*d6*)

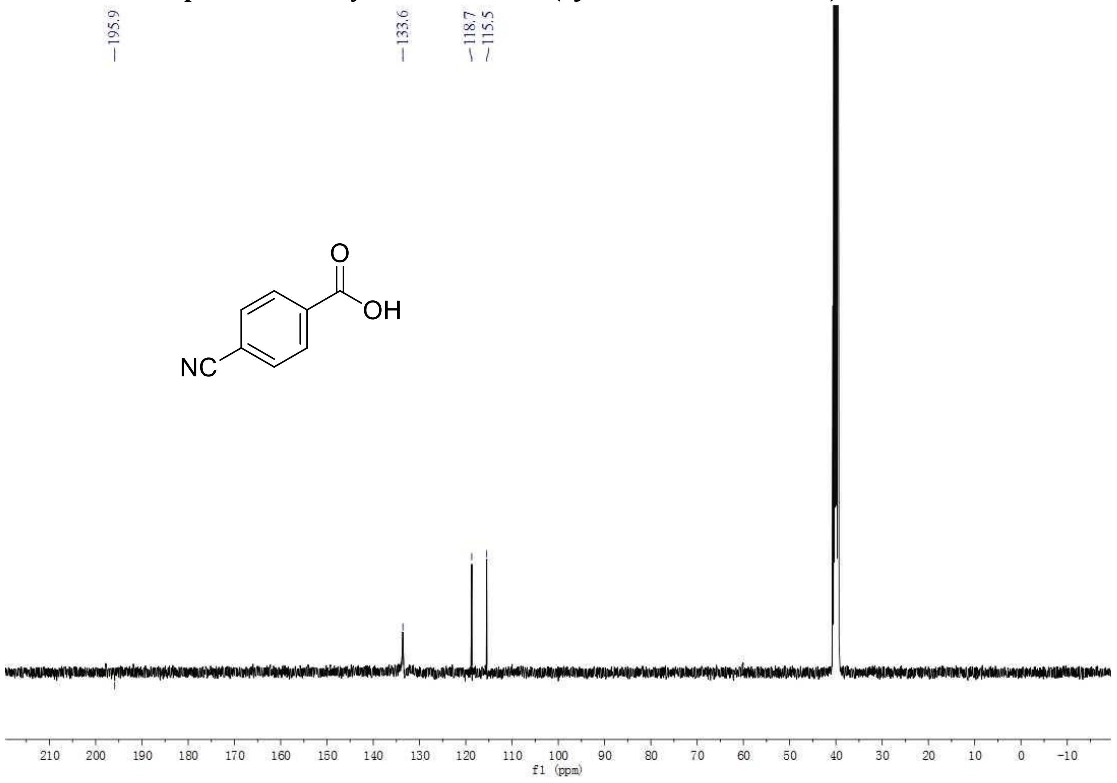

^{1}H NMR spectrum of 4-(methoxycarbonyl)benzoic acid (3k, 400 MHz, DMSO-*d6*)

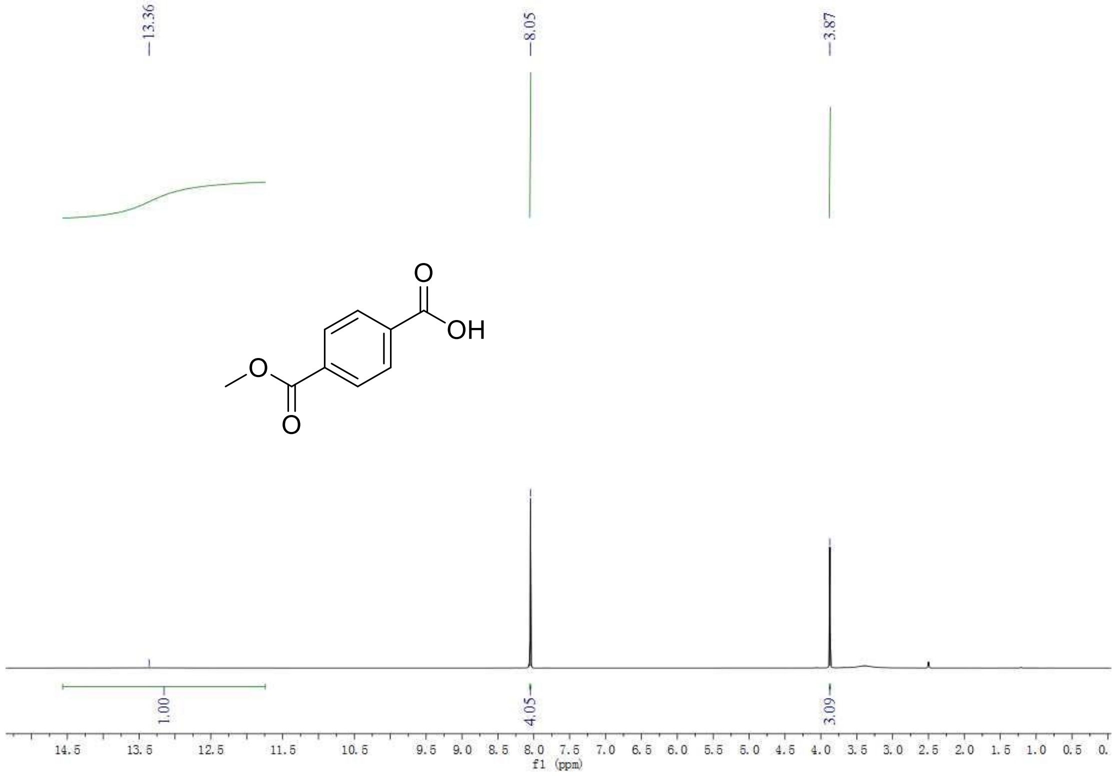

^{13}C{1H} NMR spectrum of 4-(methoxycarbonyl)benzoic acid (3k, 101 MHz, DMSO-*d6*)

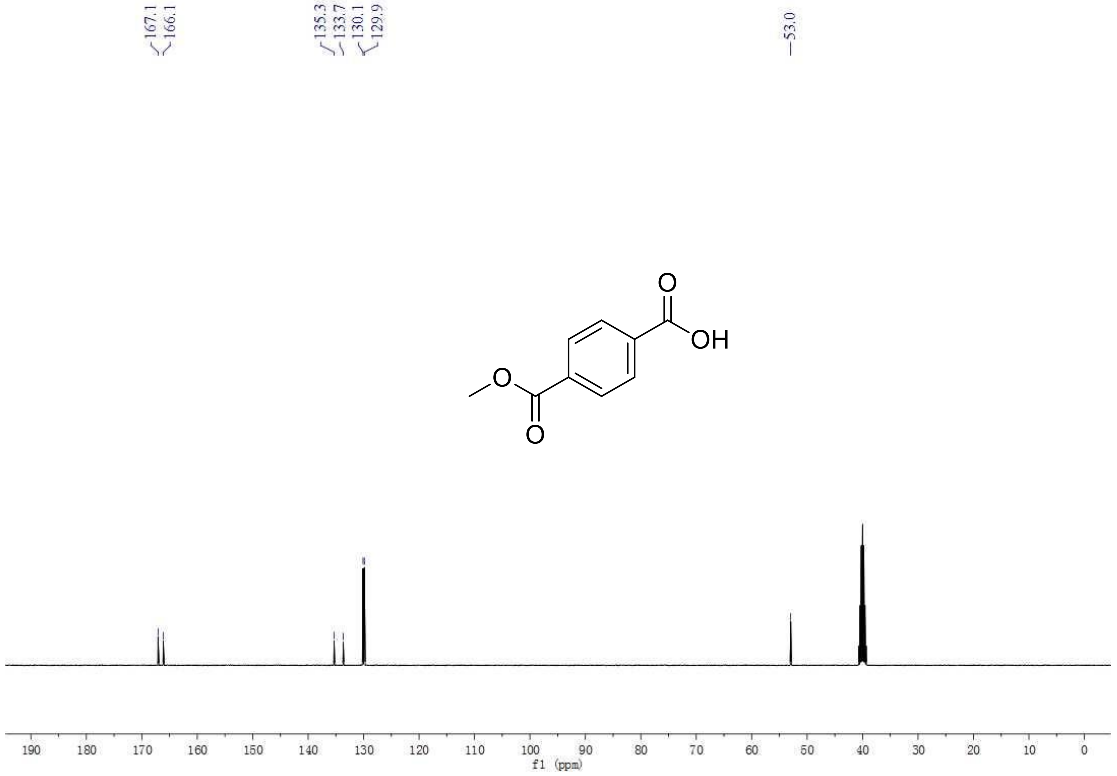

^{1}H NMR spectrum of 2-chlorobenzoic acid (3l, 400 MHz, DMSO-*d6*)

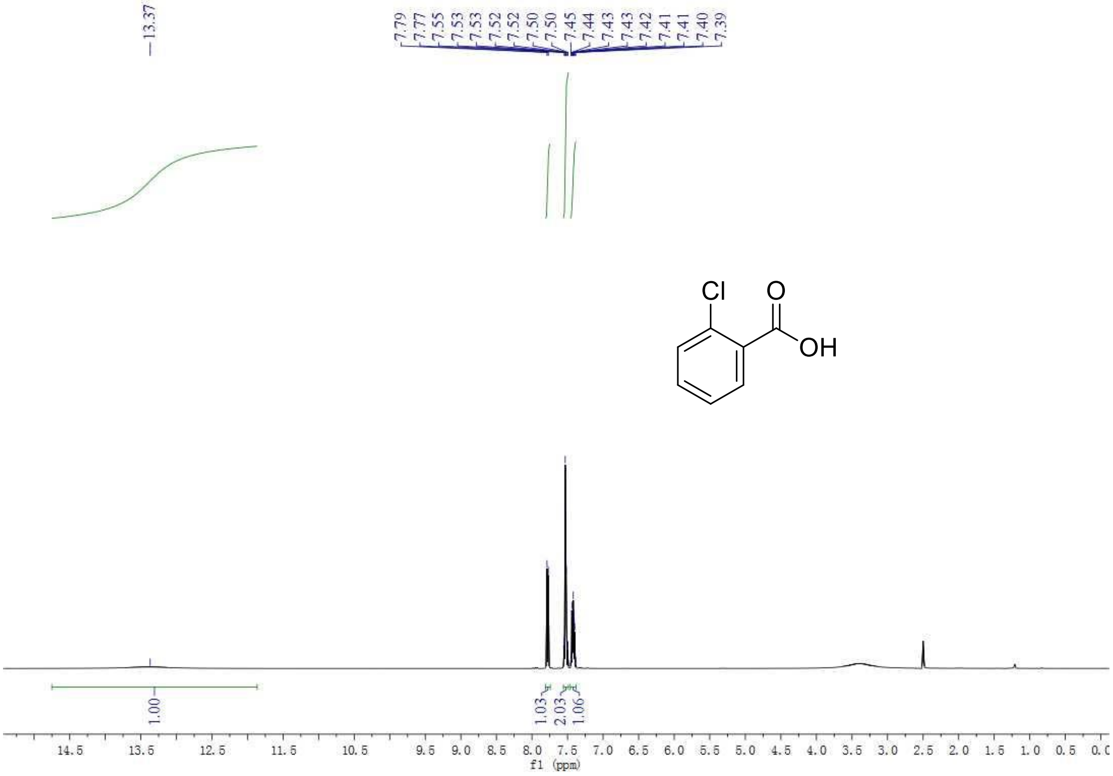

^{13}C{1H} NMR spectrum of 2-chlorobenzoic acid (3l, 101 MHz, DMSO-*d6*)

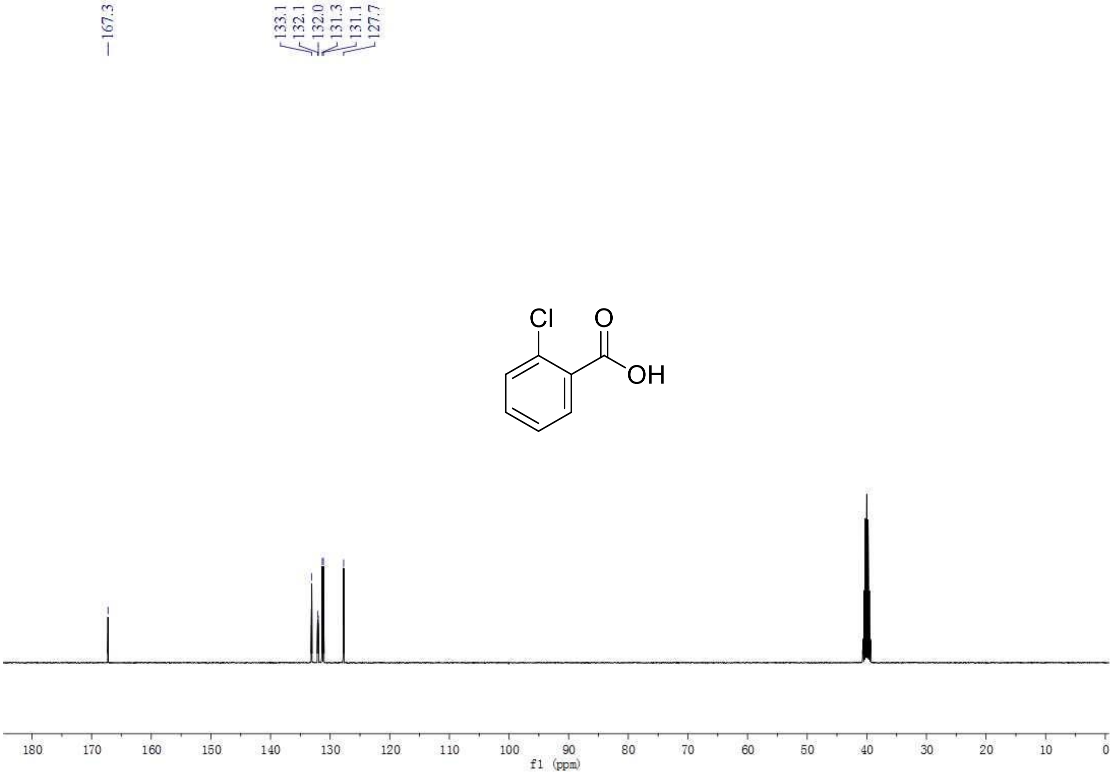

^{1}H NMR spectrum of 2-methylbenzoic acid (3m, 400 MHz, DMSO-*d6*)

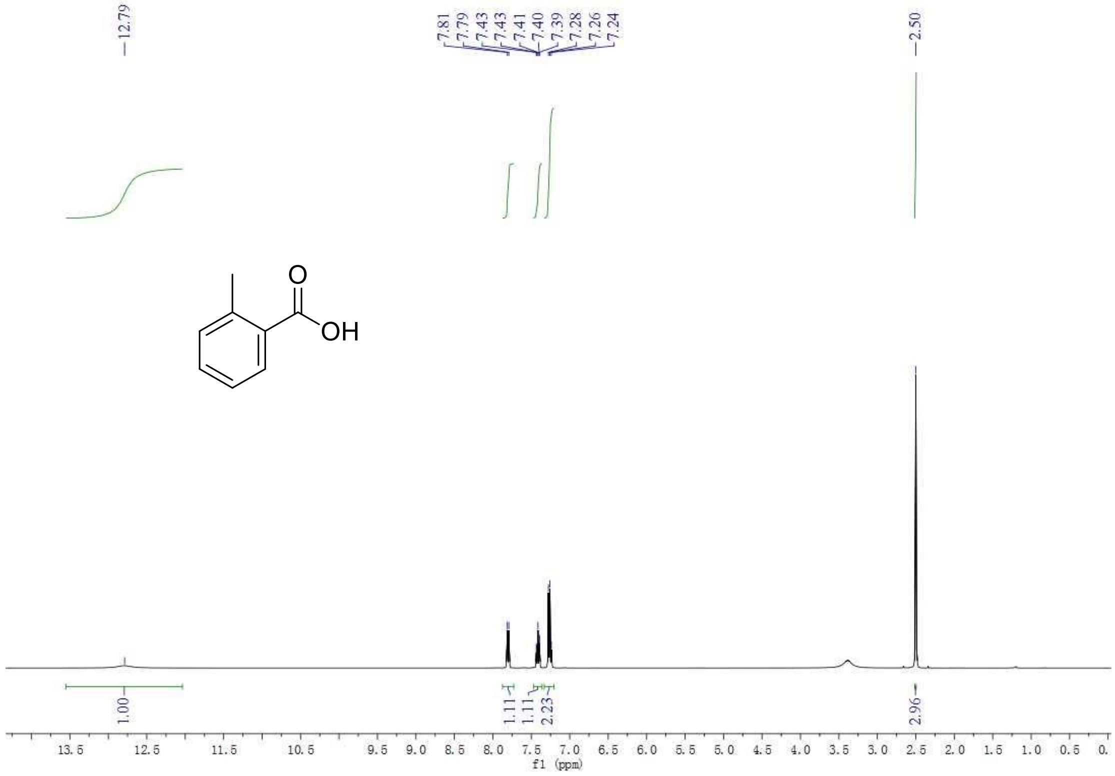

^{13}C{1H} NMR spectrum of 2-methylbenzoic acid (3m, 101 MHz, DMSO-*d6*)

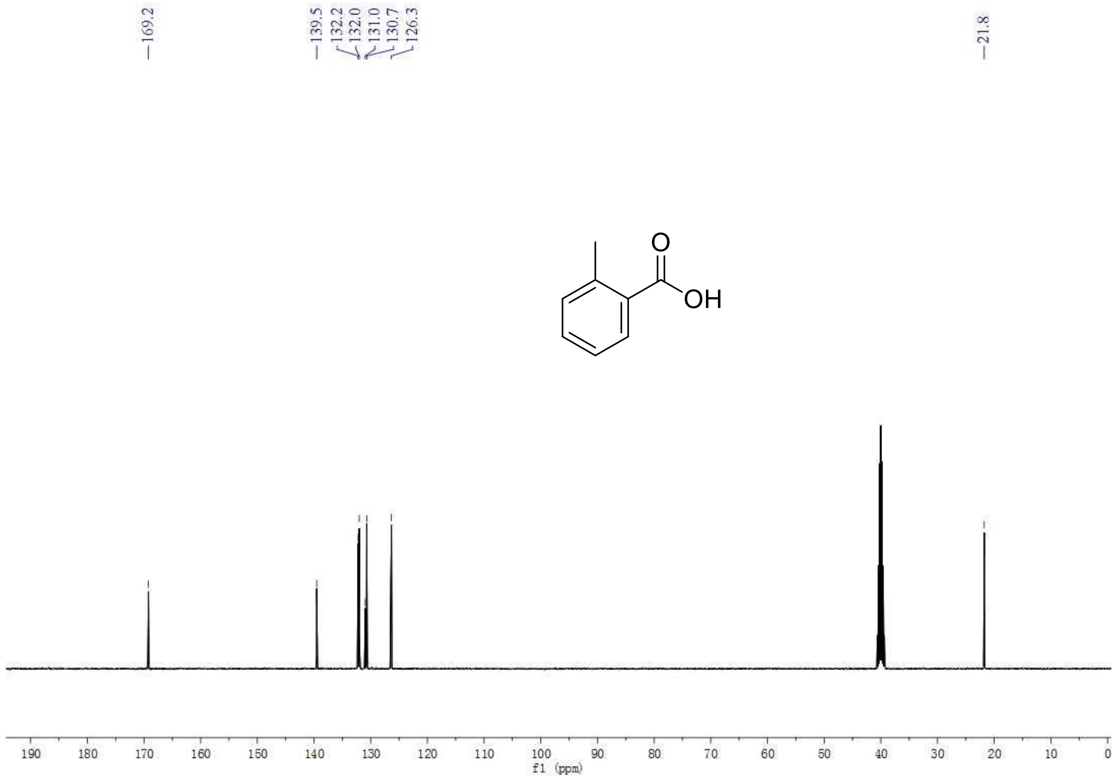

^{1}H NMR spectrum of 3-chlorobenzoic acid (3n, 400 MHz, DMSO-*d6*)

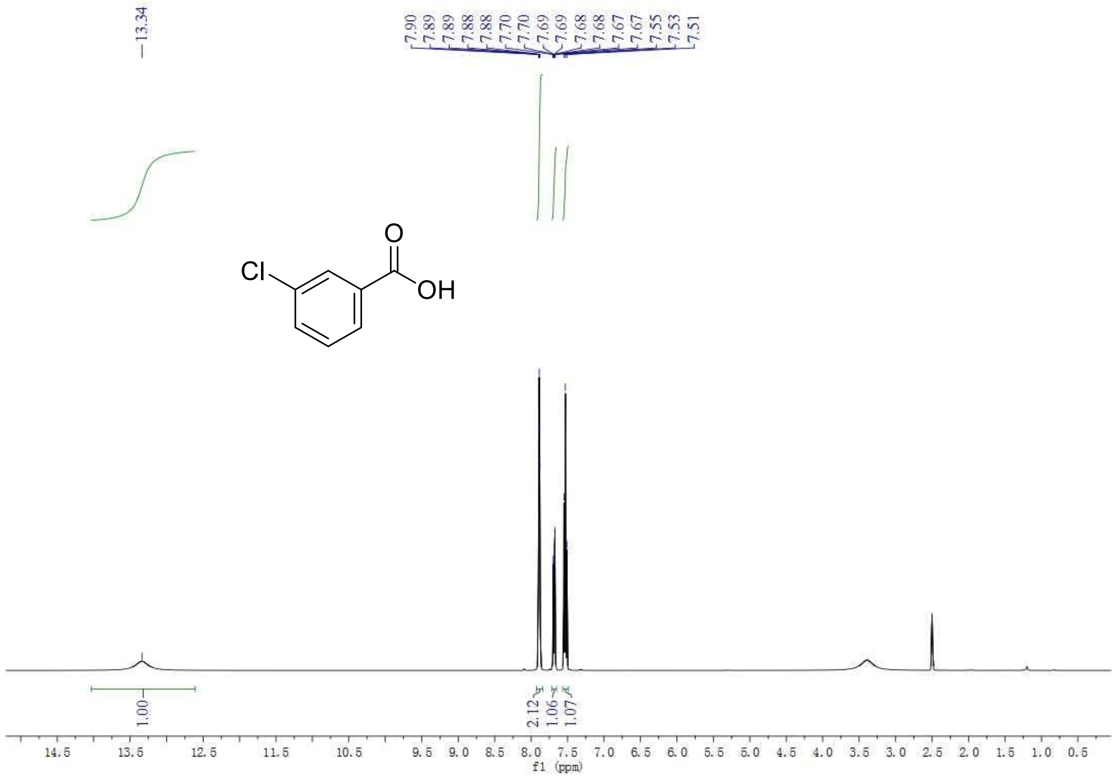

^{13}C{1H} NMR spectrum of 3-chlorobenzoic acid (3n, 101 MHz, DMSO-*d6*)

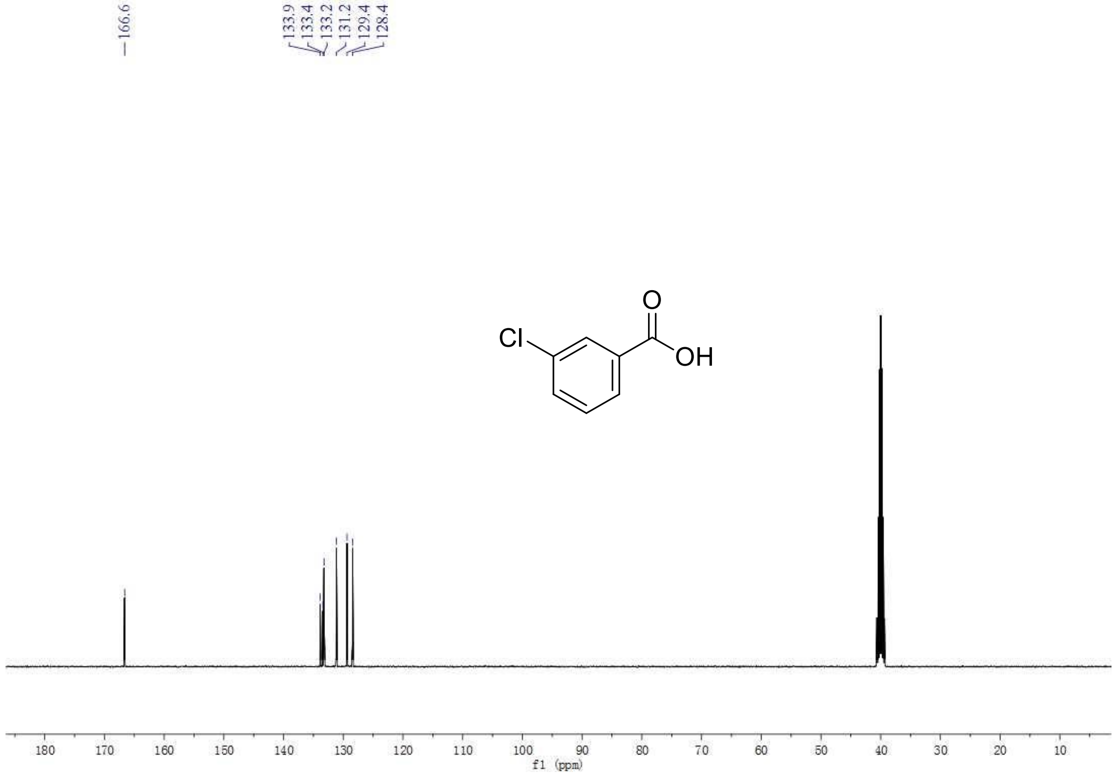

^{1}H NMR spectrum of 3-methylbenzoic acid (3o, 101 MHz, DMSO-*d6*)

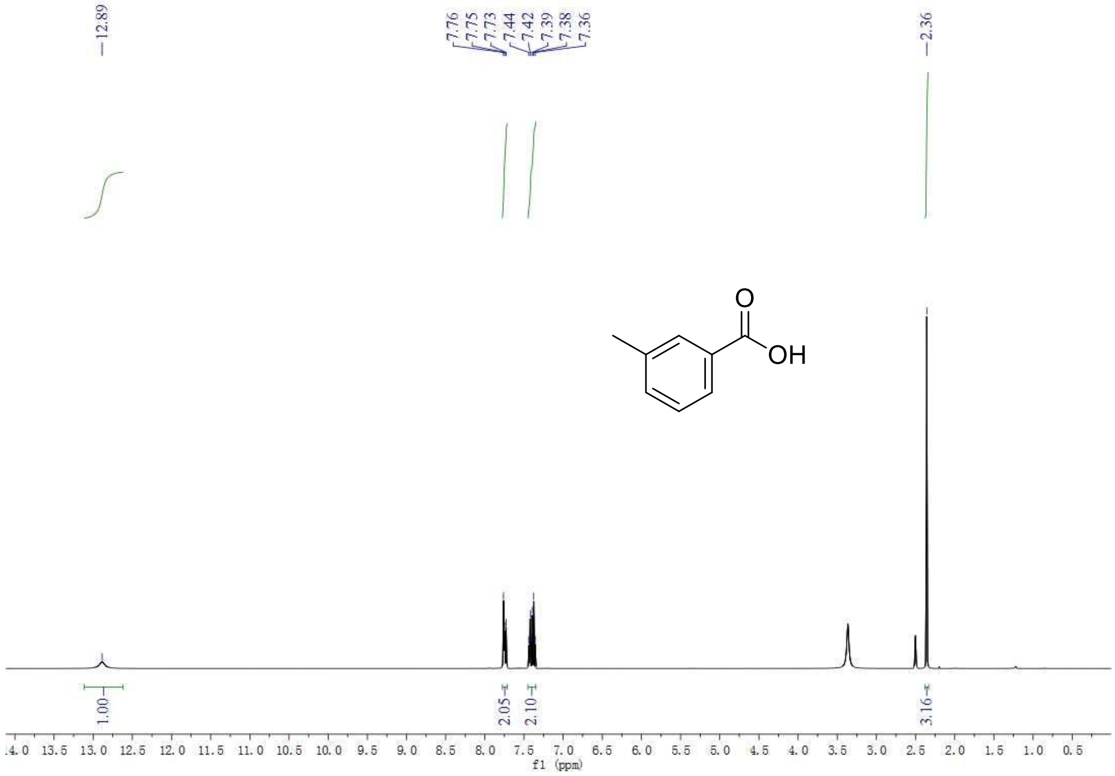

^{13}C{1H} NMR spectrum of 3-methylbenzoic acid (3o, 101 MHz, DMSO-*d6*)

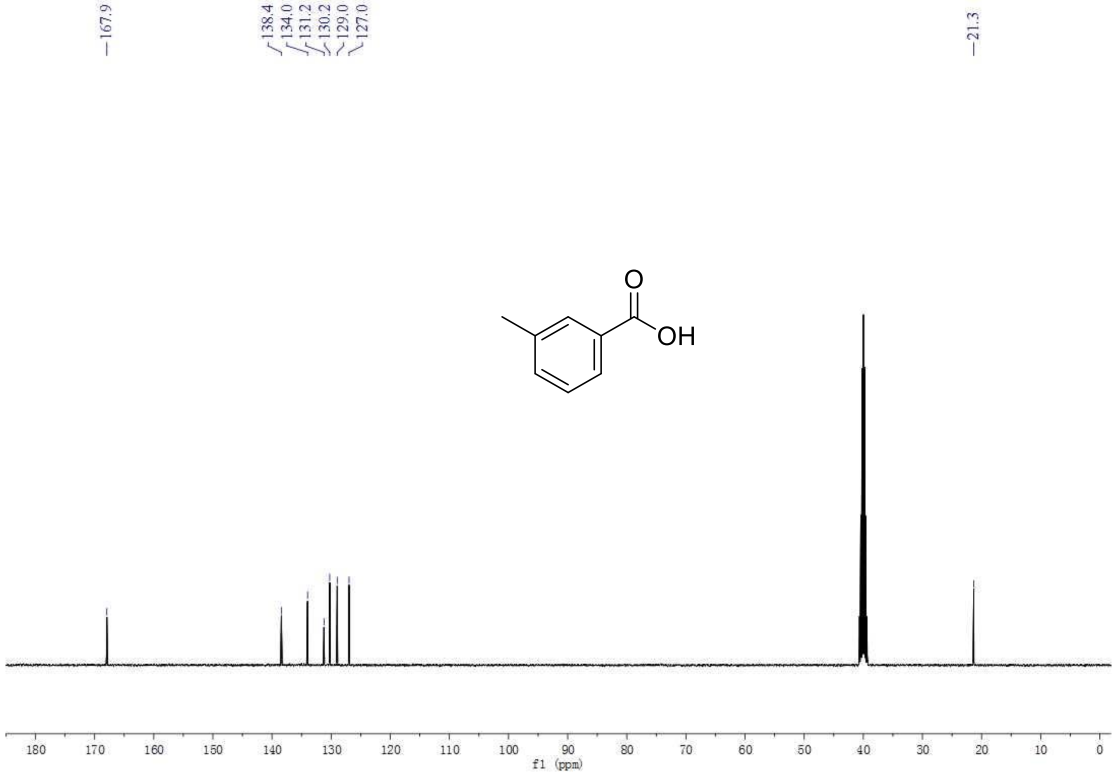

^{1}H NMR spectrum of 3,4-dichlorobenzoic acid (3p, 400 MHz, DMSO-*d6*)

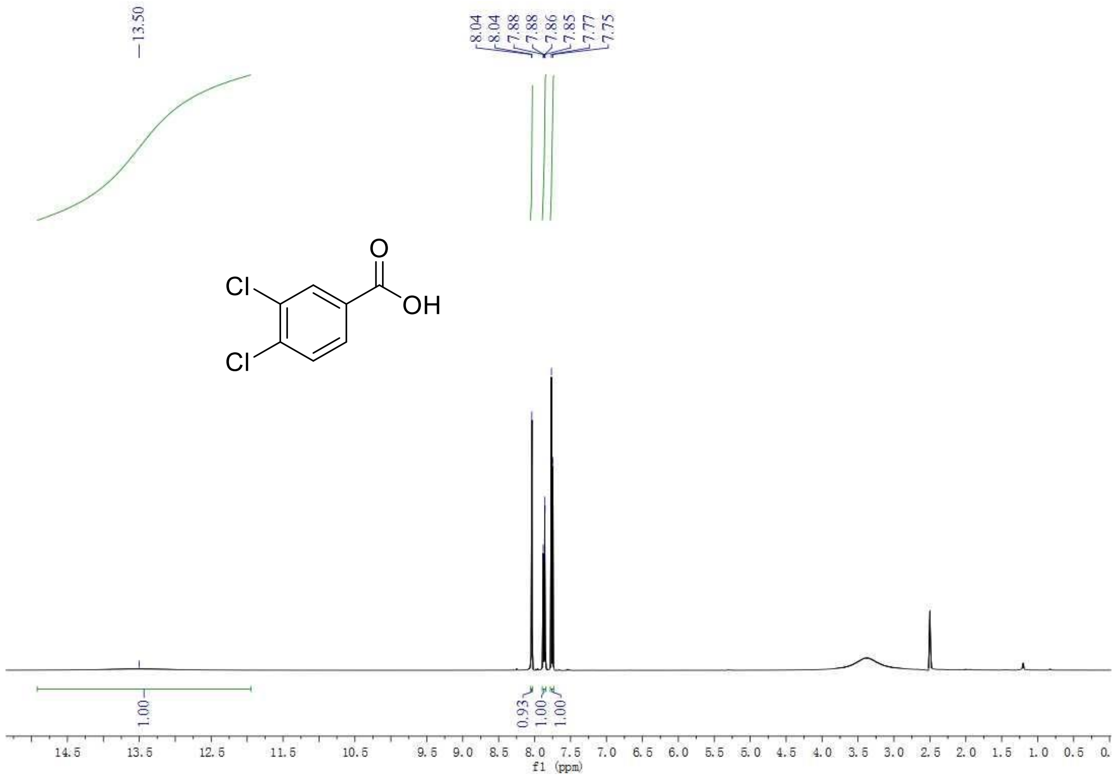

^{13}C{1H} NMR spectrum of 3,4-dichlorobenzoic acid (3p, 101 MHz, DMSO-*d6*)

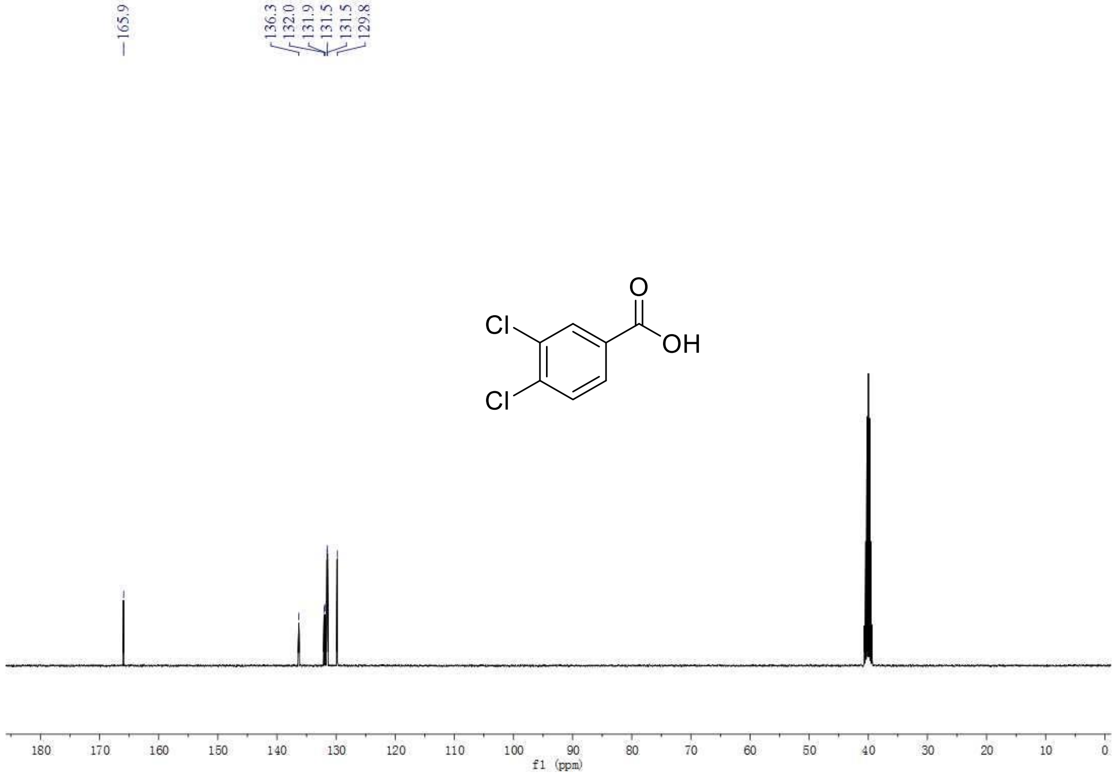

^{1}H NMR spectrum of terephthalic acid (3q, 400 MHz, DMSO-*d6*)

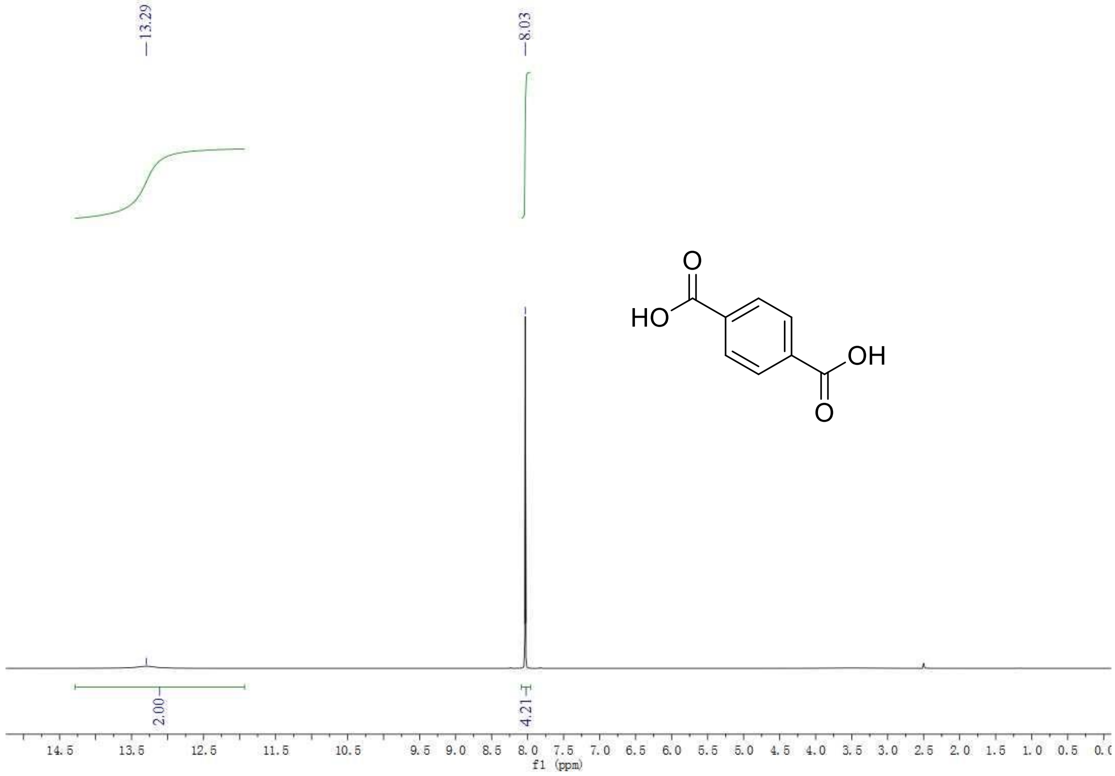

^{13}C{1H} NMR spectrum of terephthalic acid (3q, 101 MHz, DMSO-*d6*)

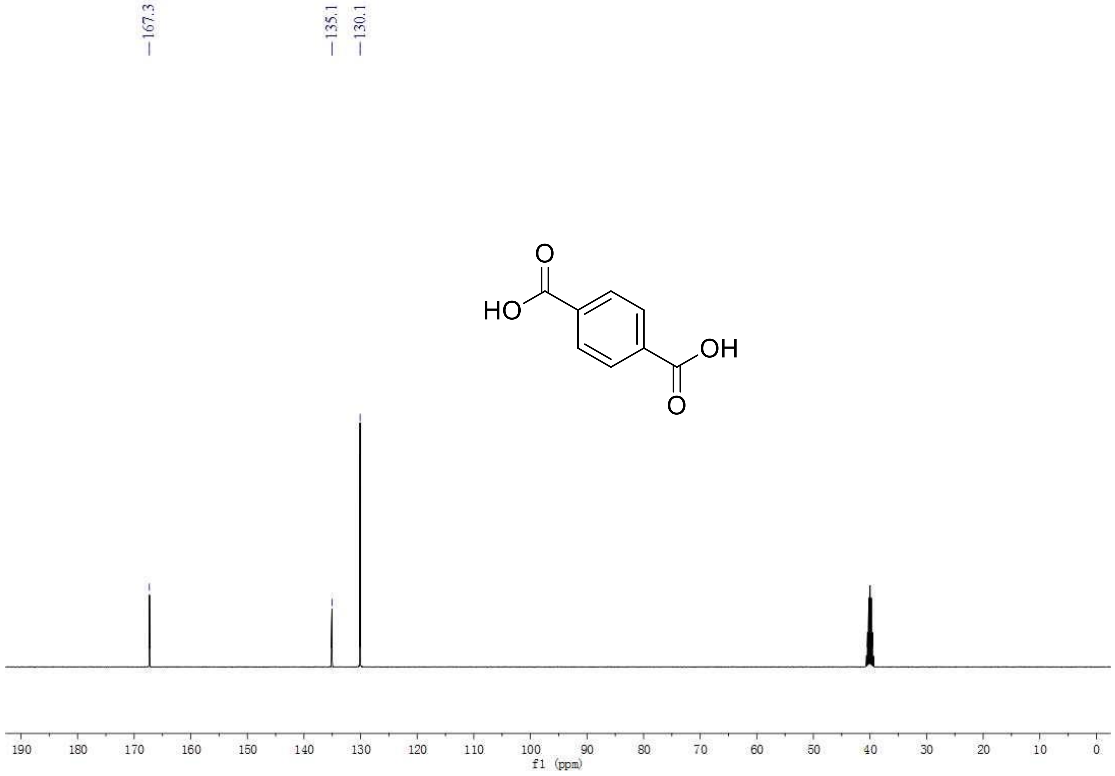

^{1}H NMR spectrum of 1-naphthoic acid (3r, 400 MHz, DMSO-*d6*)

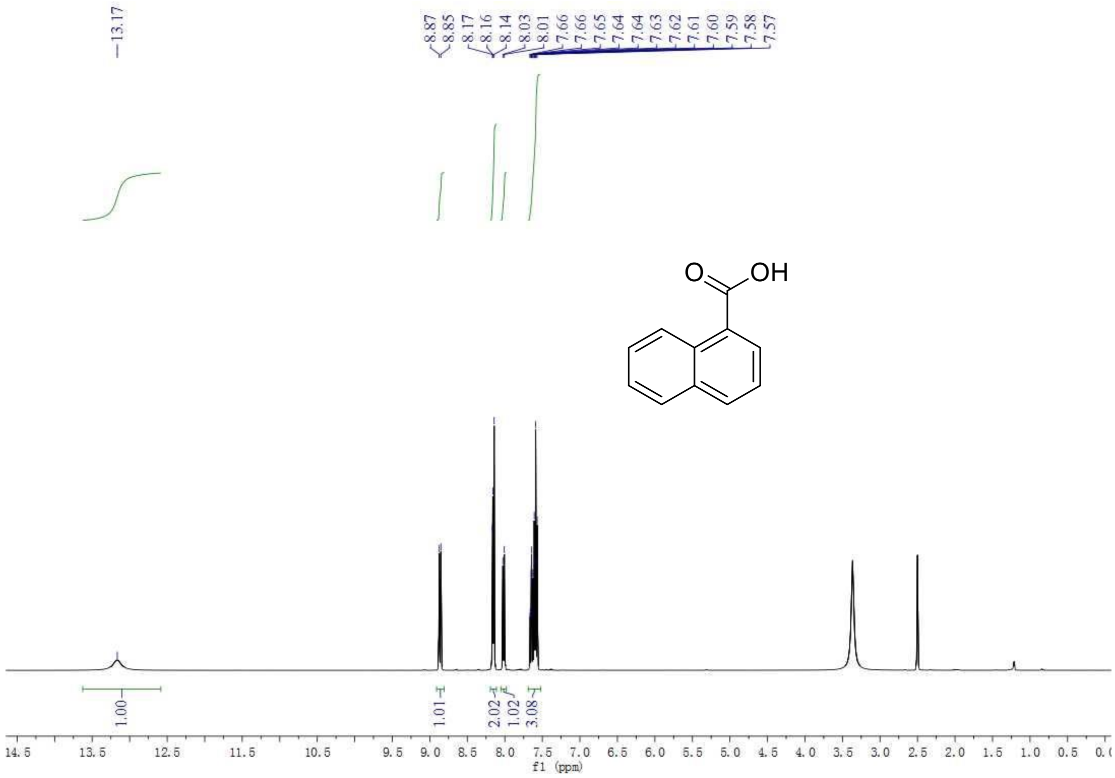

^{13}C{1H} NMR spectrum of 1-naphthoic acid (3r, 101 MHz, DMSO-*d6*)

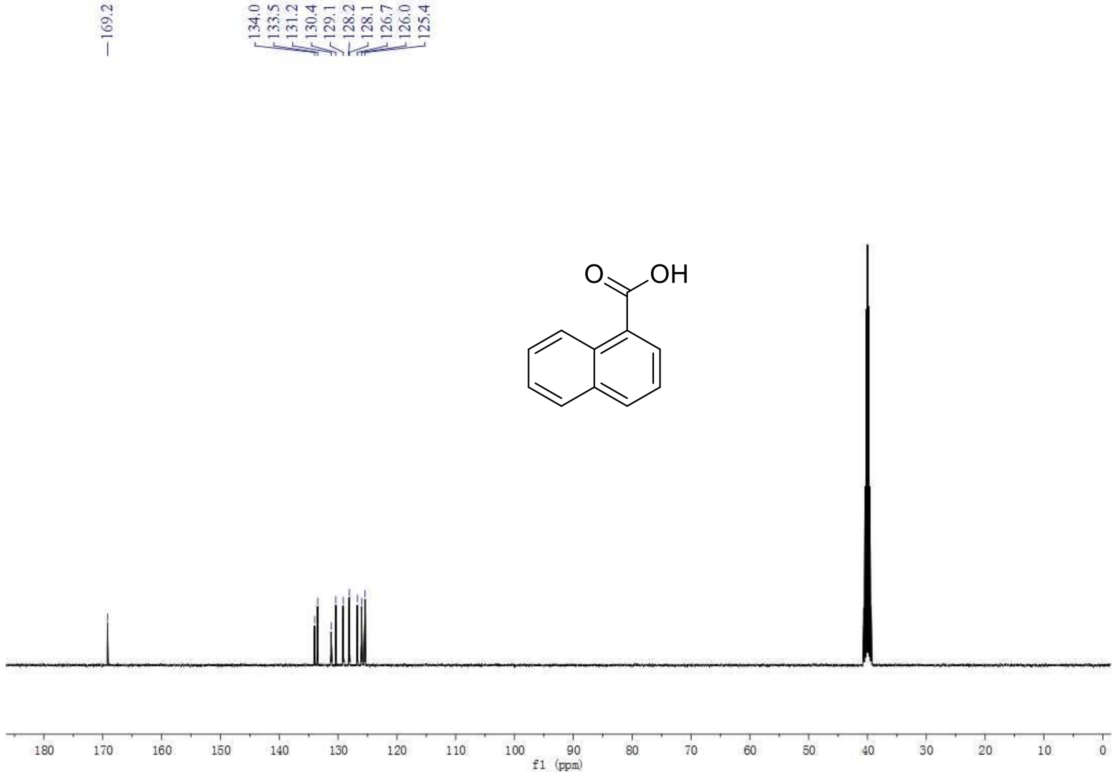

^{1}H NMR spectrum of anthracene-9-carboxylic acid (3s, 400 MHz, DMSO-*d6*)

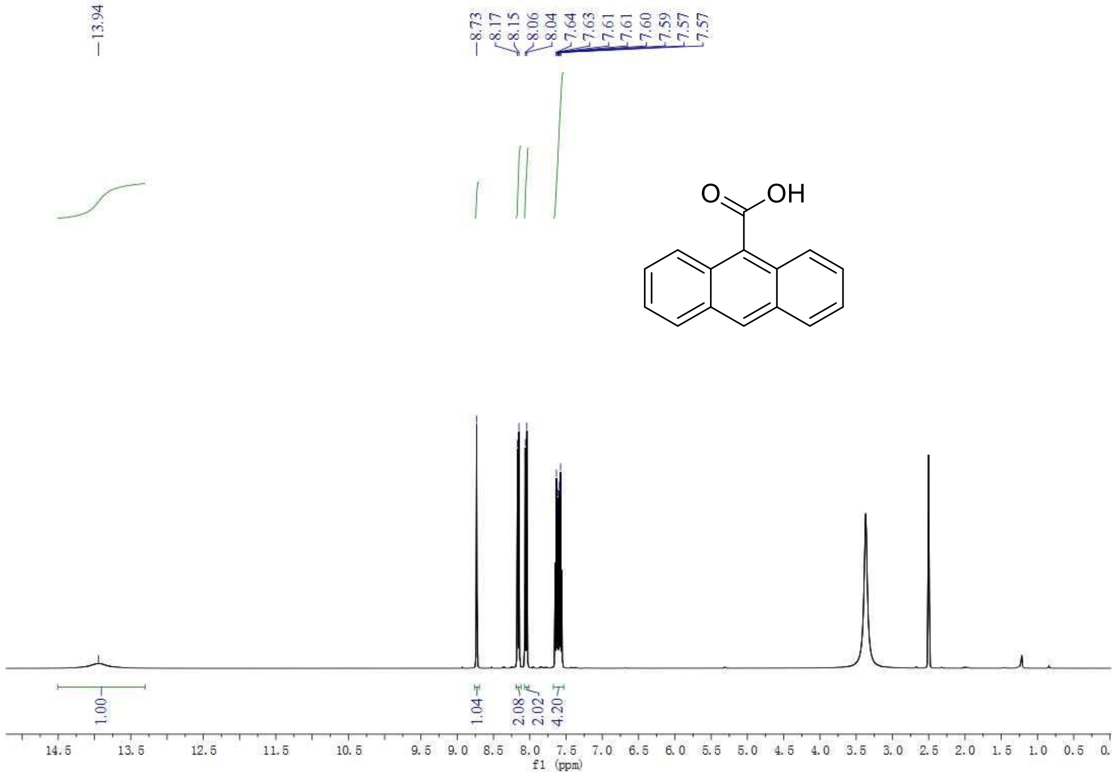

^{13}C{1H} NMR spectrum of anthracene-9-carboxylic acid (3s, 101 MHz, DMSO-*d6*)

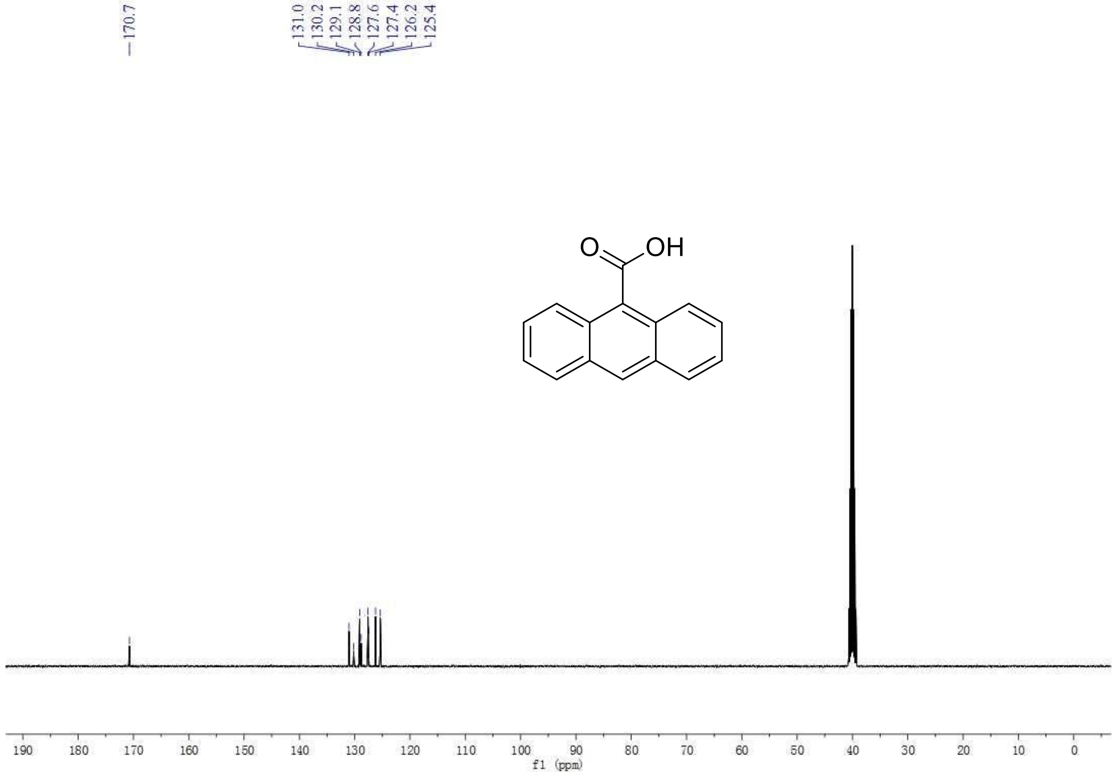

^{1}H NMR spectrum of isonicotinic acid (3t 400 MHz, DMSO-*d6*)

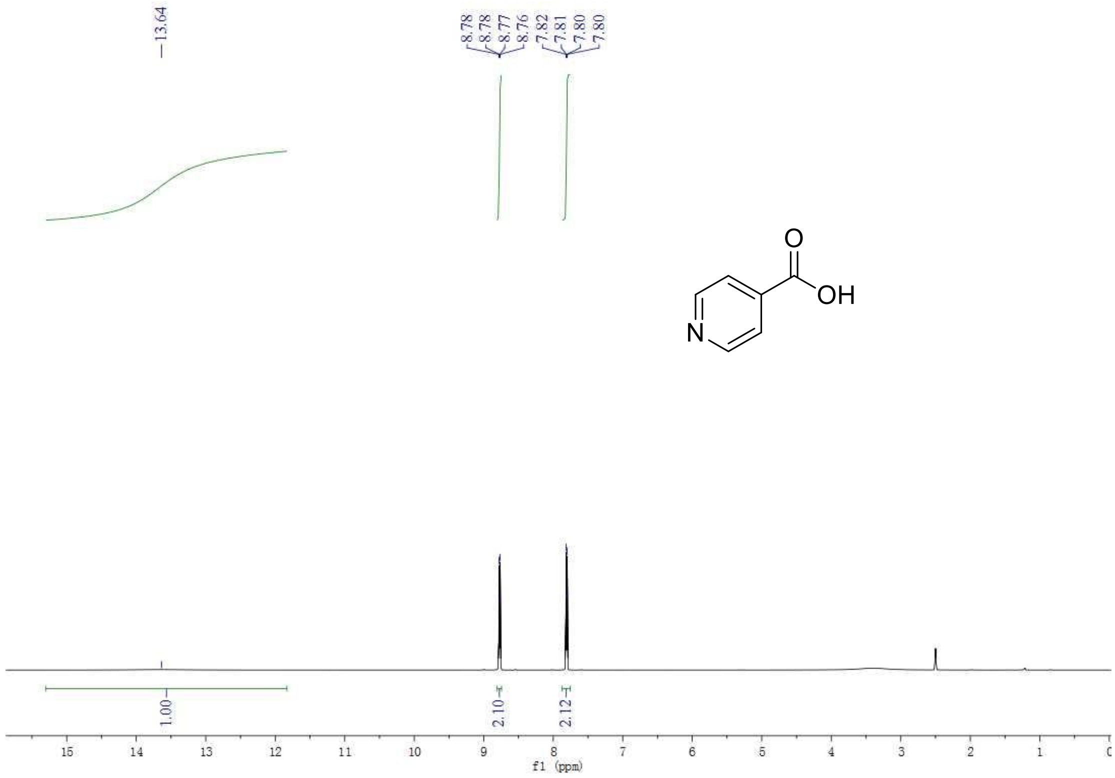

^{13}C{1H} NMR spectrum of isonicotinic acid (3t, 101 MHz, DMSO-*d6*)

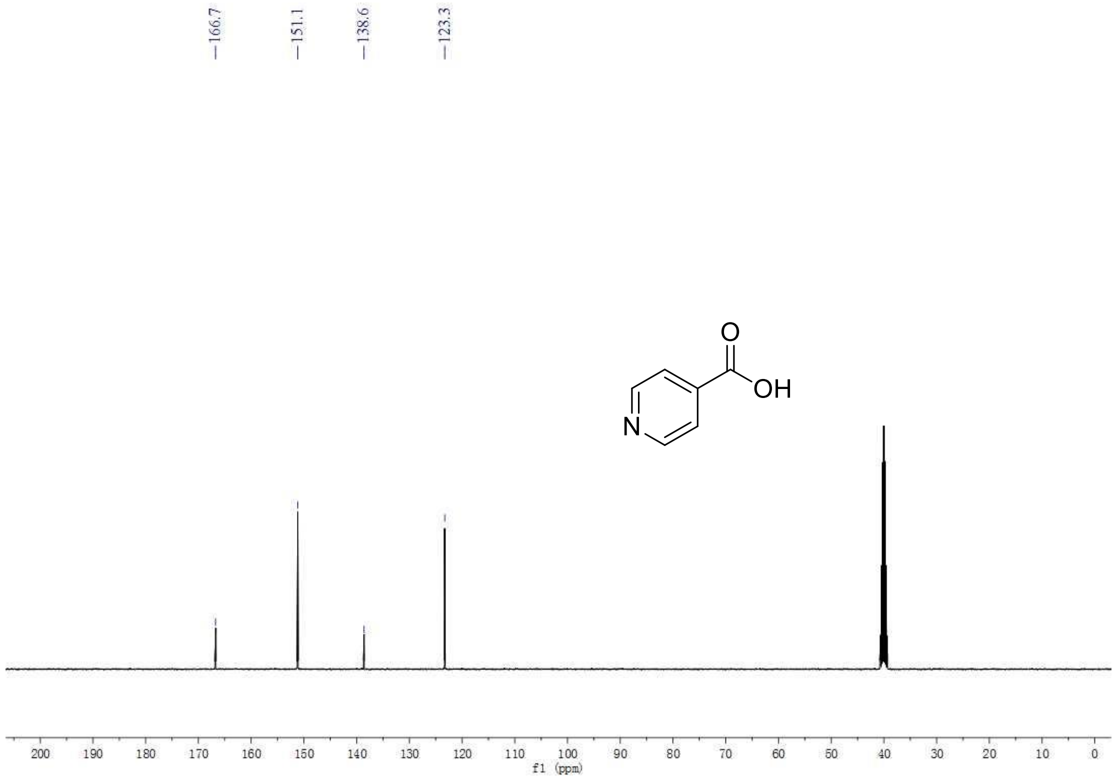

^{1}H NMR spectrum of thiophene-2-carboxylic acid (3u, 400 MHz, DMSO-*d6*)

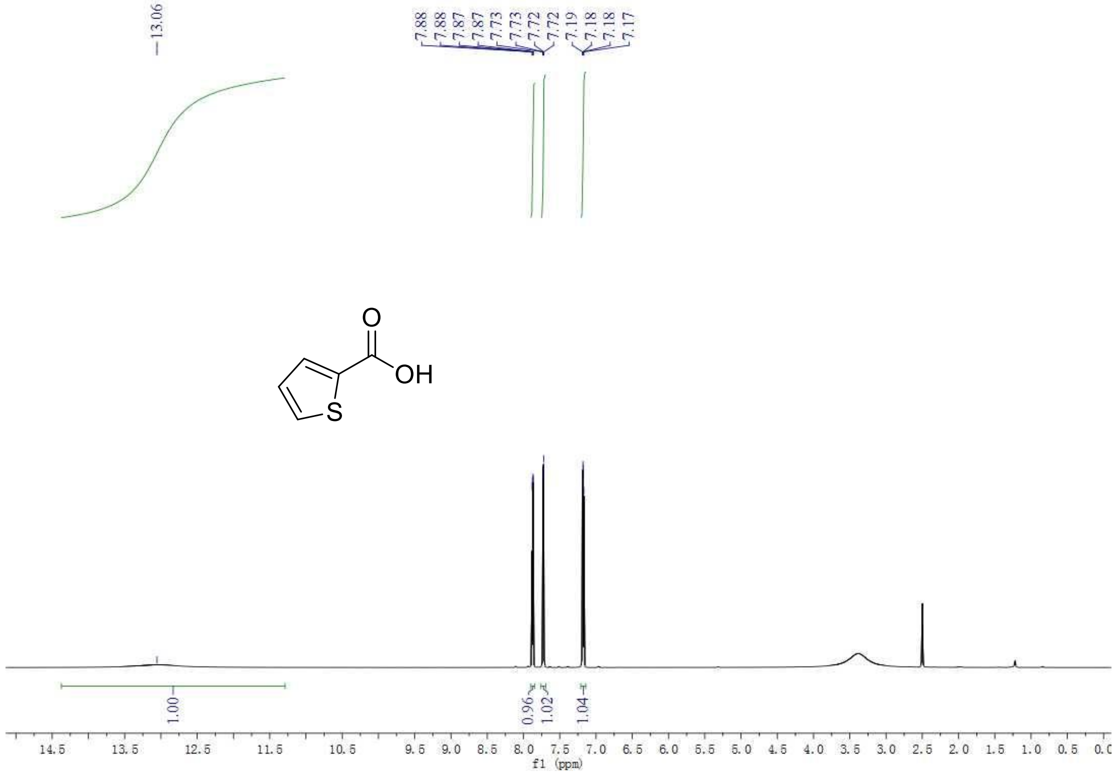

^{13}C{1H} NMR spectrum of thiophene-2-carboxylic acid (3u, 101 MHz, DMSO-*d6*)

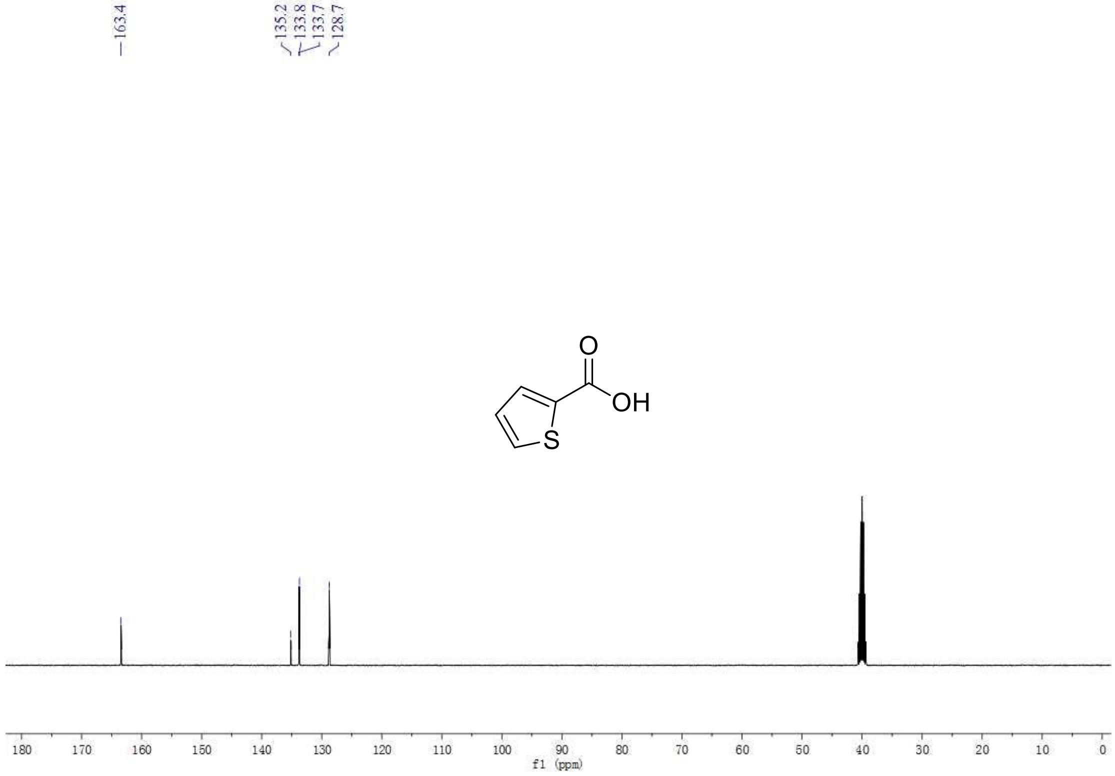

^{1}H NMR spectrum of isobenzofuran-1(3H)-one (3v, 400 MHz, $CDCl_3$)

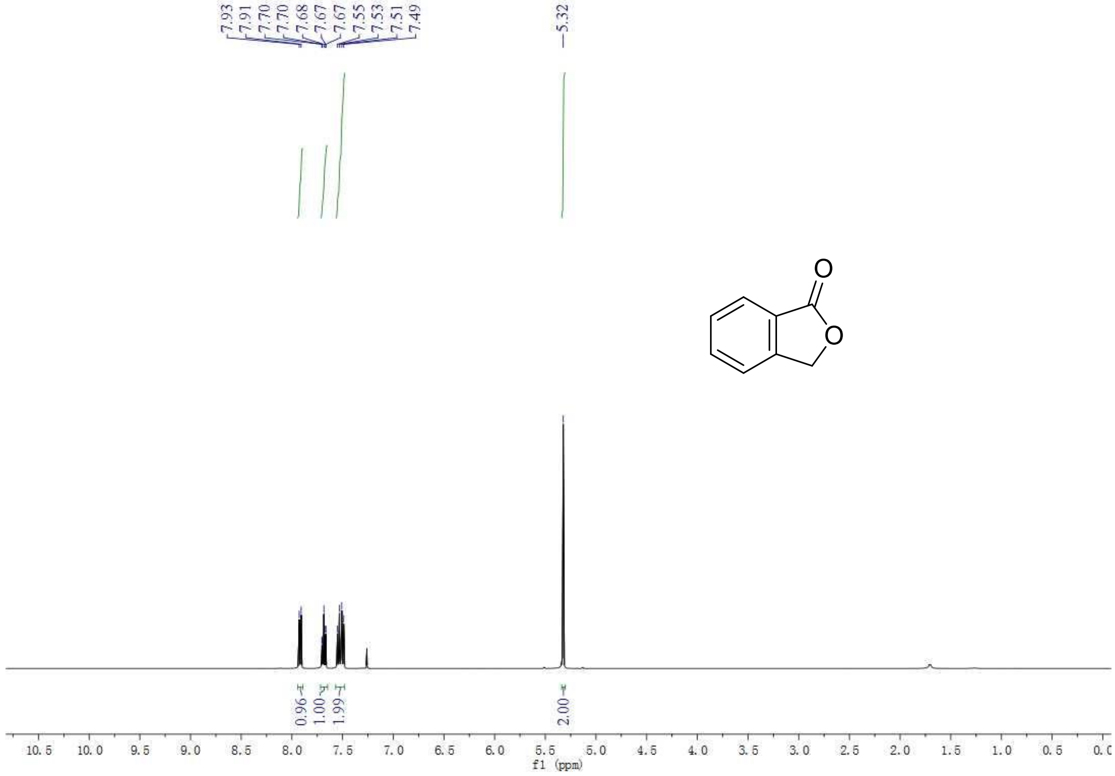

^{13}C{1H} NMR spectrum of isobenzofuran-1(3H)-one (3v, 101 MHz, $CDCl_3$)

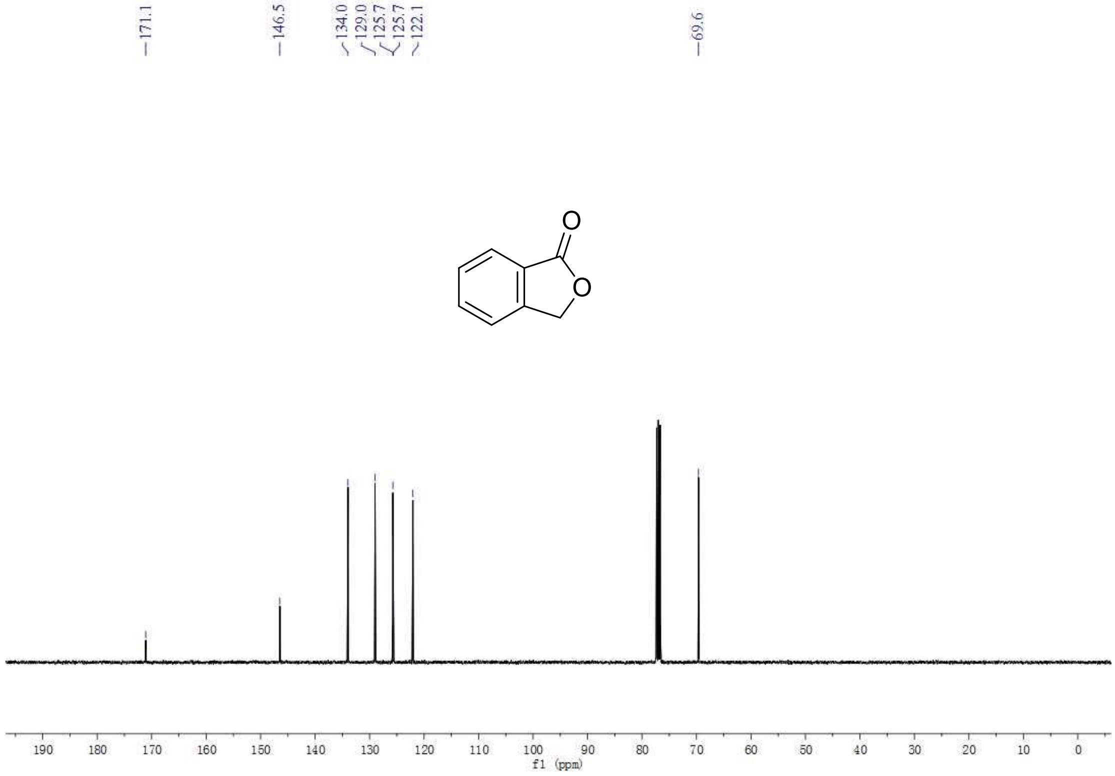

^{1}H **NMR spectrum of 2-((3-(trifluoromethyl)phenyl)amino)nicotinic acid (3y, 400 MHz, DMSO-*d6*)**

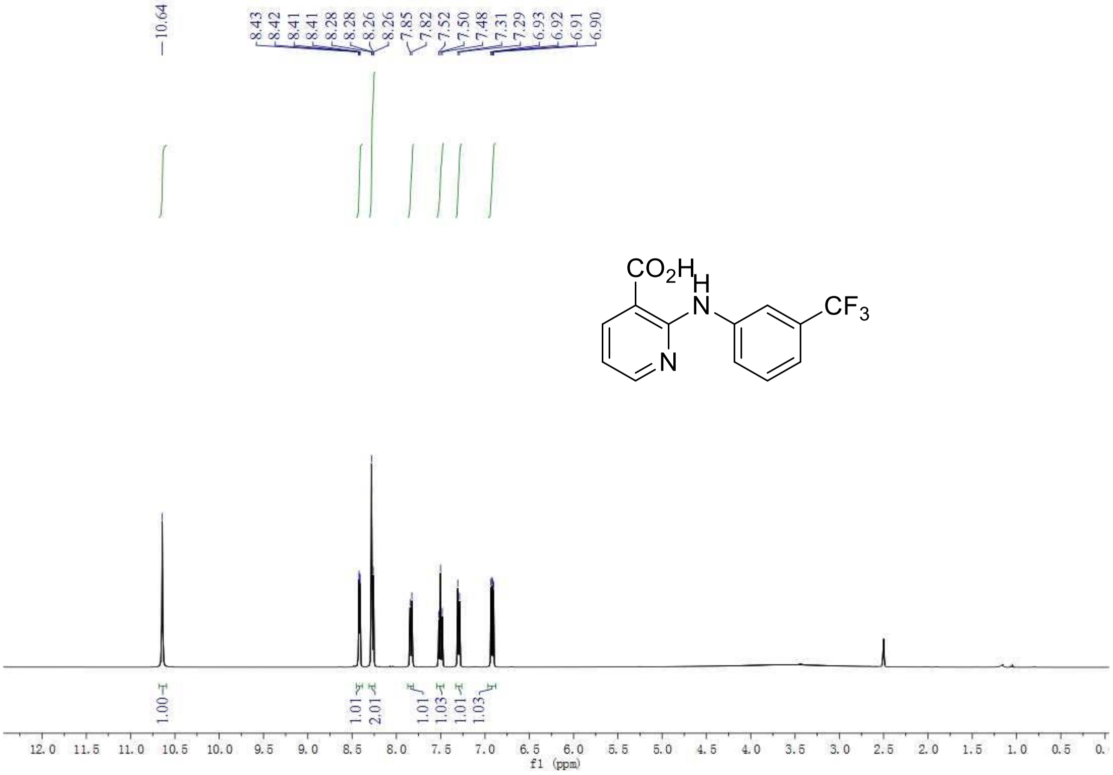

$^{13}C\{1H\}$ **NMR spectrum of2-((3-(trifluoromethyl)phenyl)amino)nicotinic acid (3w, 101 MHz, DMSO-*d6*)**

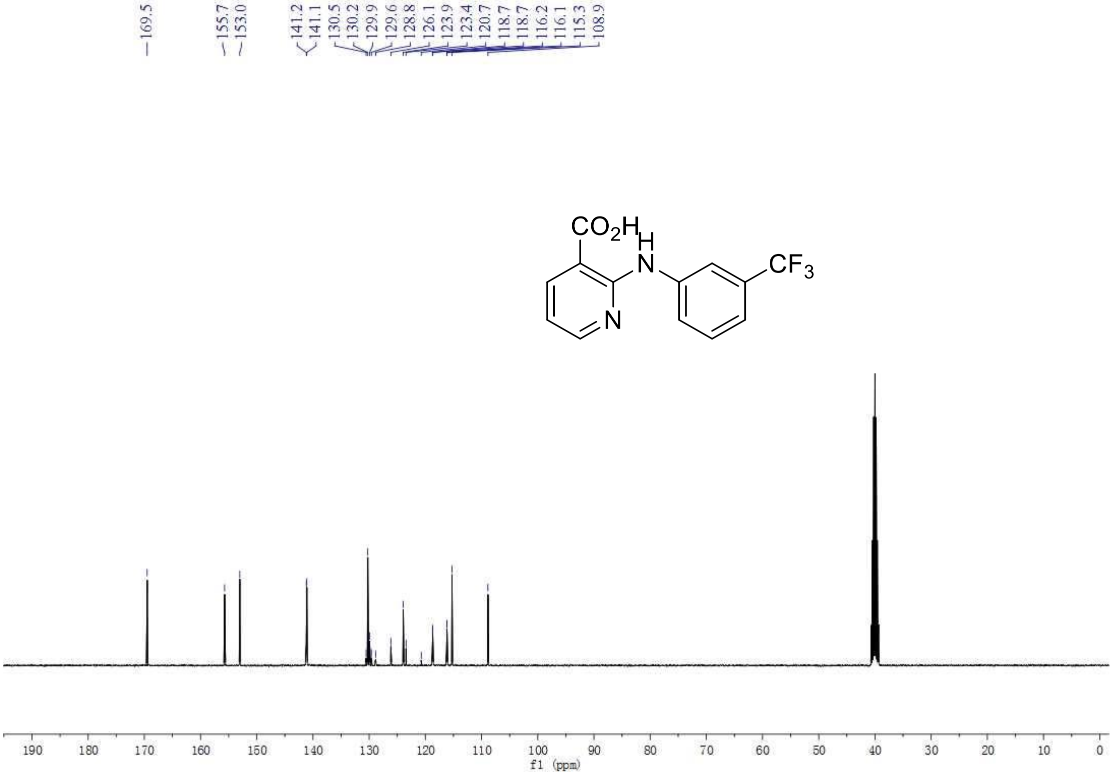

^{19}F NMR spectrum of 2-((3-(trifluoromethyl)phenyl)amino)nicotinic acid (3w, 376 MHz, DMSO-*d6*)

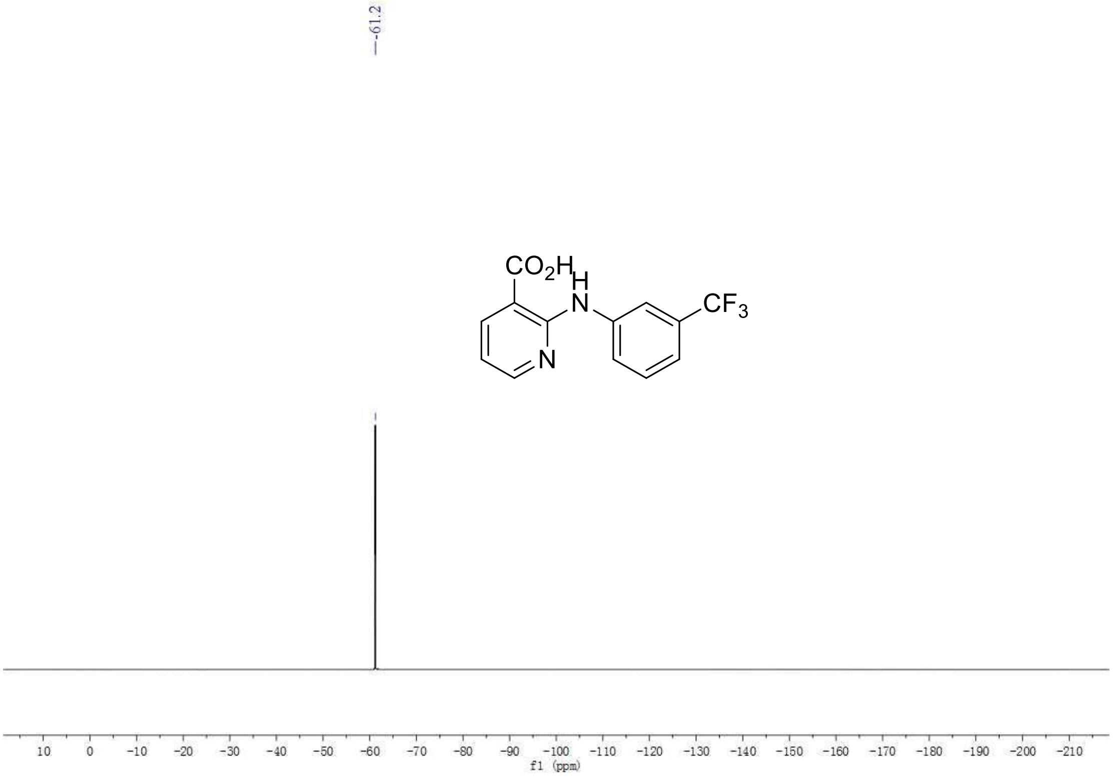

^{1}H NMR spectrum of acetophenone (4a, 400 MHz, CDCl$_3$)

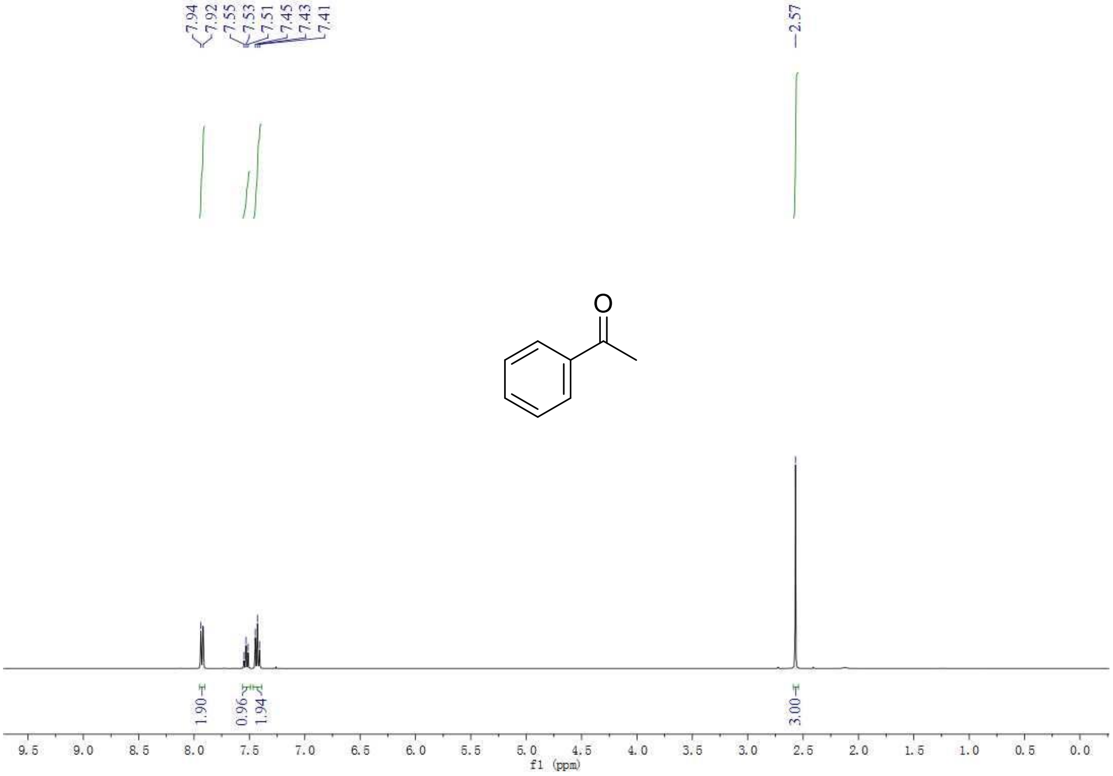

^{13}C{1H} NMR spectrum of acetophenone (4a, 101 MHz, $CDCl_3$)

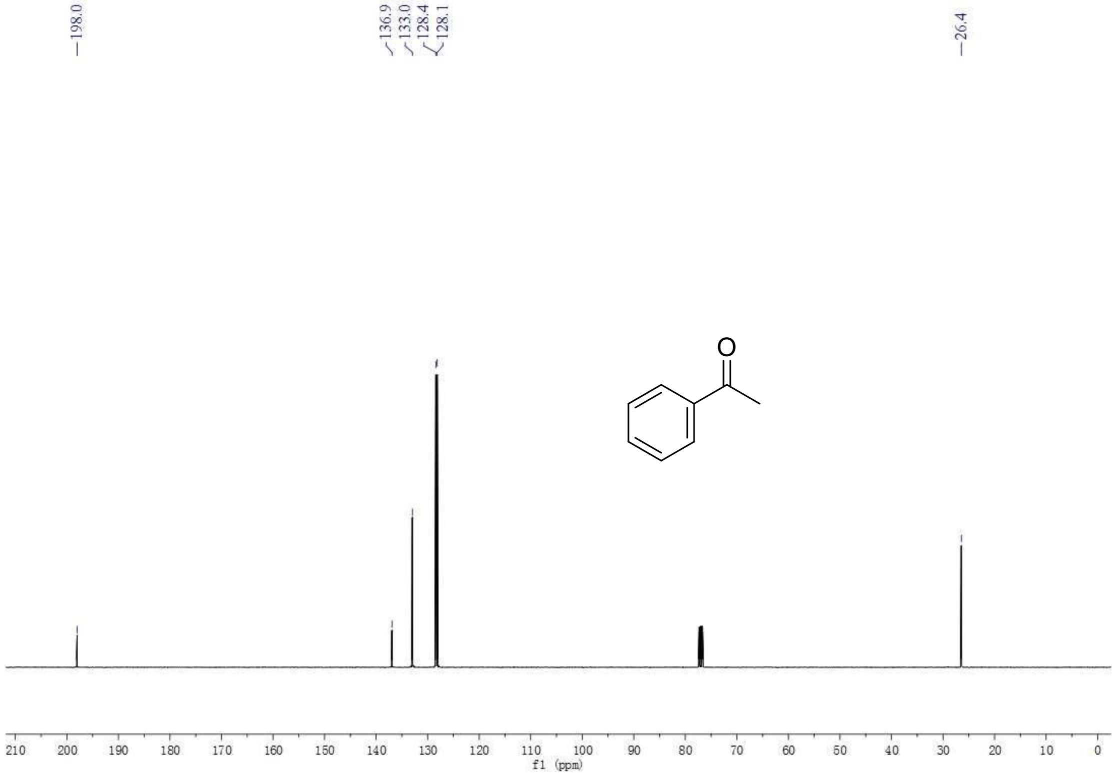

^{1}H NMR spectrum of 1-(*p*-tolyl)ethanone (4b, 400 MHz, $CDCl_3$)

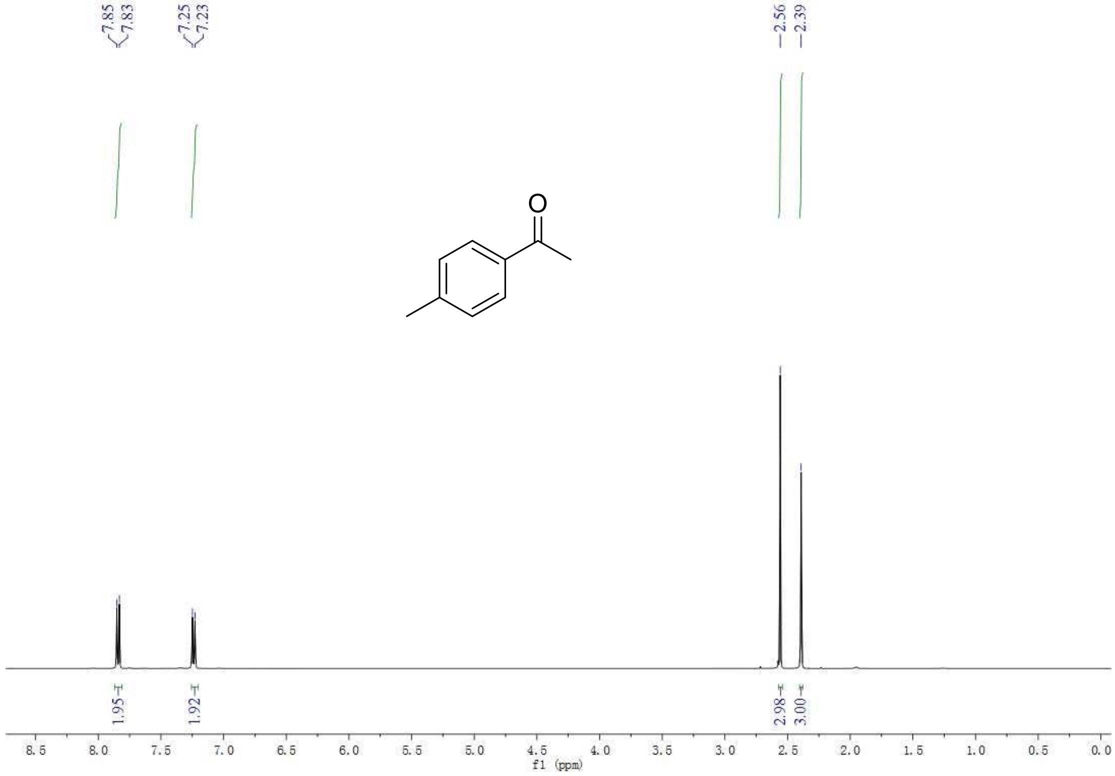

$^{13}C\{1H\}$ NMR spectrum of 1-(*p*-tolyl)ethanone (4b, 101 MHz, $CDCl_3$)

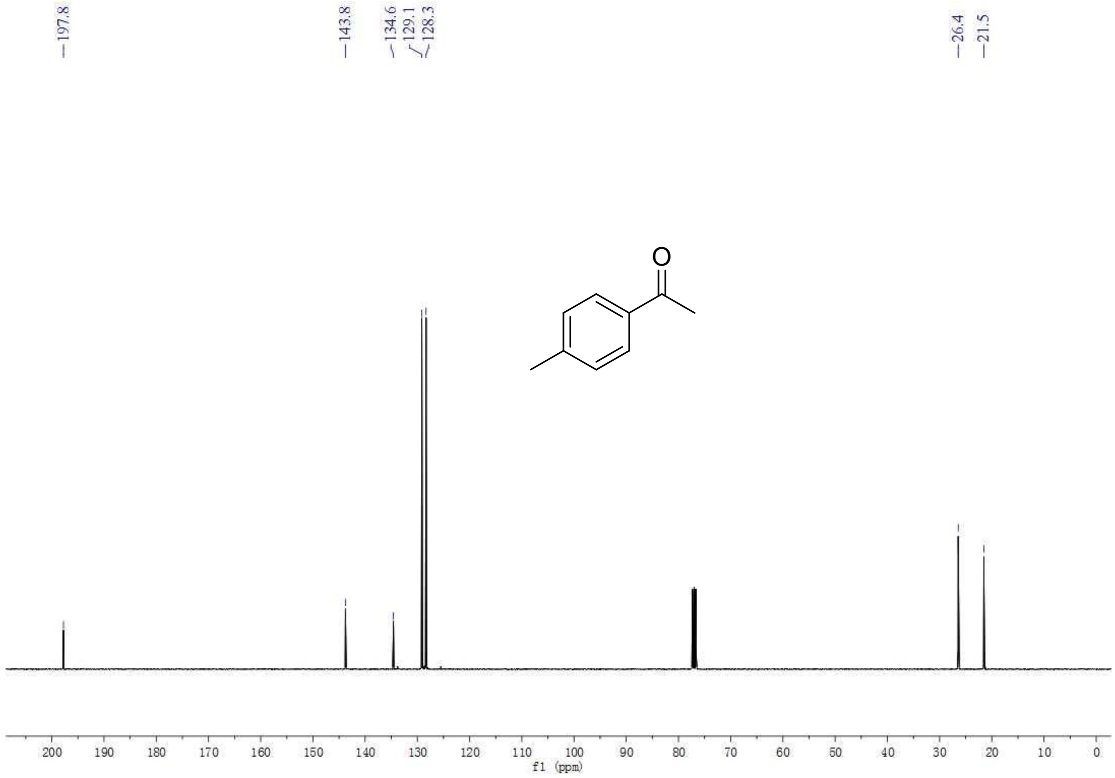

^{1}H NMR spectrum of 1-(4-fluorophenyl)ethanone (4c, 400 MHz, $CDCl_3$)

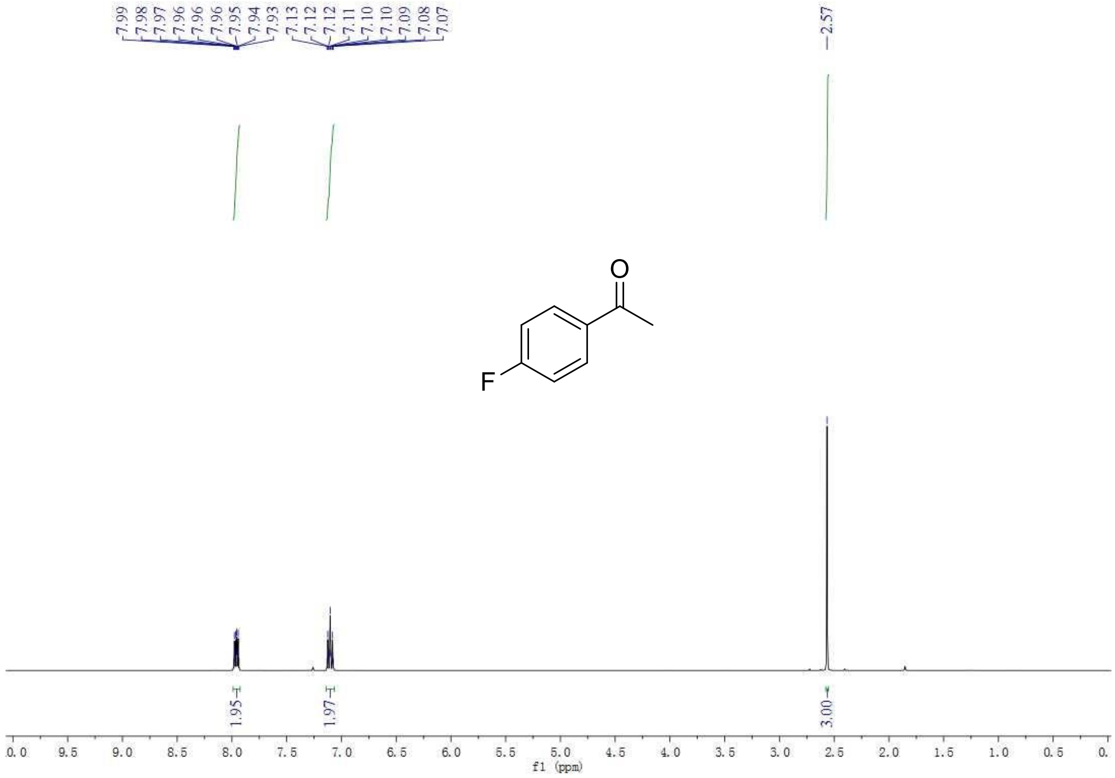

^{13}C{1H} NMR spectrum of 1-(4-fluorophenyl)ethanone (4c, 101 MHz, $CDCl_3$)

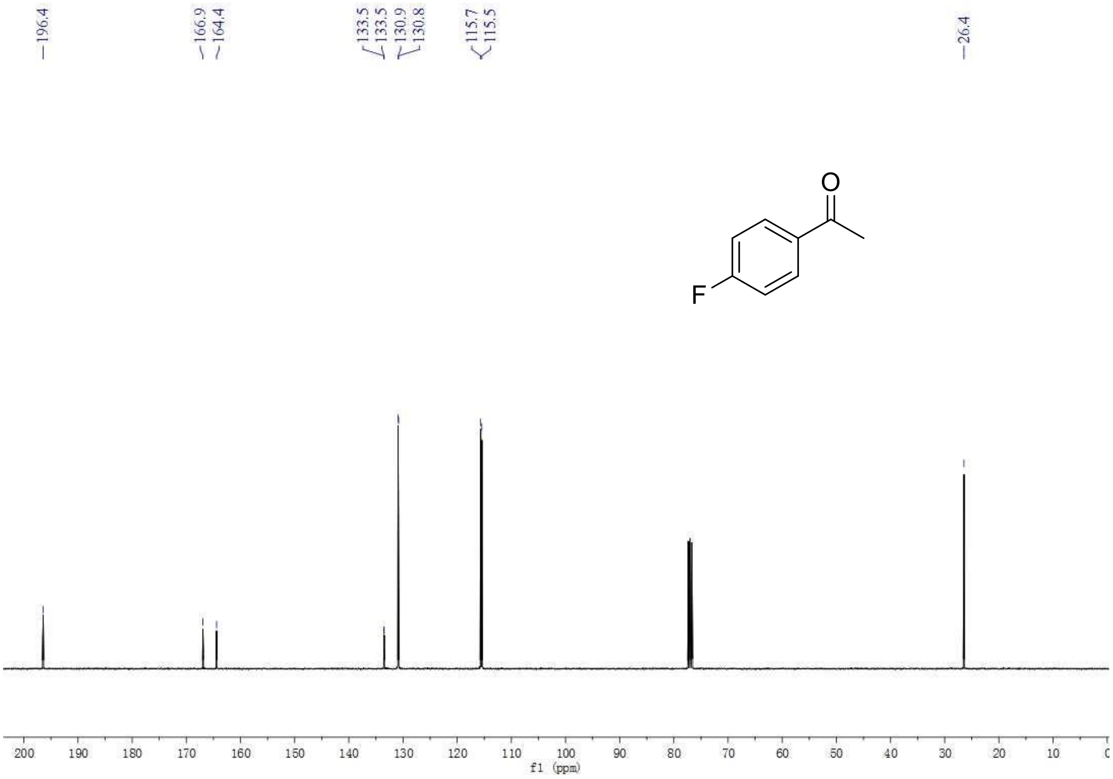

^{19}F NMR spectrum of 1-(4-fluorophenyl)ethanone (4c, 376 MHz, $CDCl_3$)

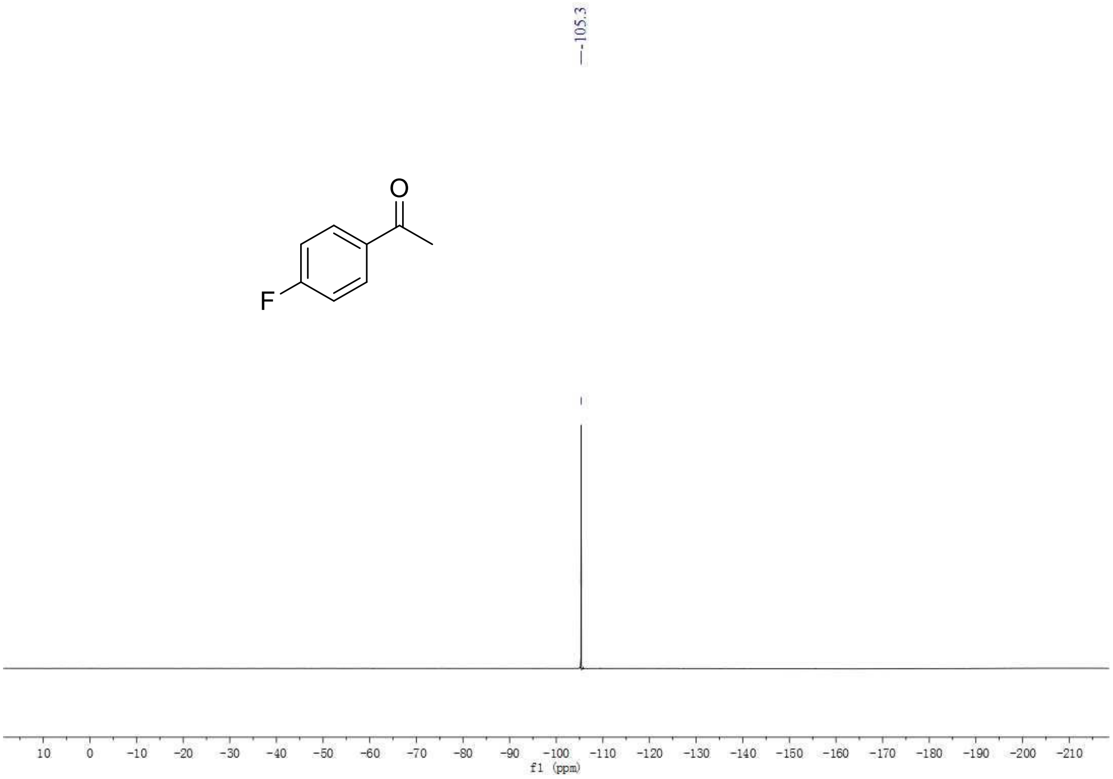

^{1}H NMR spectrum of acetophenone (4d, 400 MHz, $CDCl_3$)

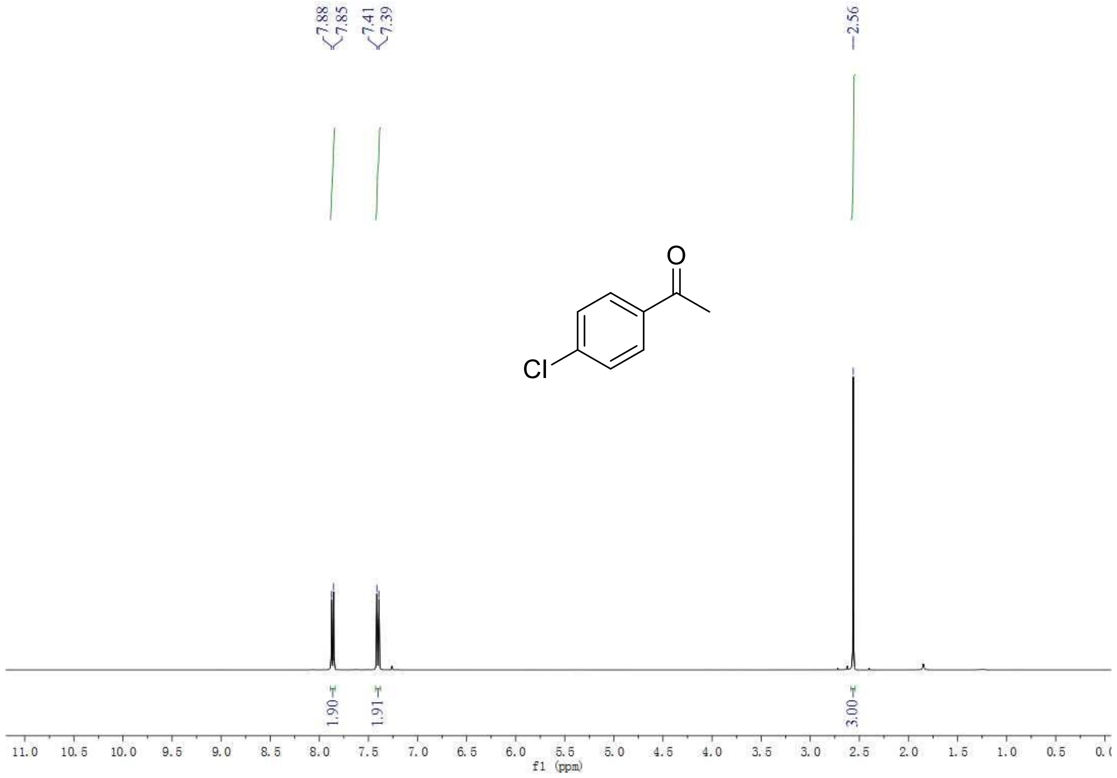

^{13}C{1H} NMR spectrum of acetophenone (4d, 101 MHz, $CDCl_3$)

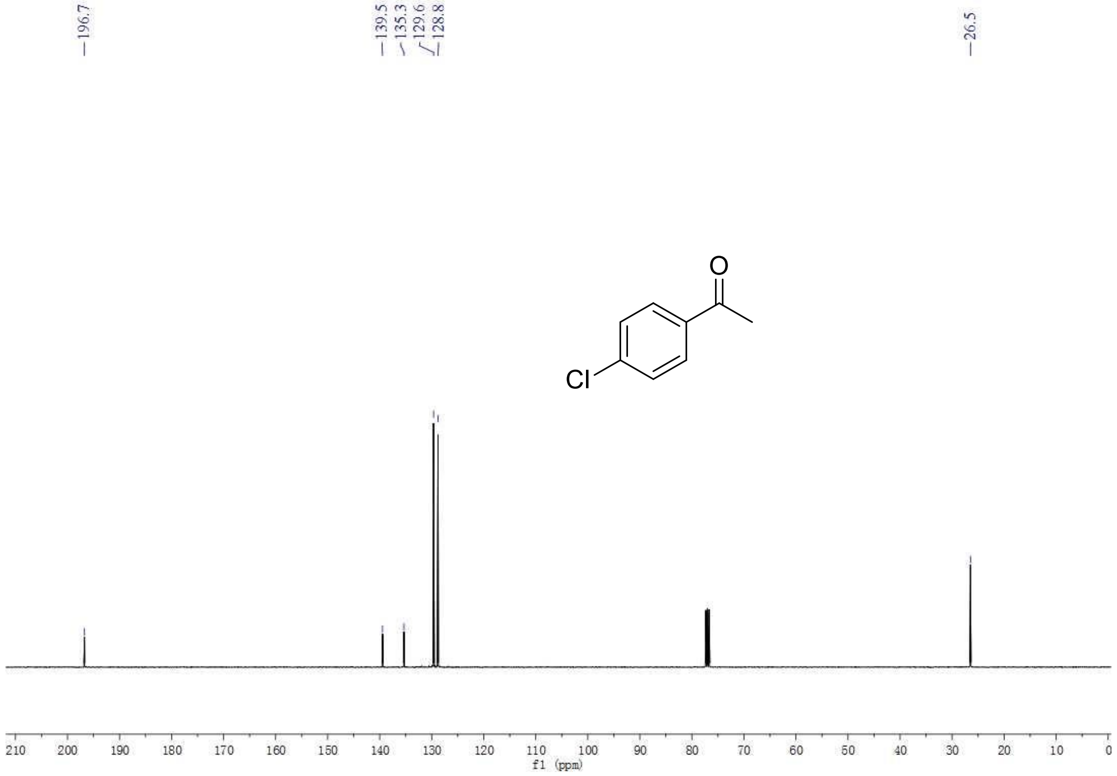

^{1}H NMR spectrum of 1-(4-bromophenyl)ethanone (4e, 400 MHz, $CDCl_3$)

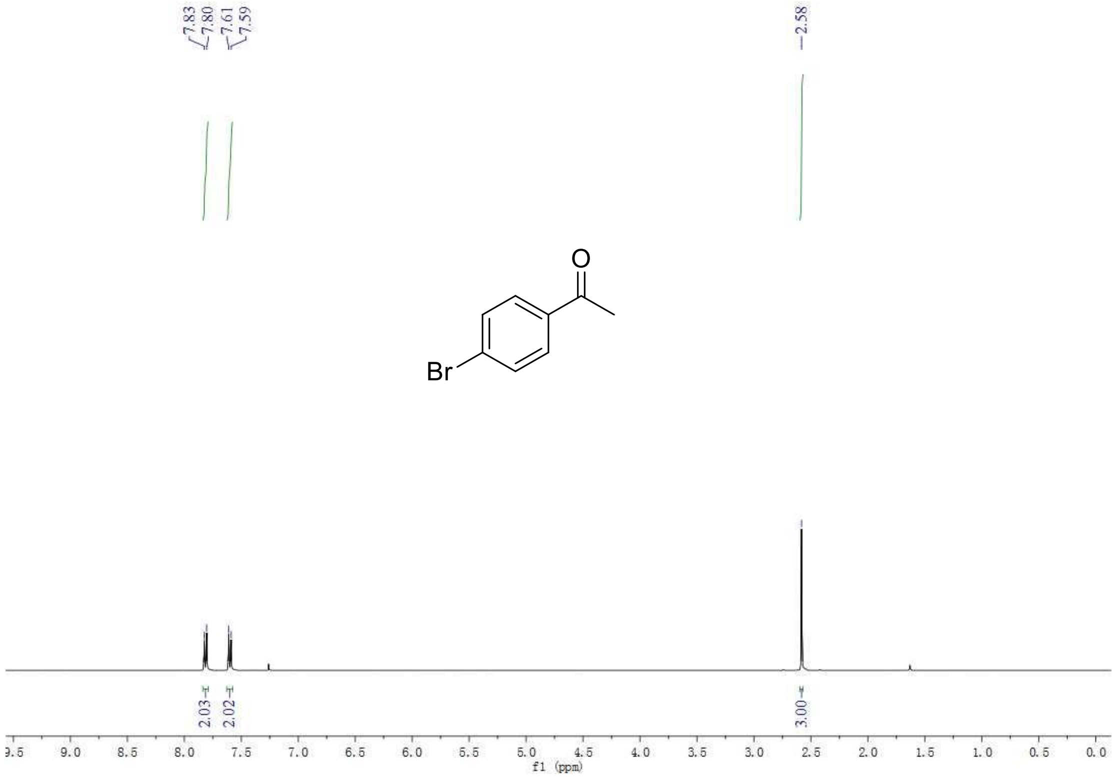

^{13}C{1H} NMR spectrum of 1-(4-bromophenyl)ethanone (4e, 101 MHz, $CDCl_3$)

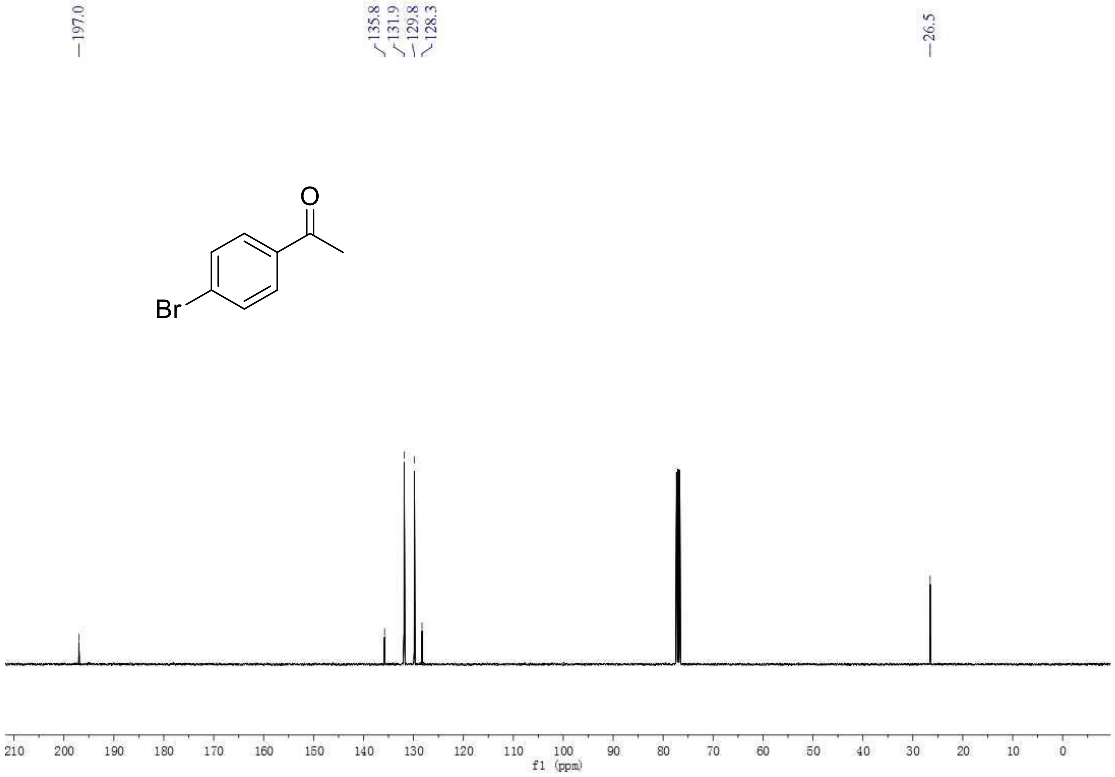

^{1}H NMR spectrum of benzophenone (4f, 400 MHz, CDCl$_3$)

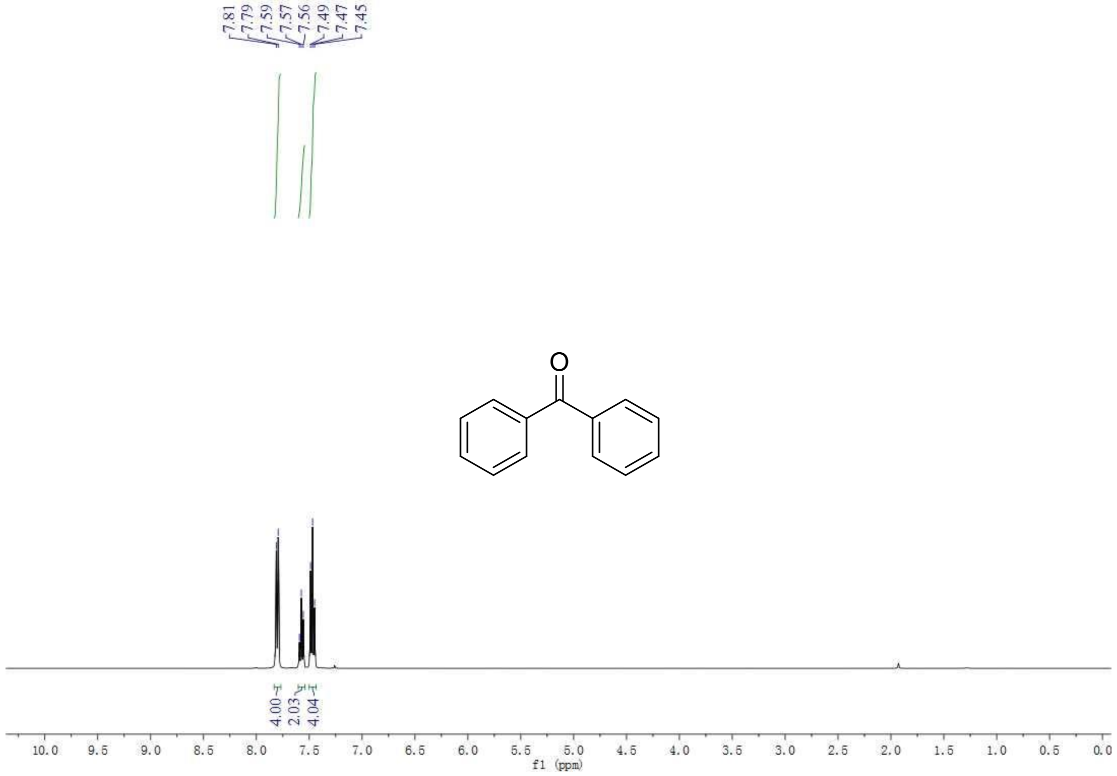

^{13}C{1H} NMR spectrum of benzophenone (4f, 101 MHz, CDCl$_3$)

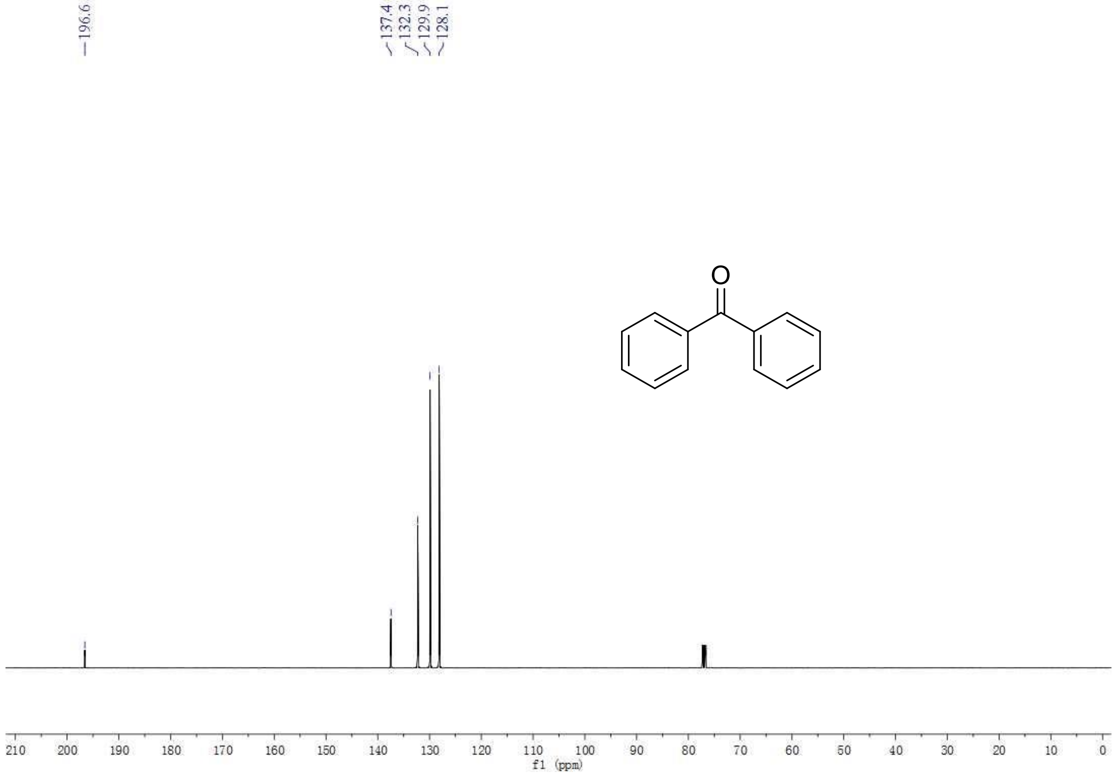

^{1}H NMR spectrum of phenyl(p-tolyl)methanone (4g, 400 MHz, CDCl$_3$)

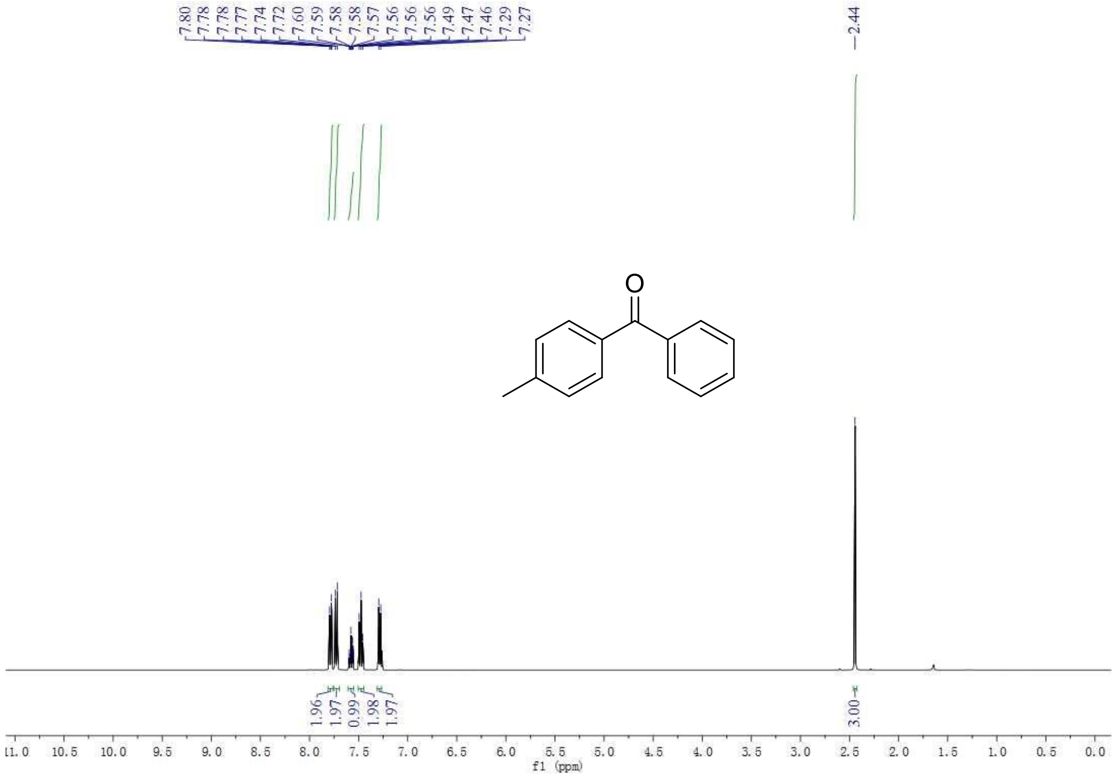

^{13}C{1H} NMR spectrum of phenyl(p-tolyl)methanone (4g, 101 MHz, CDCl$_3$)

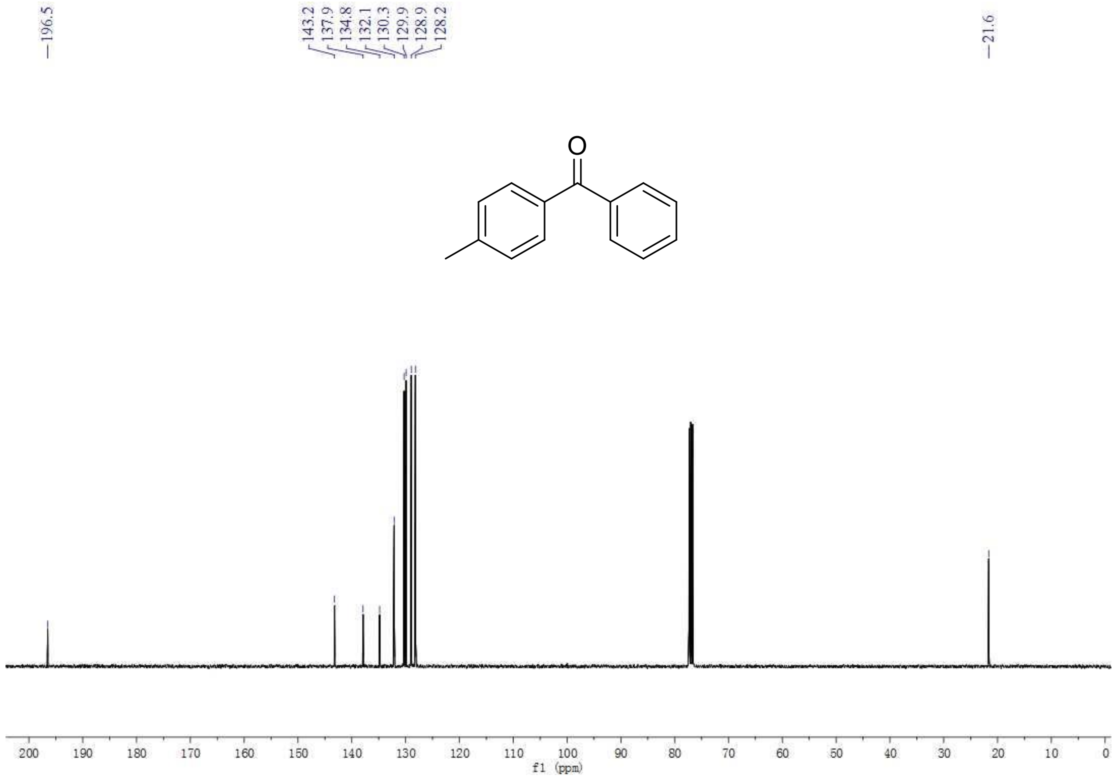

^{1}H NMR spectrum of (4-fluorophenyl)(phenyl)methanone (4h, 400 MHz, $CDCl_3$)

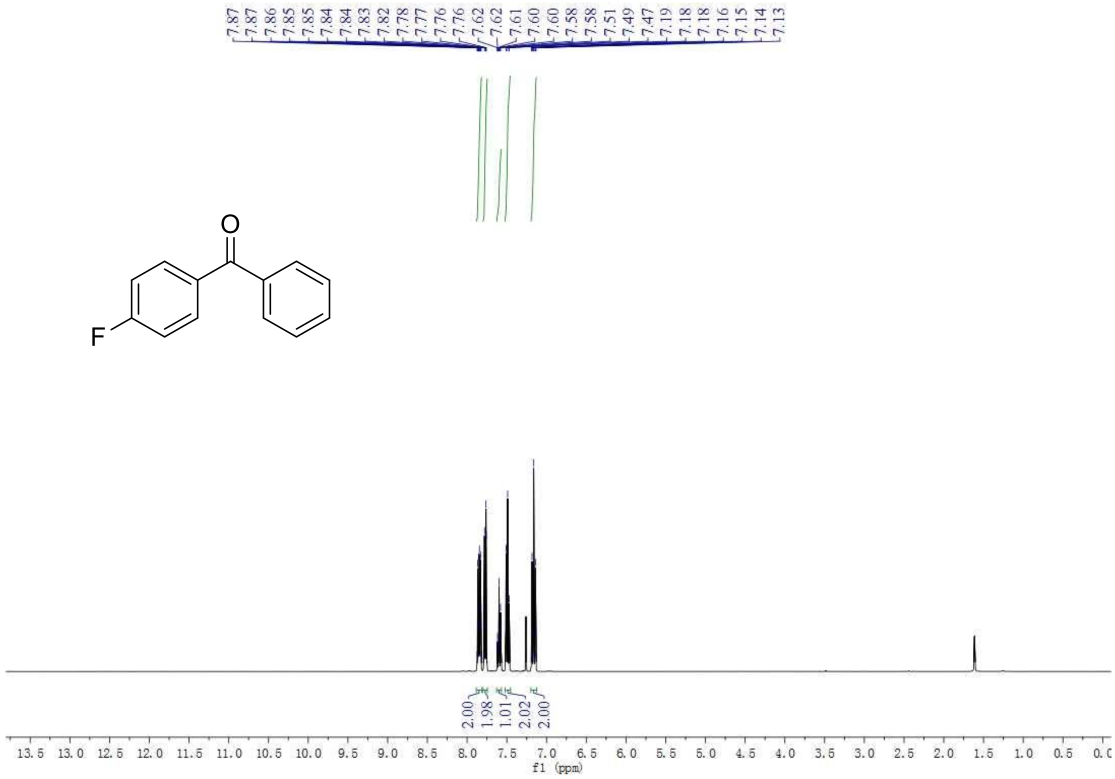

^{13}C{1H} NMR spectrum of (4-fluorophenyl)(phenyl)methanone (4h, 101 MHz, $CDCl_3$)

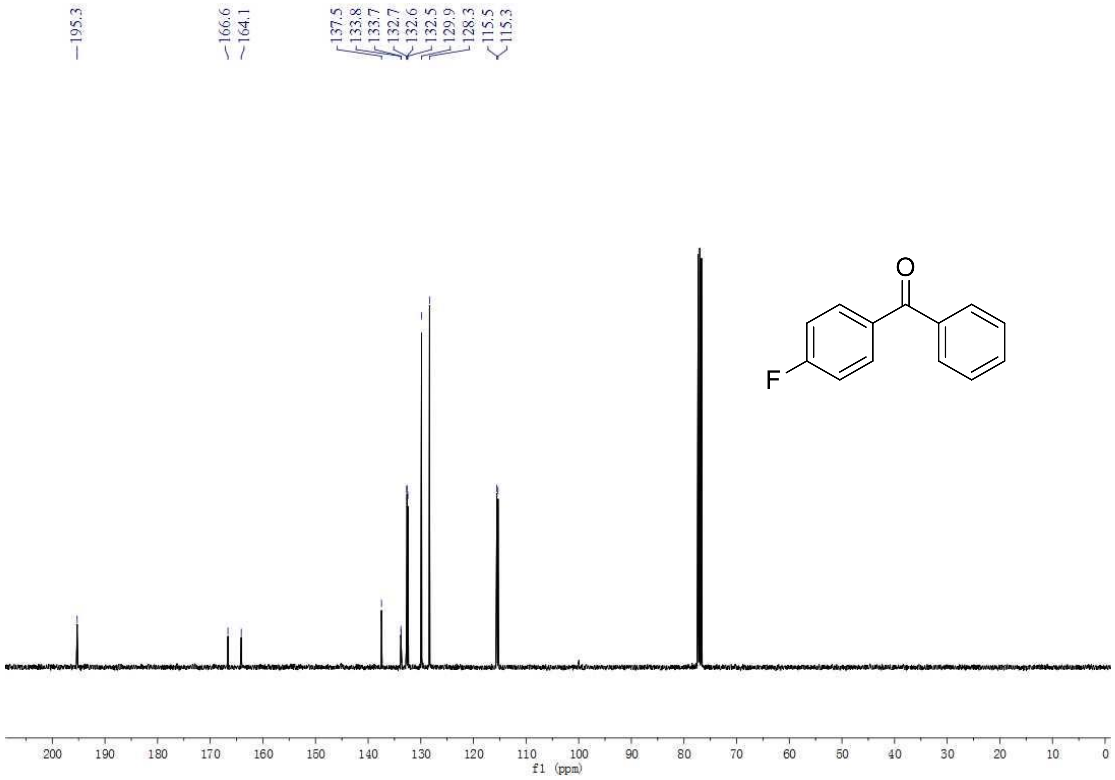

^{19}F NMR spectrum of (4-fluorophenyl)(phenyl)methanone (4h, 376 MHz, $CDCl_3$)

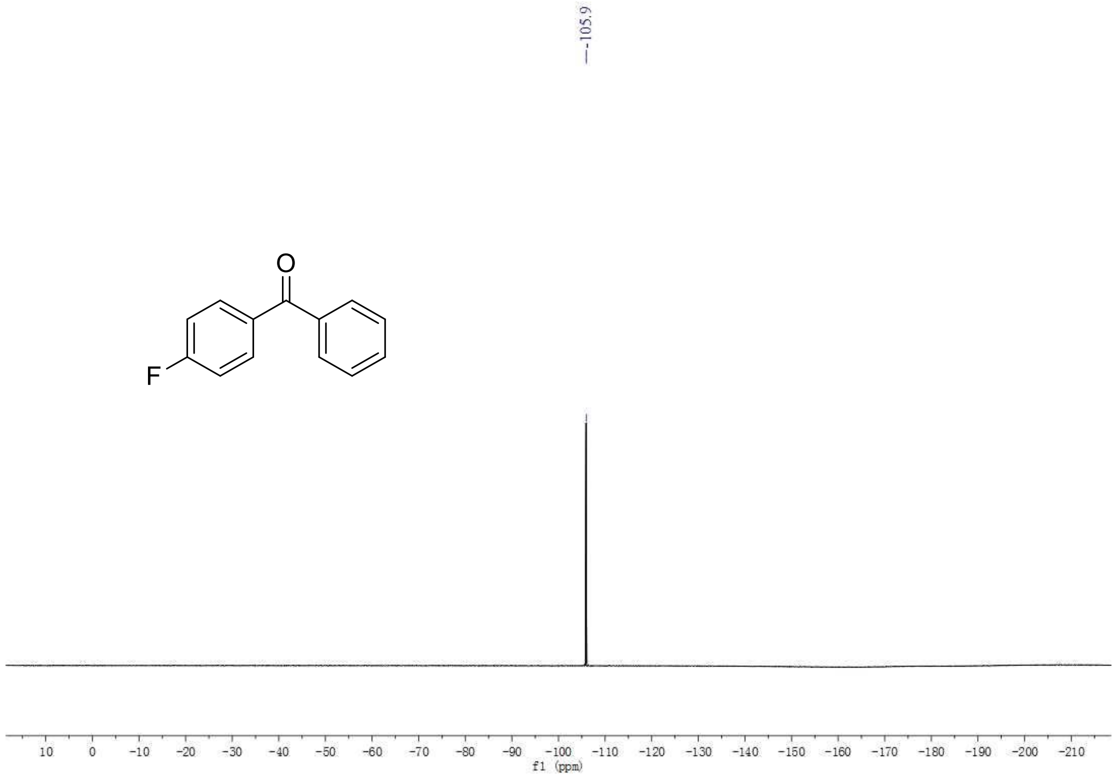

^{1}H NMR spectrum of (4-chlorophenyl)(phenyl)methanone (4i, 400 MHz, $CDCl_3$)

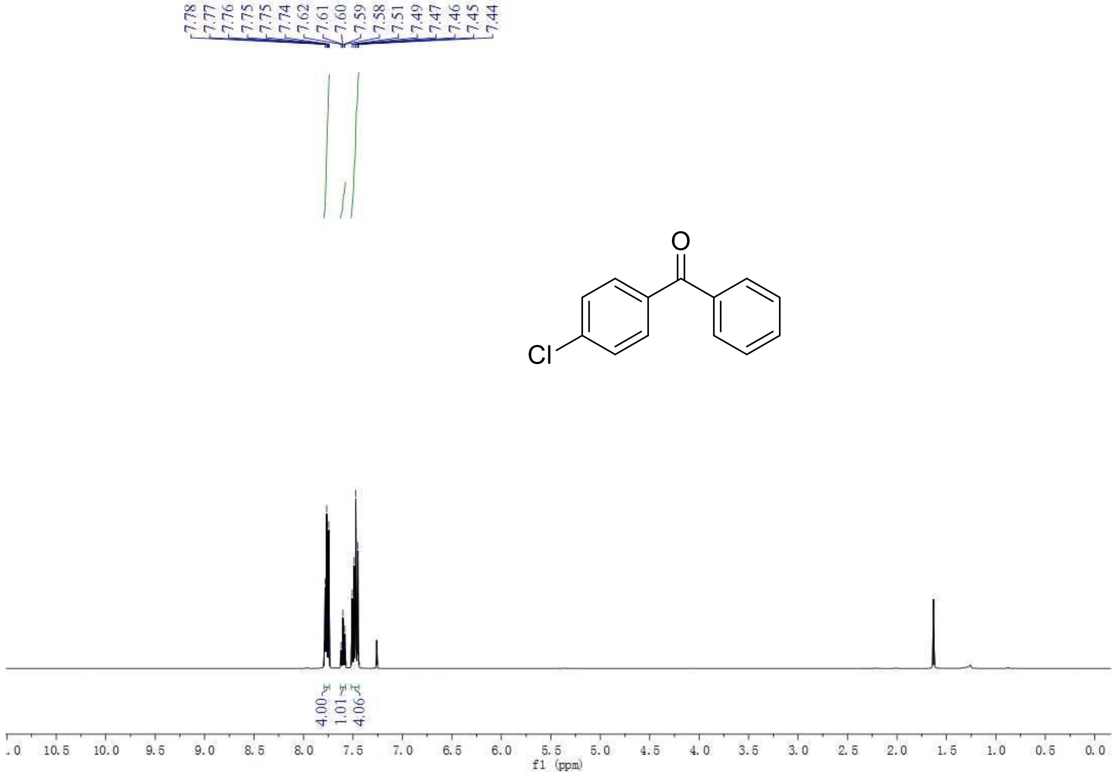

$^{13}C\{1H\}$ NMR spectrum of (4-chlorophenyl)(phenyl)methanone (4i, 101 MHz, $CDCl_3$)

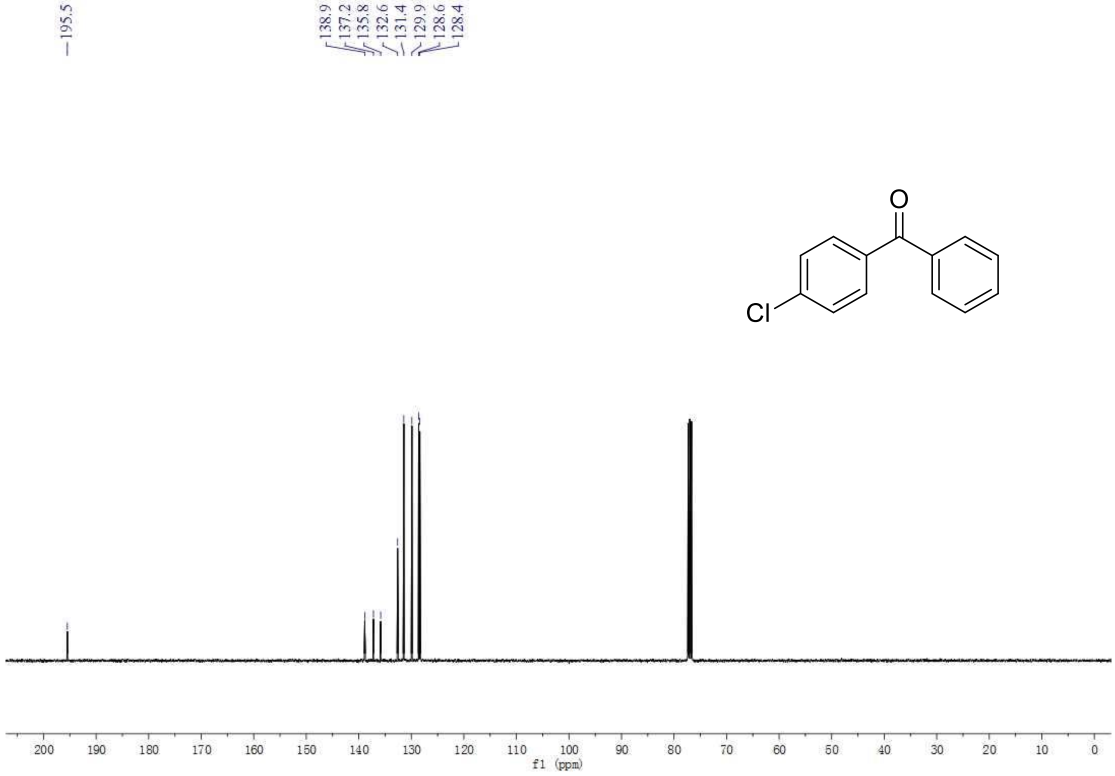

^{1}H NMR spectrum of (4-bromophenyl)(phenyl)methanone (4j, 400 MHz, $CDCl_3$)

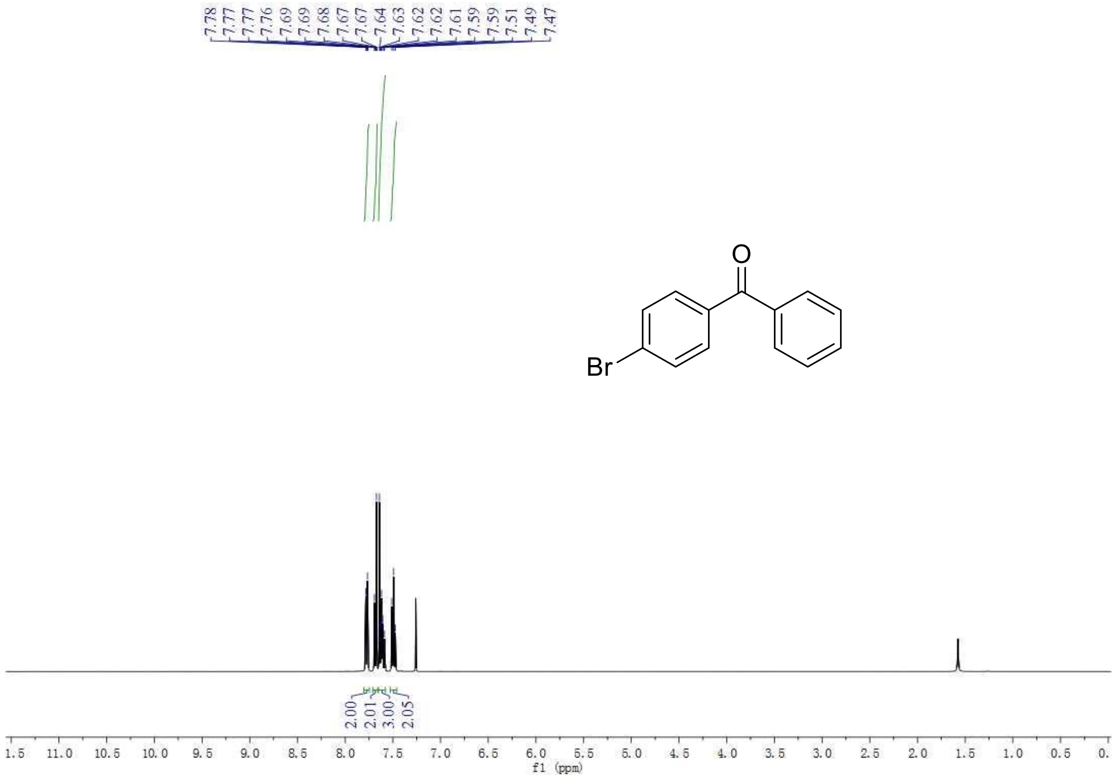

^{13}C{1H} NMR spectrum of (4-bromophenyl)(phenyl)methanone (4j, 101 MHz, CDCl3)

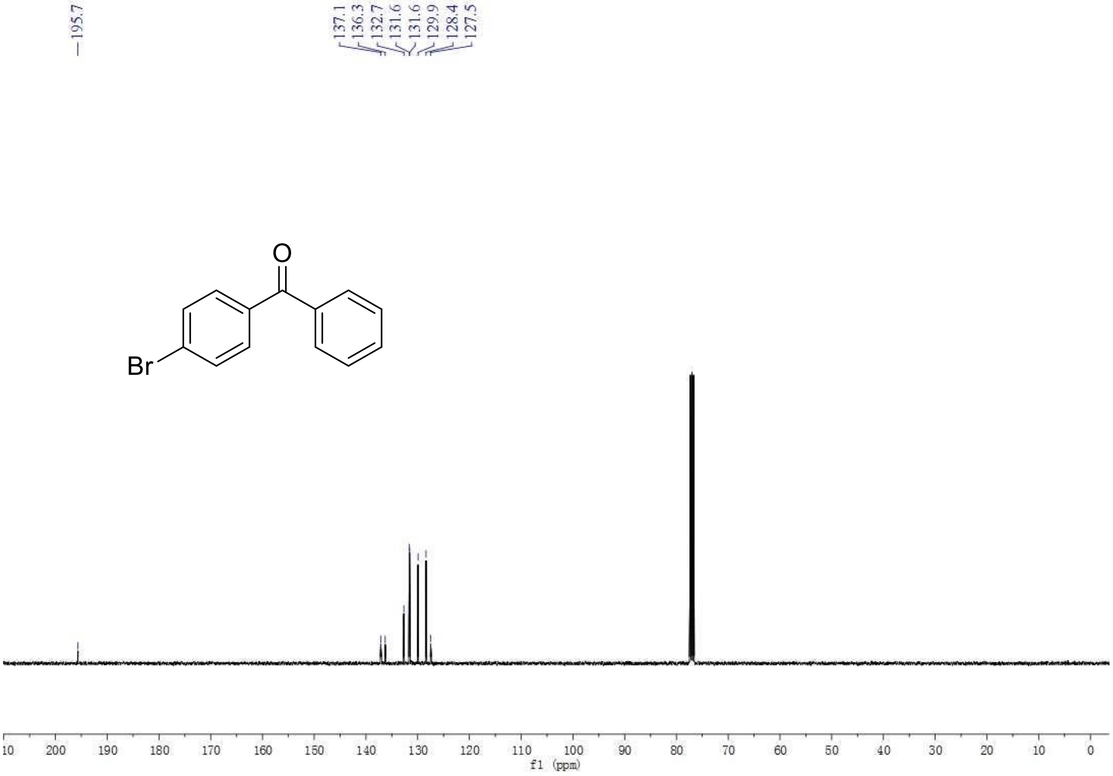

^{1}H NMR spectrum of 2-chloro-1-phenylethanone (4k, 400 MHz, CDCl3)

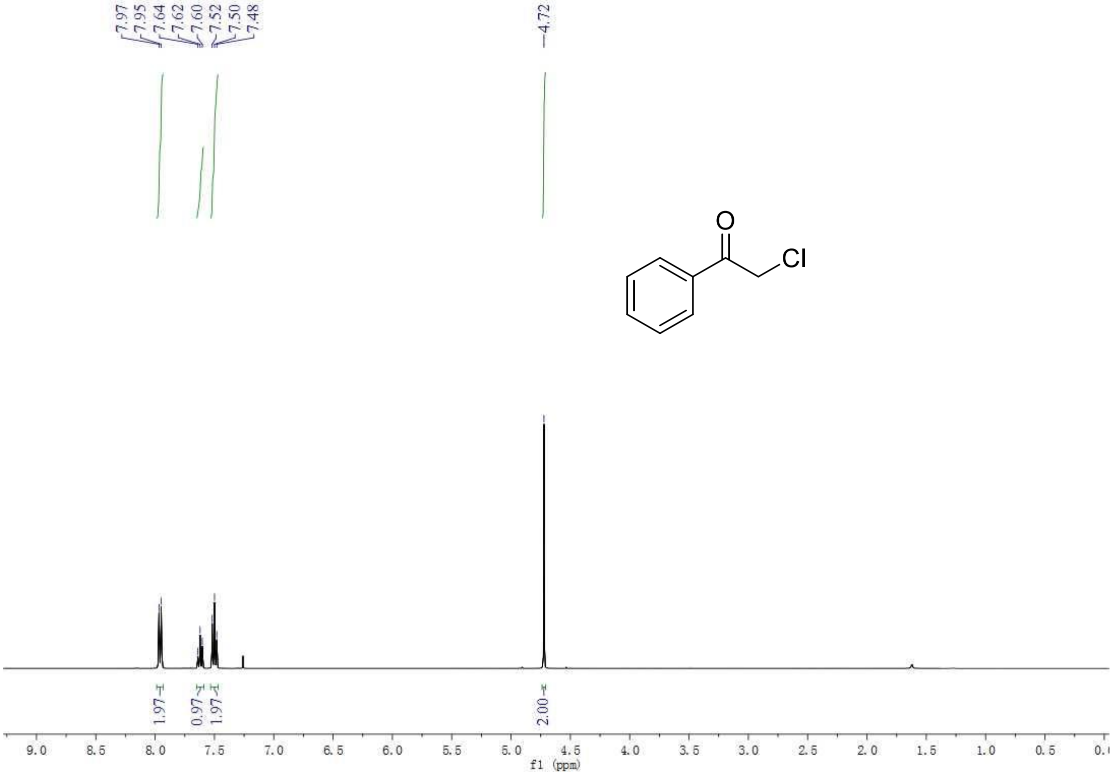

$^{13}C\{1H\}$ NMR spectrum of 2-chloro-1-phenylethanone (4k, 101 MHz, $CDCl_3$)

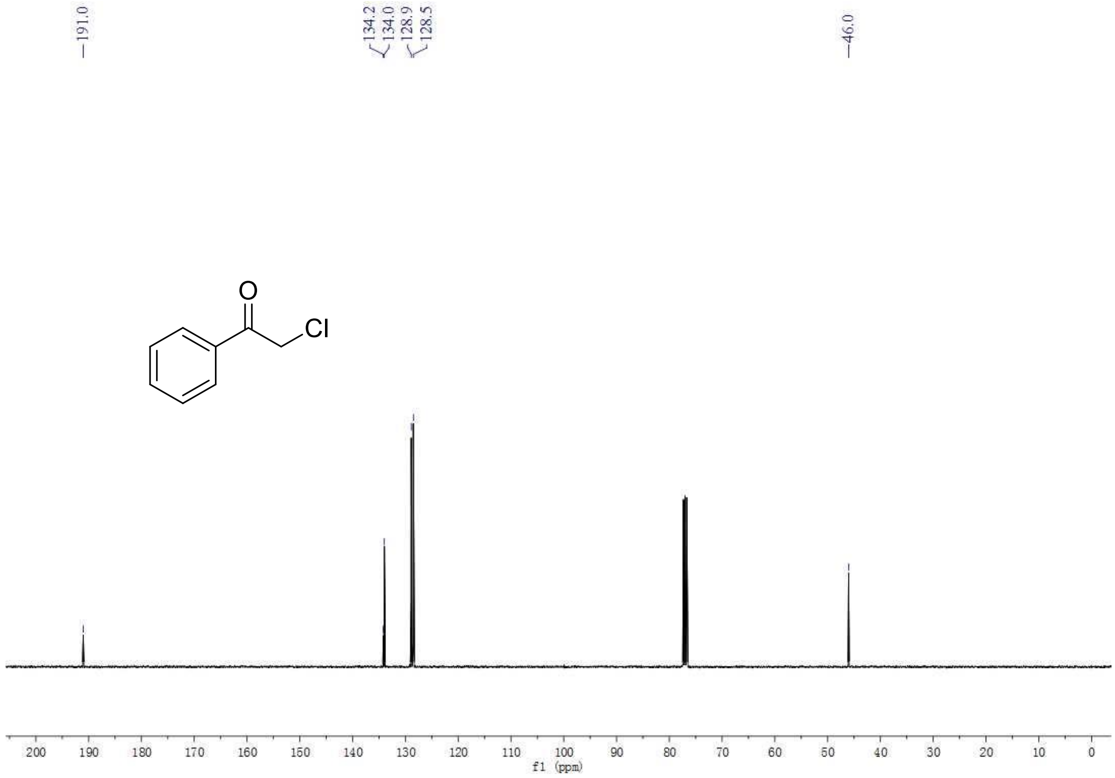

^1H NMR spectrum of 2-bromo-1-phenylethanone (4l, 400 MHz, $CDCl_3$)

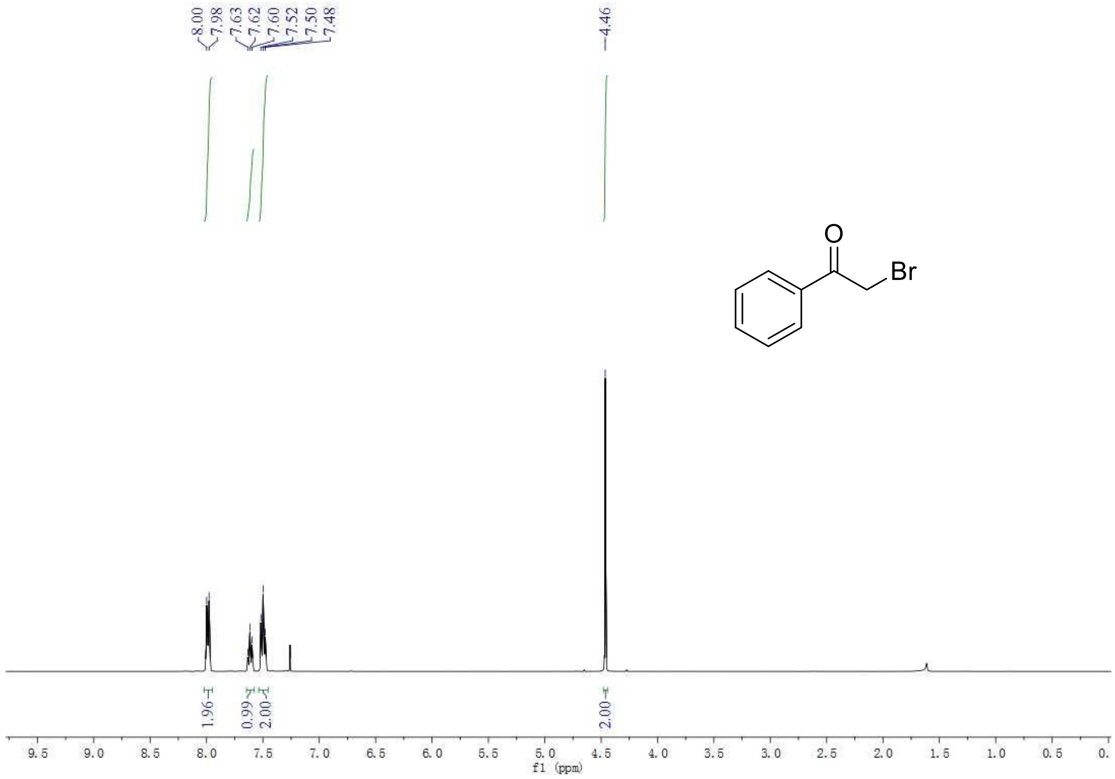

$^{13}C\{1H\}$ **NMR spectrum of 2-bromo-1-phenylethanone (4l, 101 MHz, $CDCl_3$)**

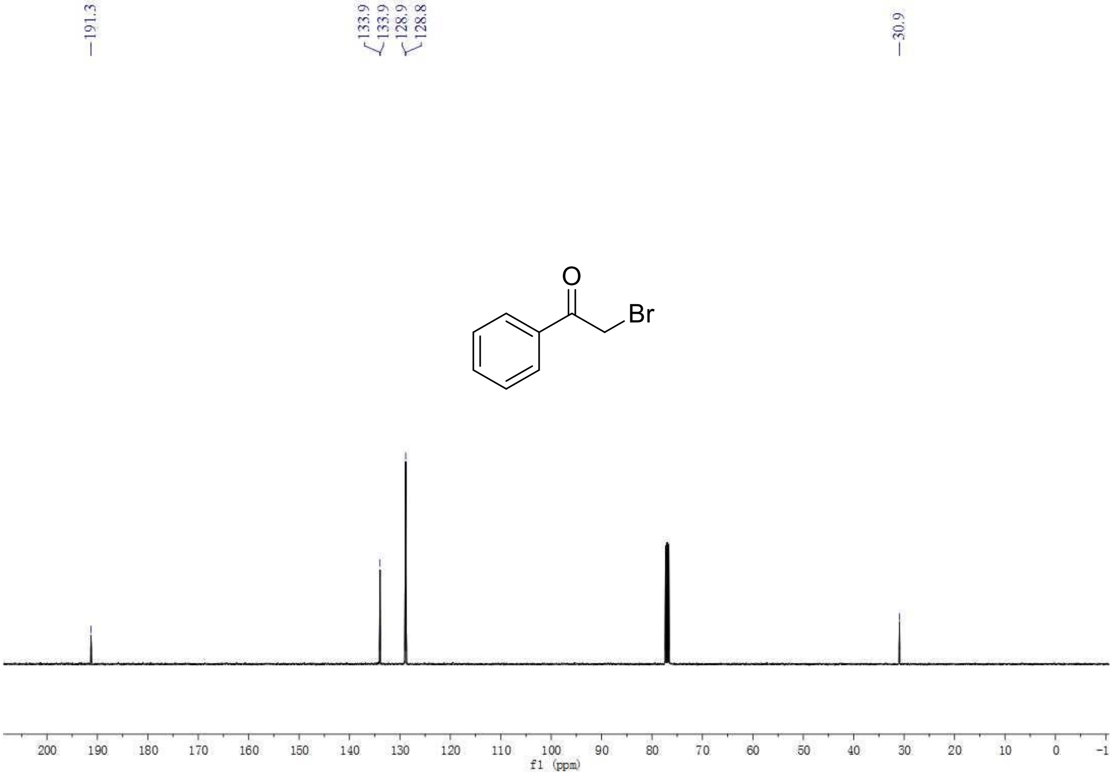

^{1}H **NMR spectrum of 3-chloro-1-phenylpropan-1-one (4m, 400 MHz, $CDCl_3$)**

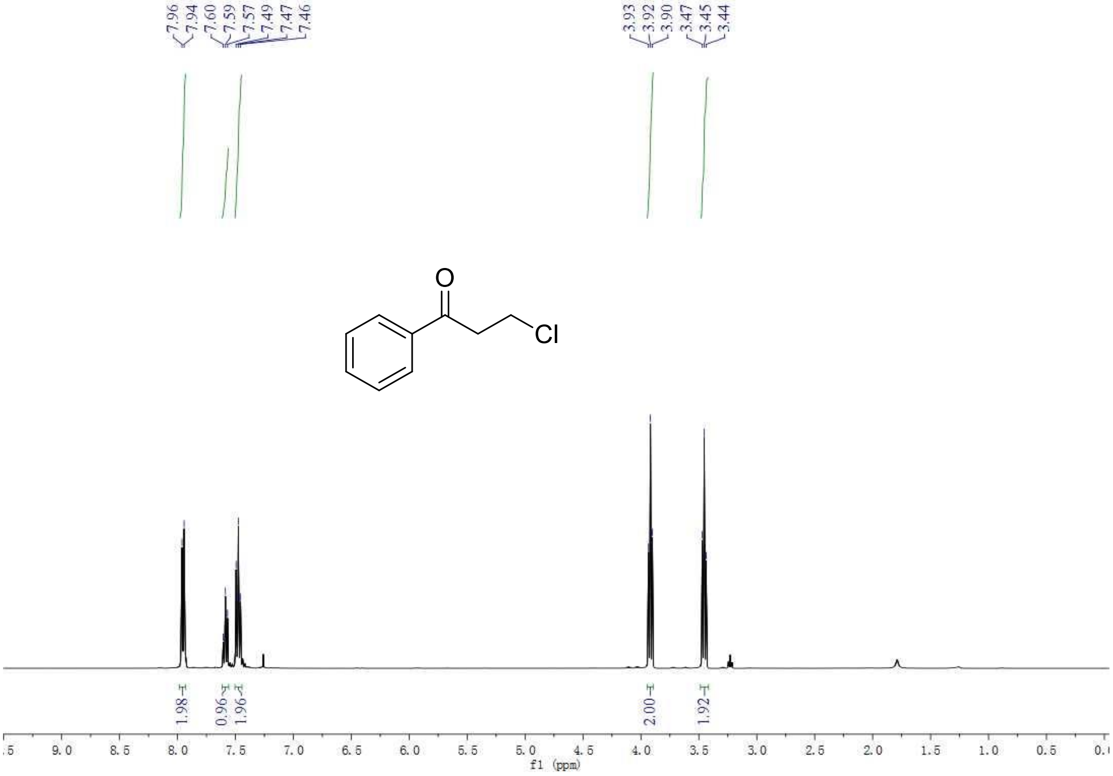

$^{13}C\{1H\}$ NMR spectrum of 3-chloro-1-phenylpropan-1-one (4m, 101 MHz, $CDCl_3$)

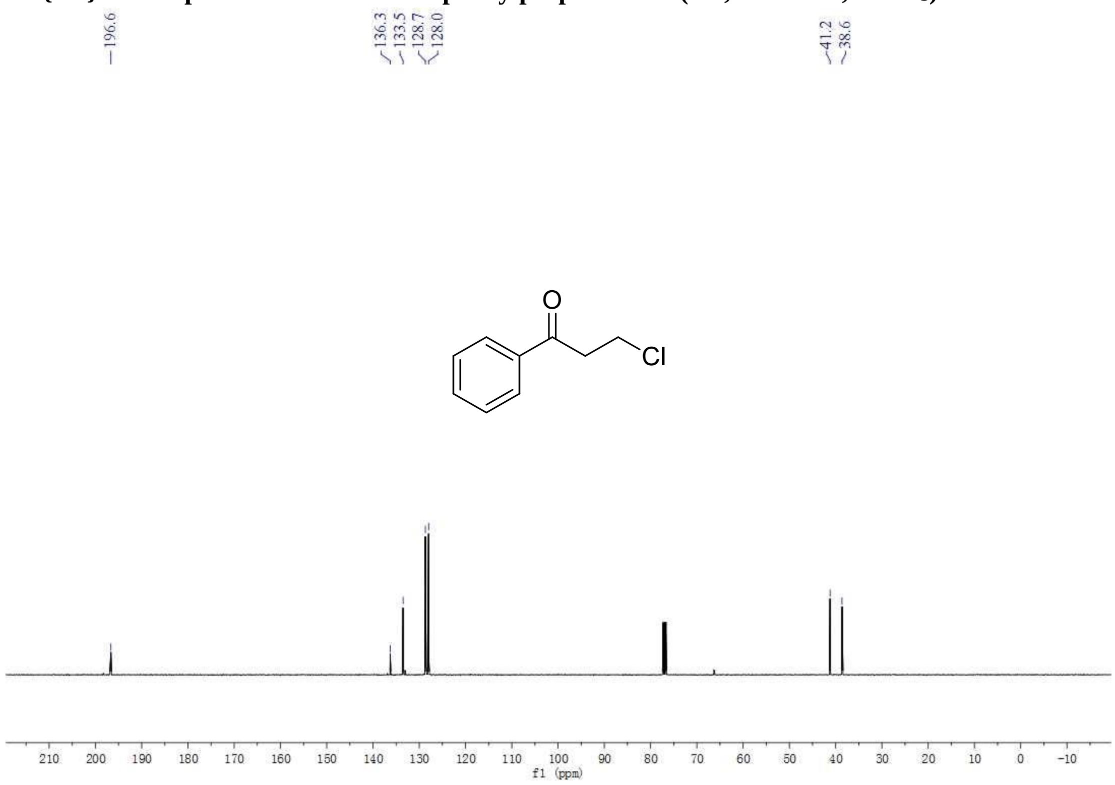

^{1}H NMR spectrum of benzoyl cyanide (4n, 400 MHz, $CDCl_3$)

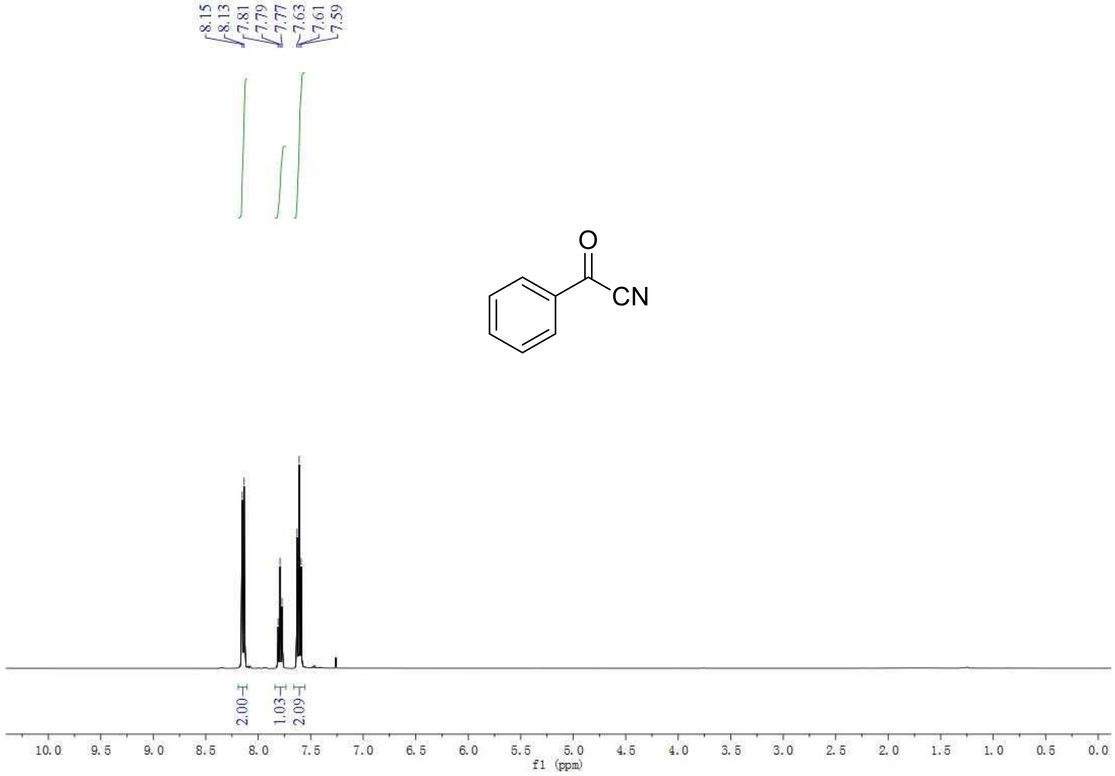

^{13}C{1H} NMR spectrum of benzoyl cyanide (4n, 101 MHz, $CDCl_3$)

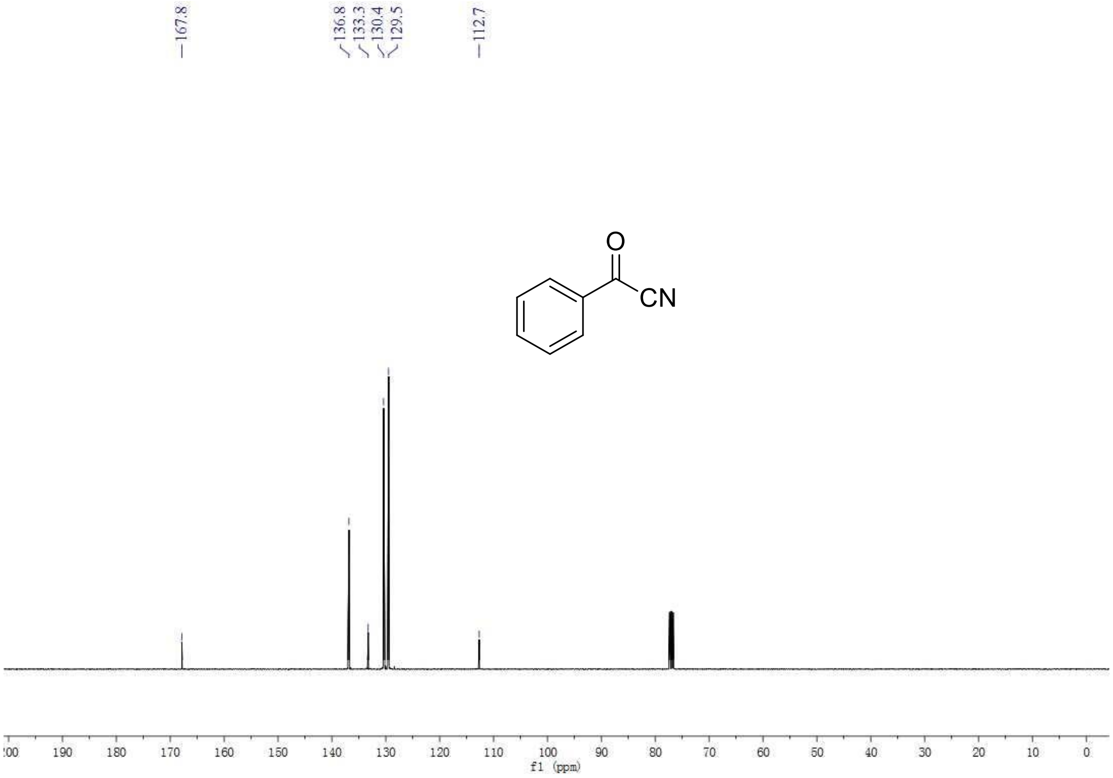

^{1}H NMR spectrum of 9*H*-fluoren-9-one (4o, 400 MHz, $CDCl_3$)

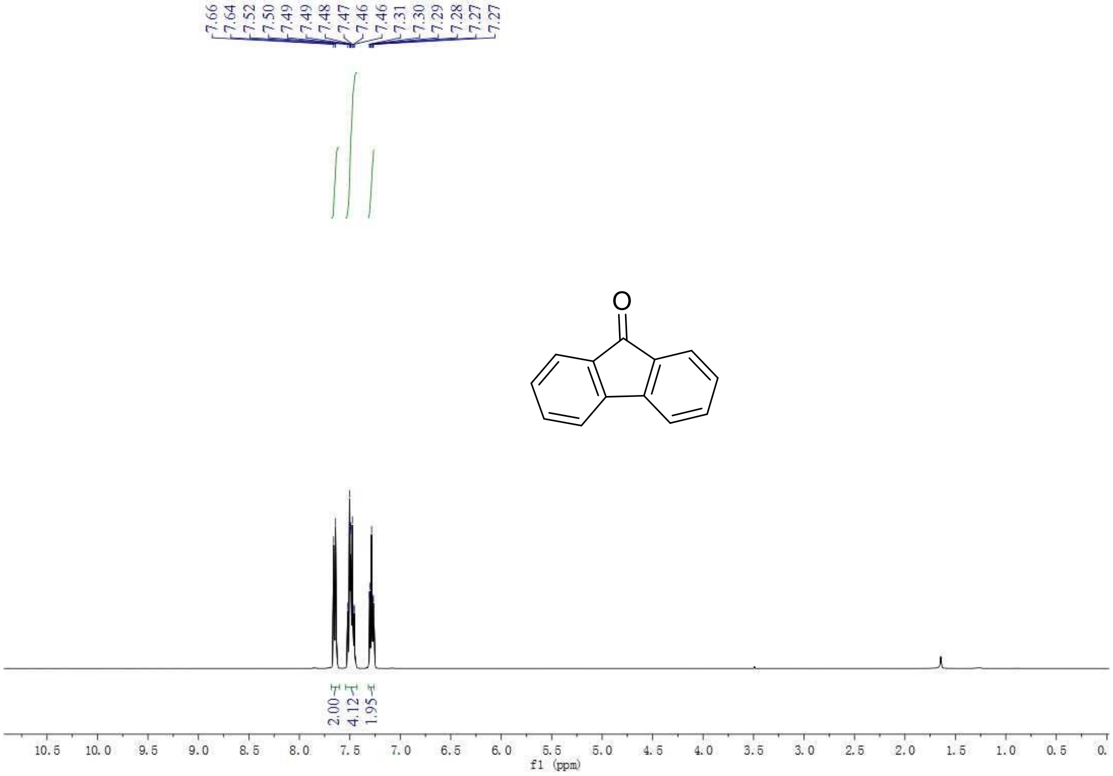

^{13}C{1H} NMR spectrum of 9*H*-fluoren-9-one (4o, 101 MHz, $CDCl_3$)

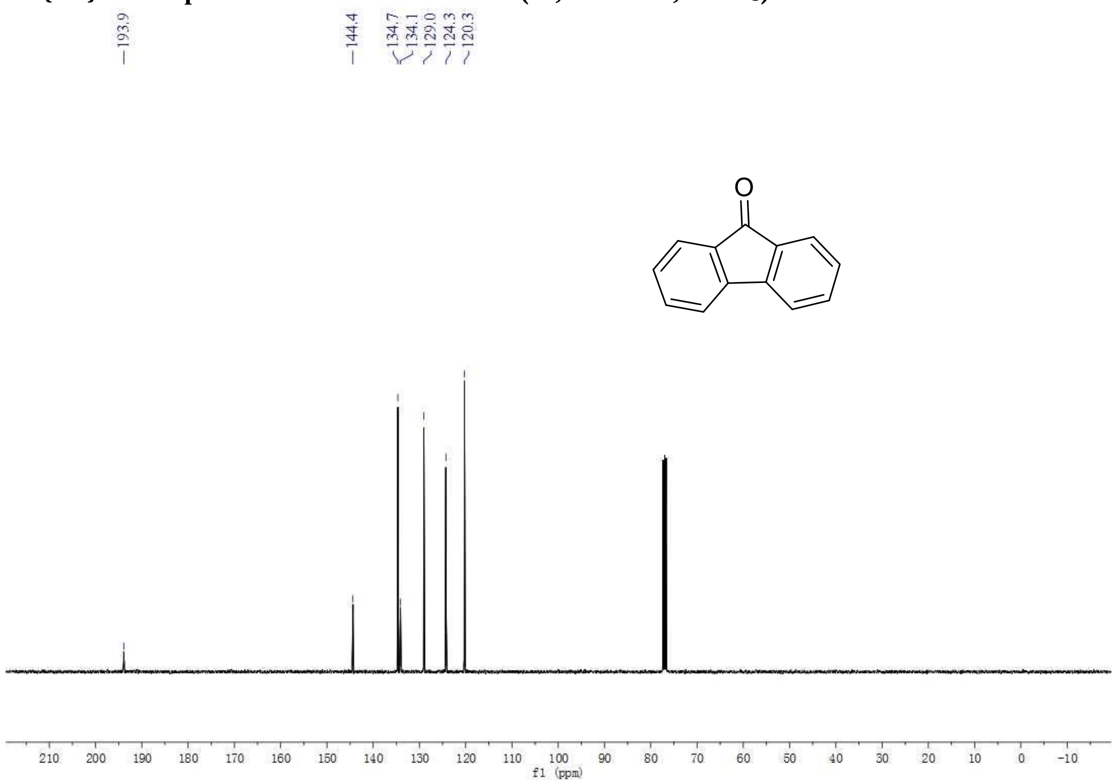

^{1}H NMR spectrum of 9*H*-xanthen-9-one (4p, 400 MHz, $CDCl_3$)

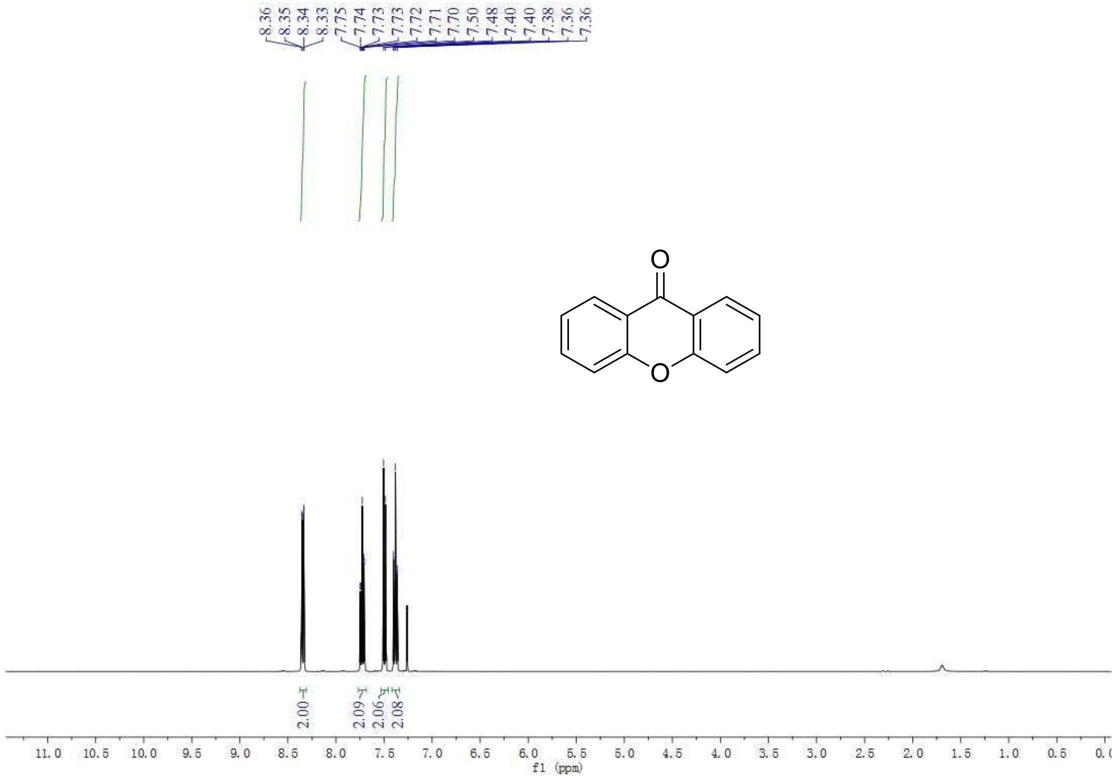

$^{13}C\{1H\}$ NMR spectrum of 9*H*-xanthen-9-one (4p, 101 MHz, CDCl$_3$)

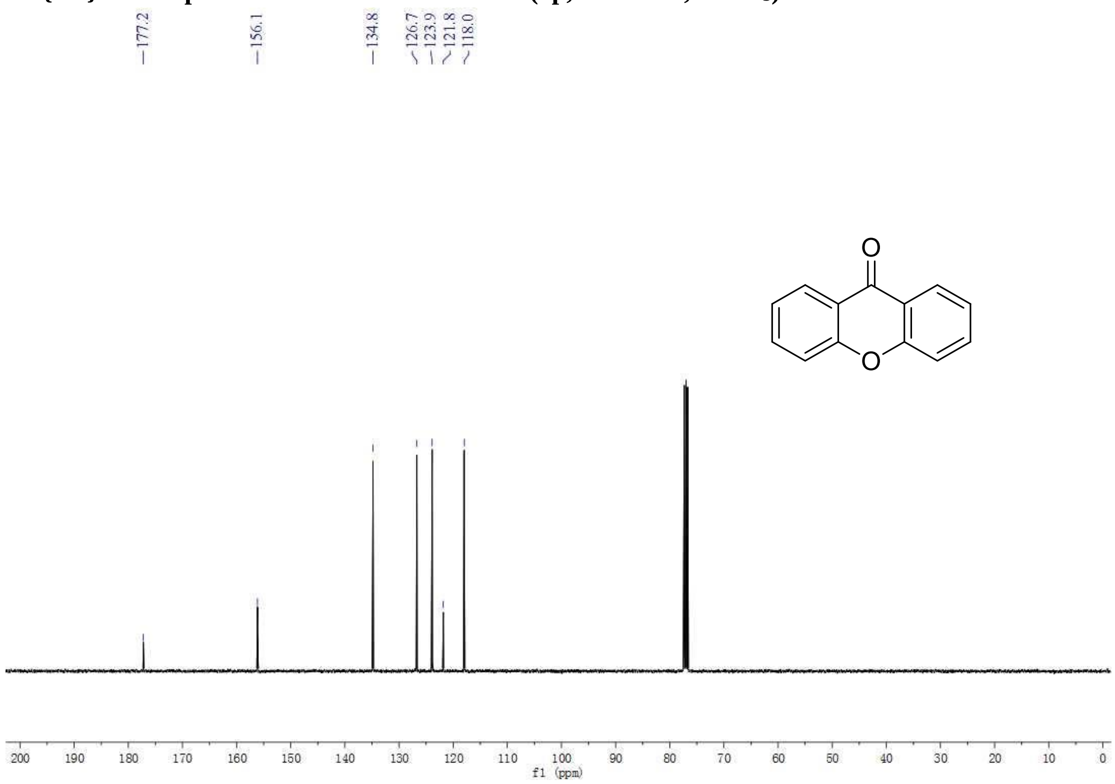

^{1}H NMR spectrum of 9*H*-thioxanthen-9-one (4q, 400 MHz, CDCl$_3$)

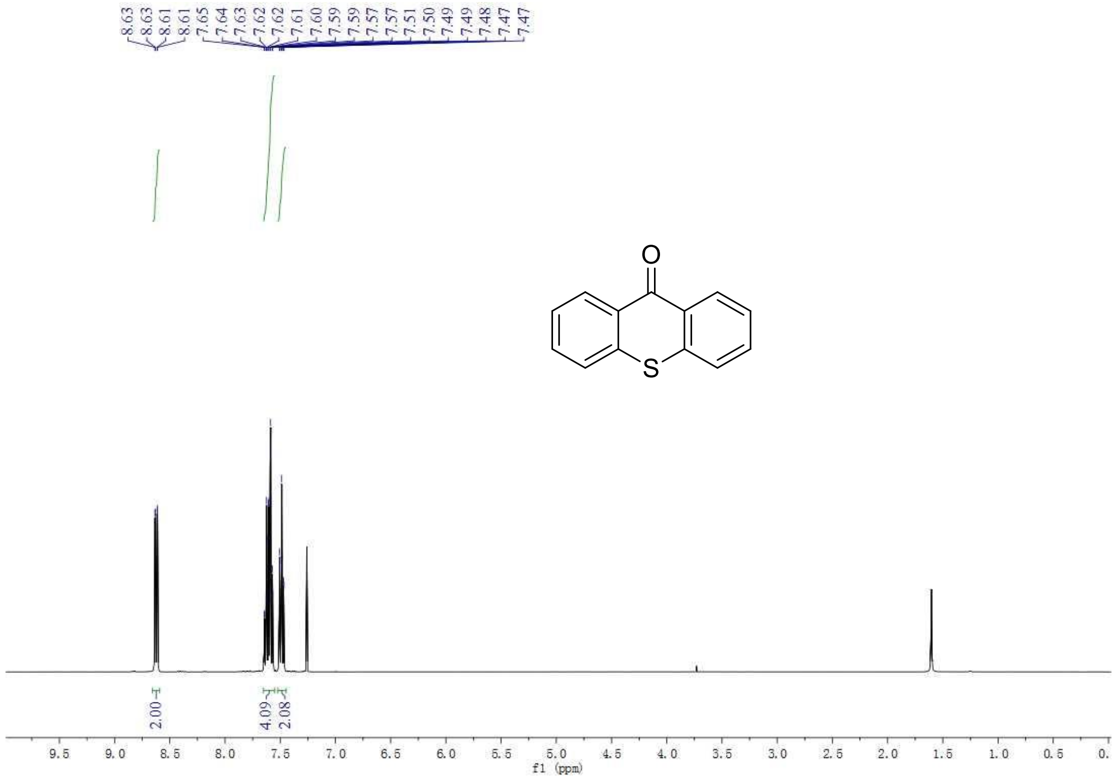

$^{13}C\{^1H\}$ NMR spectrum of 9*H*-thioxanthen-9-one (4q, 101 MHz, $CDCl_3$)

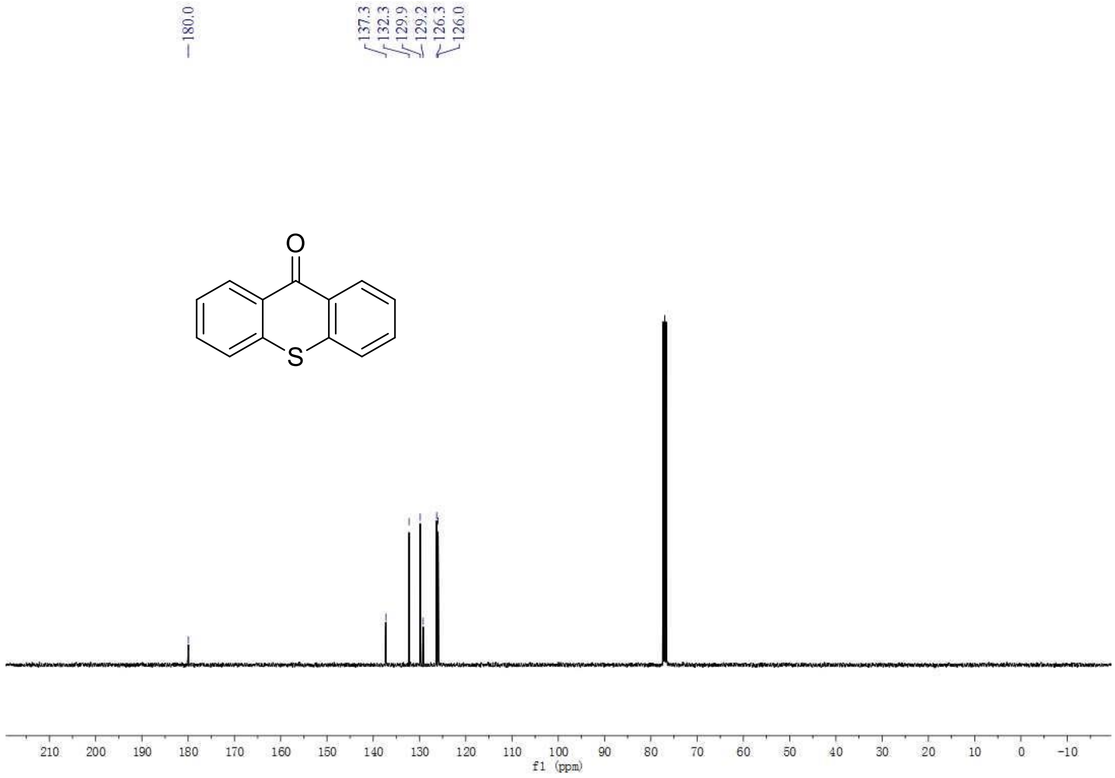

^1H NMR spectrum of methyl 2-(1-(4-acetylbenzoyl)-5-methoxy-2-methyl-1H-indol-3-yl)acetate (4r, 400 MHz, $CDCl_3$)

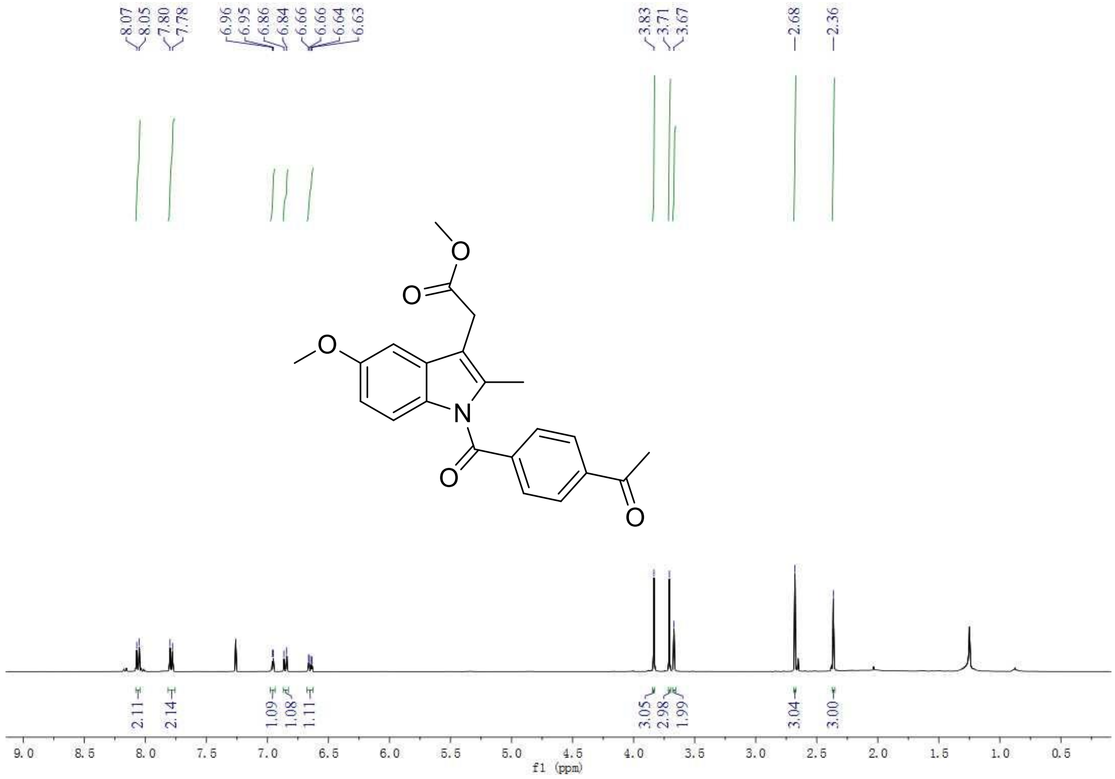

^{13}C{1H} NMR spectrum of methyl 2-(1-(4-acetylbenzoyl)-5-methoxy-2-methyl-1H-indol-3-yl)acetate (4r, 101 MHz, $CDCl_3$)

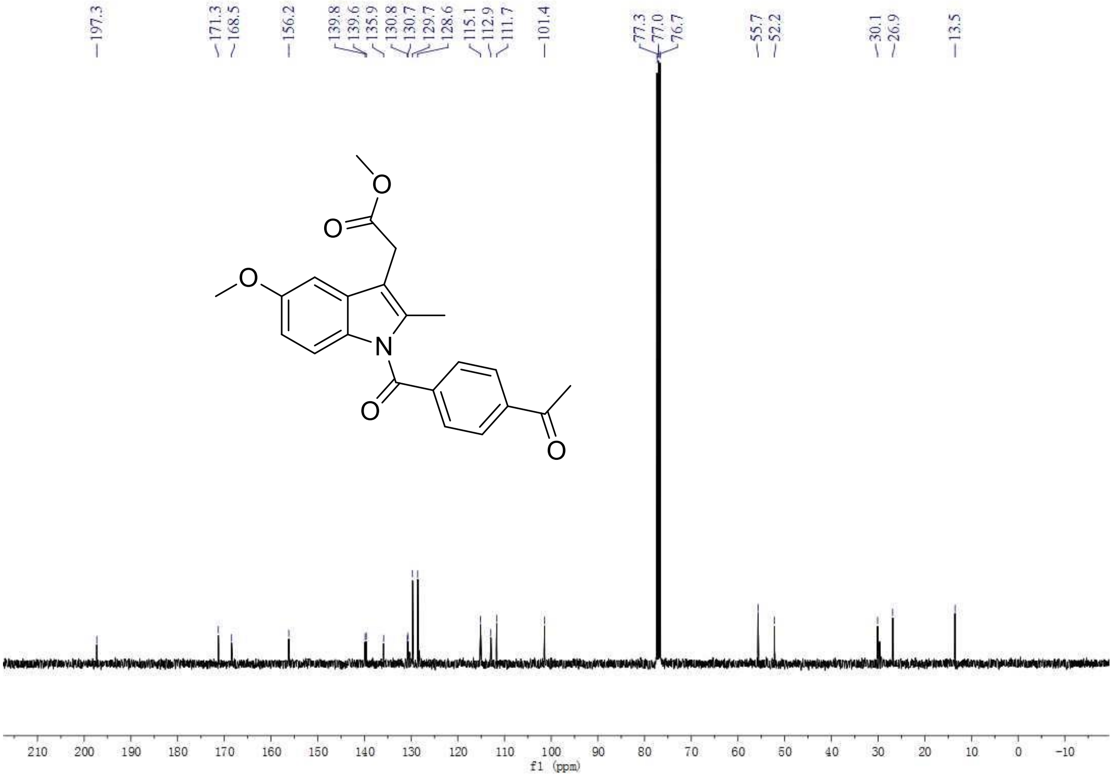

^{1}H NMR spectrum of isopropyl 2-(4-(4-acetylbenzoyl)phenoxy)-2-methylpropanoate (4s, 400 MHz, $CDCl_3$)

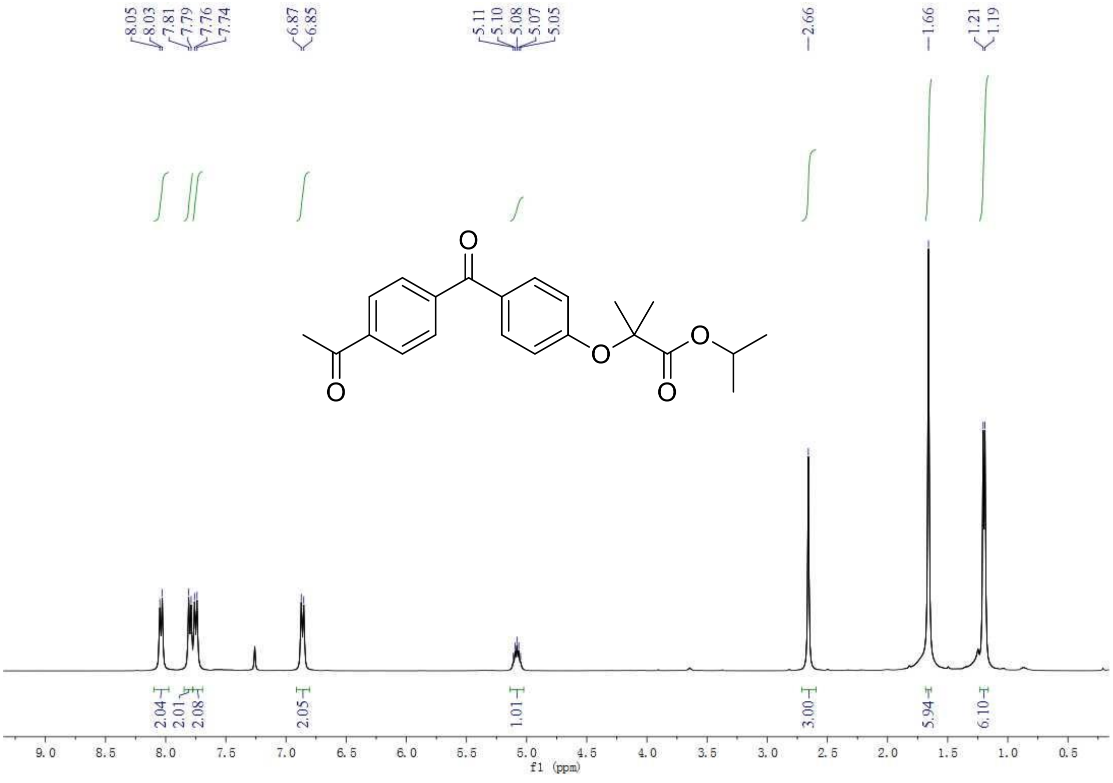

$^{13}C\{^1H\}$ NMR spectrum of isopropyl 2-(4-(4-acetylbenzoyl)phenoxy)-2-methylpropanoate (4s, 101 MHz, $CDCl_3$)

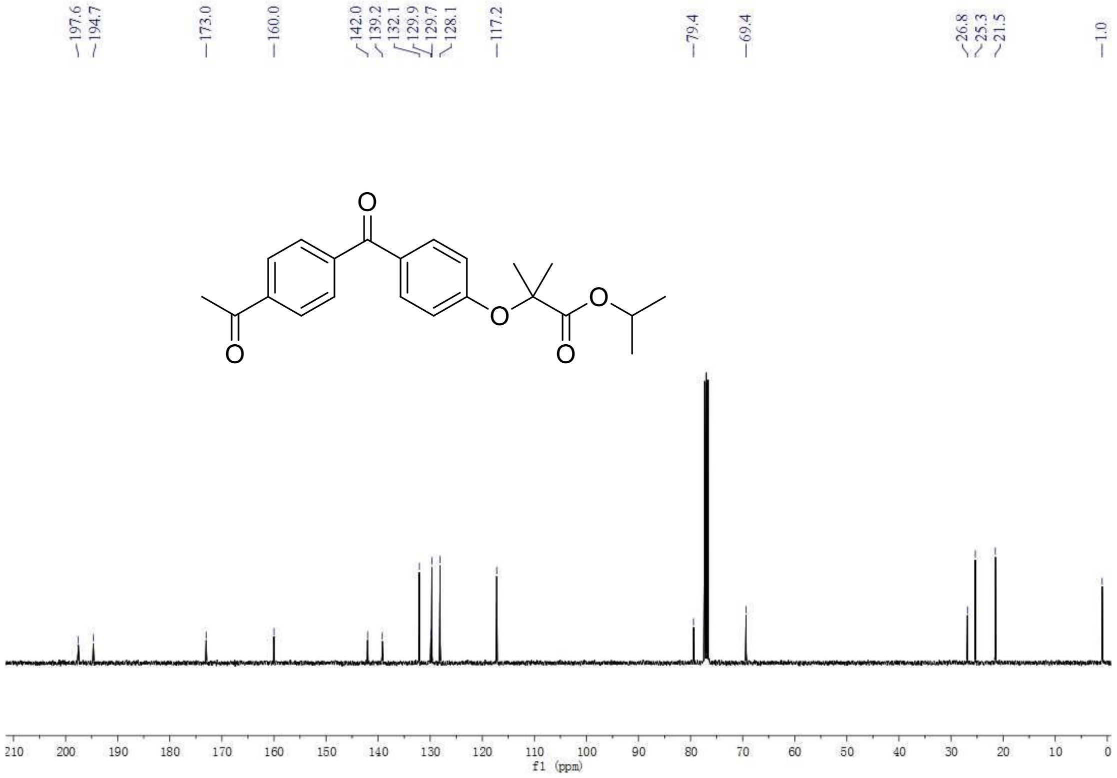

^1H NMR spectrum of benzohydrazide (5a, 400 MHz, DMSO-*d6*)

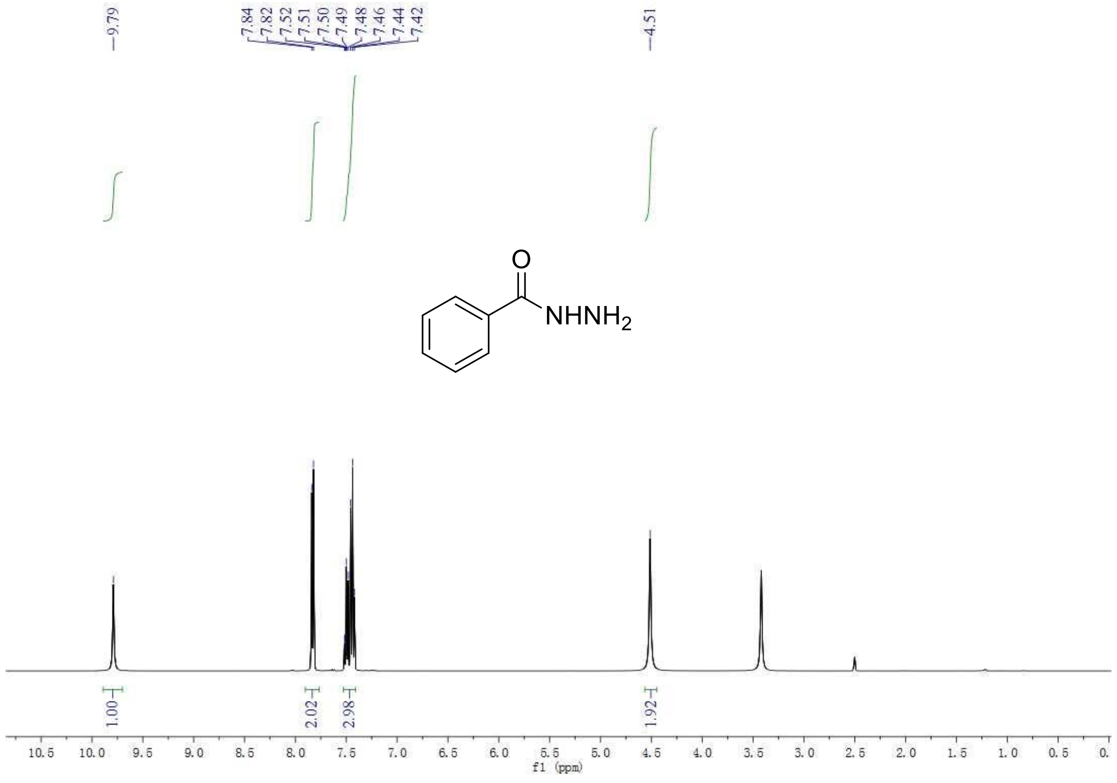

^{13}C{1H} NMR spectrum of benzohydrazide (5a, 101 MHz, DMSO-*d6*)

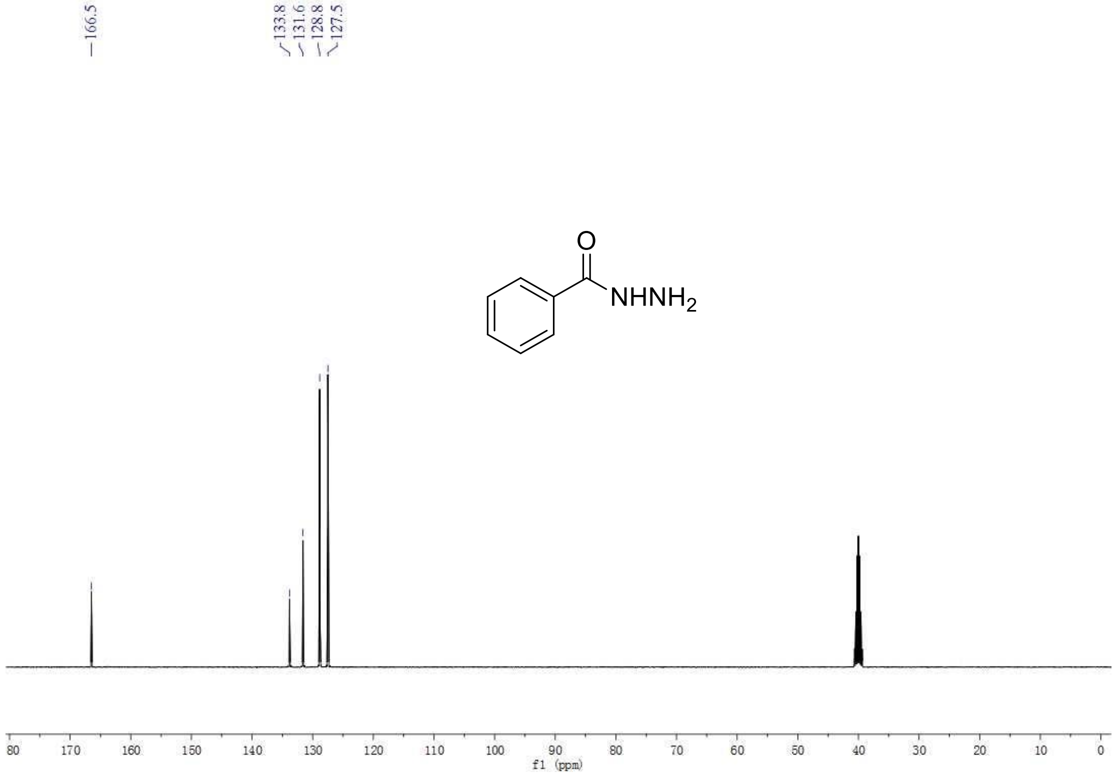

^{1}H NMR spectrum of phenyl benzoate (5b, 400 MHz, CDCl$_3$)

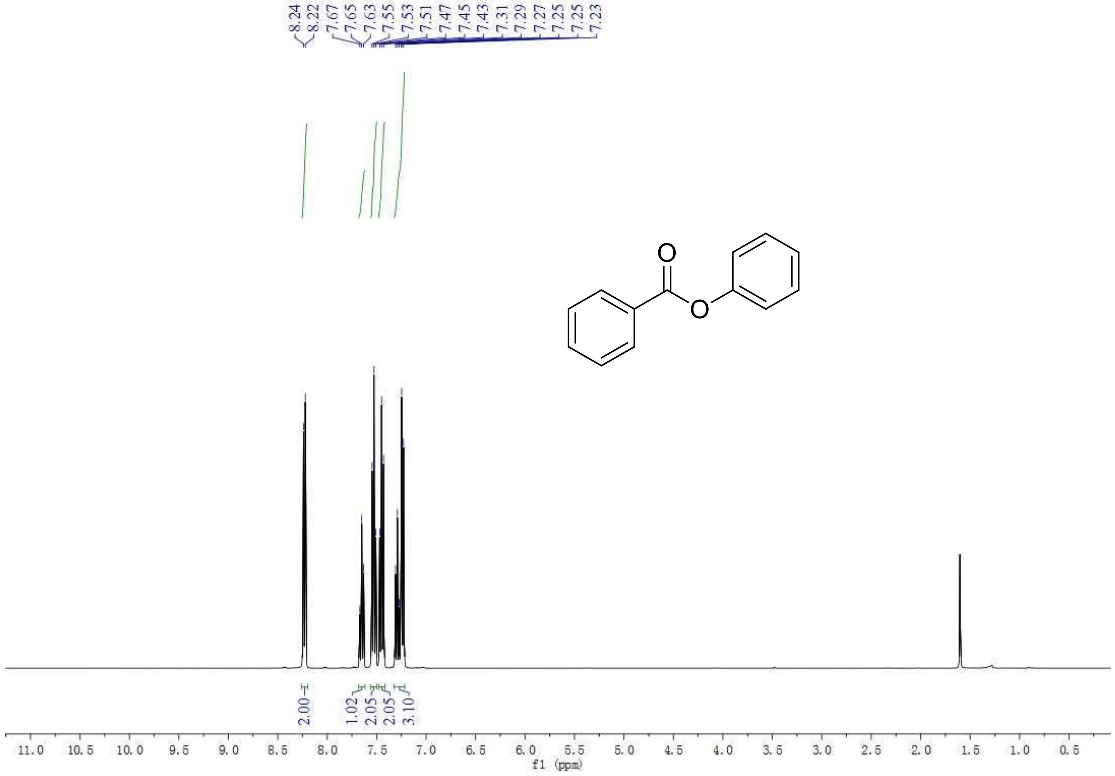

$^{13}C\{1H\}$ NMR spectrum of phenyl benzoate (5b, 101 MHz, $CDCl_3$)

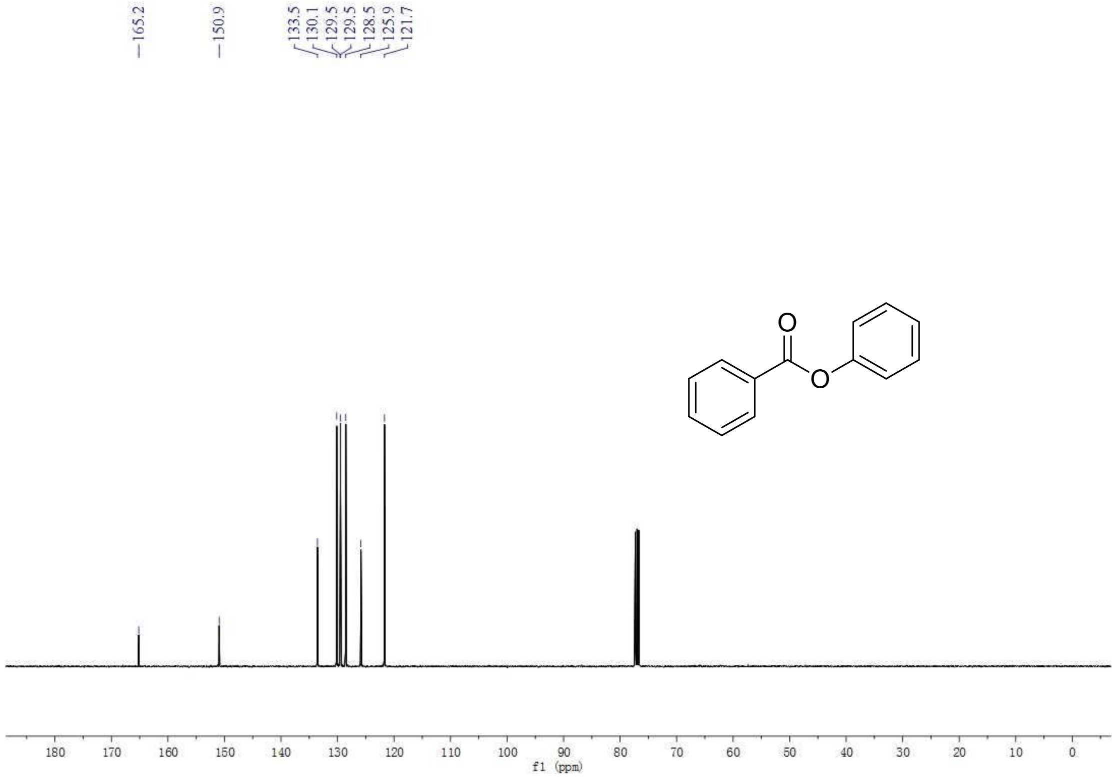

^{1}H NMR spectrum of acetophenone oxime (5c, 400 MHz, DMSO-*d6*)

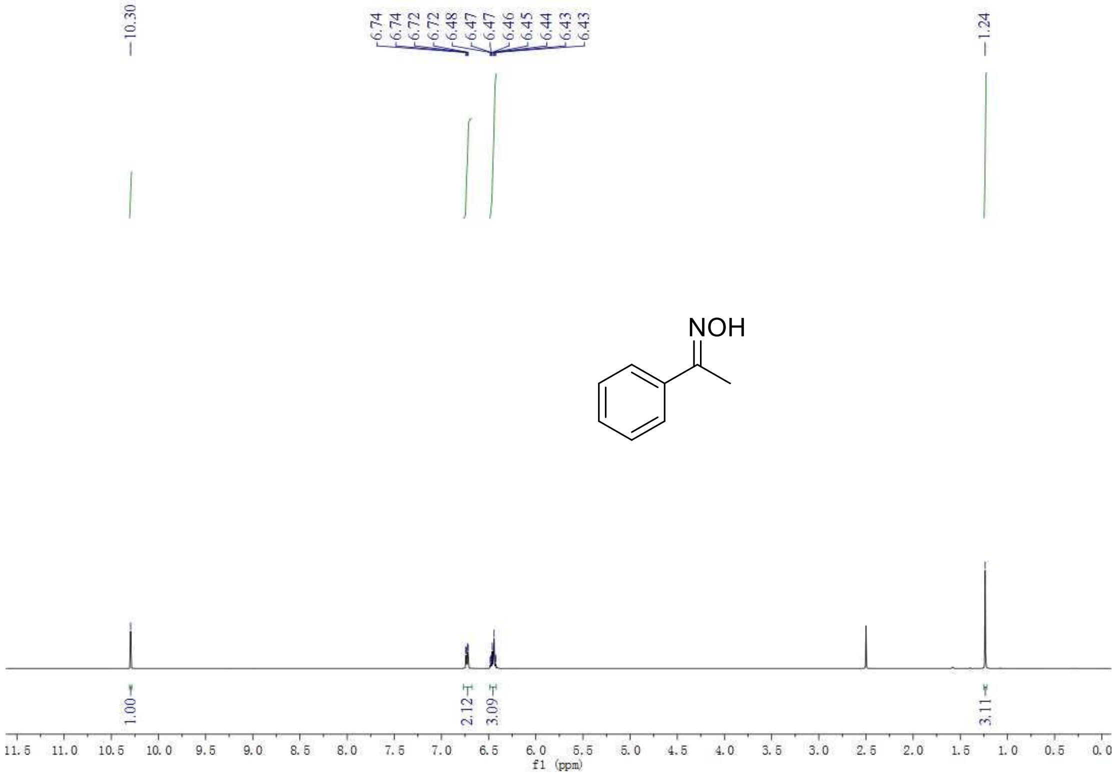

^{13}C{1H} NMR spectrum of acetophenone oxime (5c, 101 MHz, DMSO-*d6*)

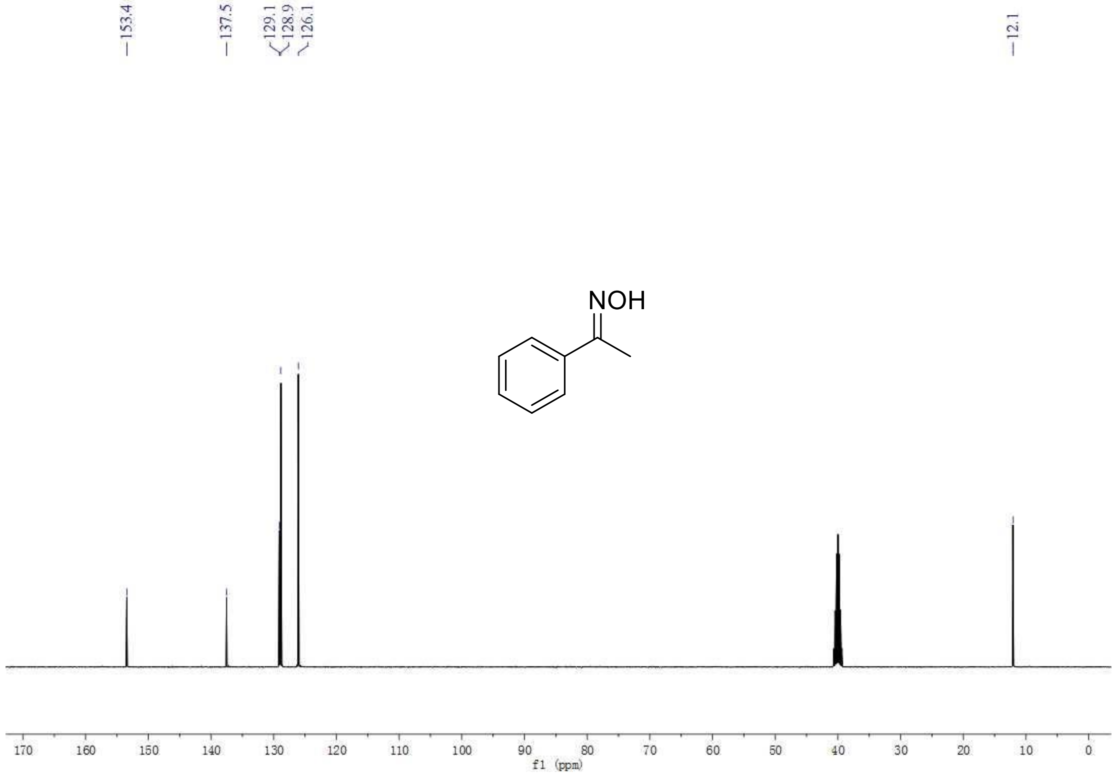

^{1}H NMR spectrum of (*E*)-chalcone (5d, 400 MHz, $CDCl_3$)

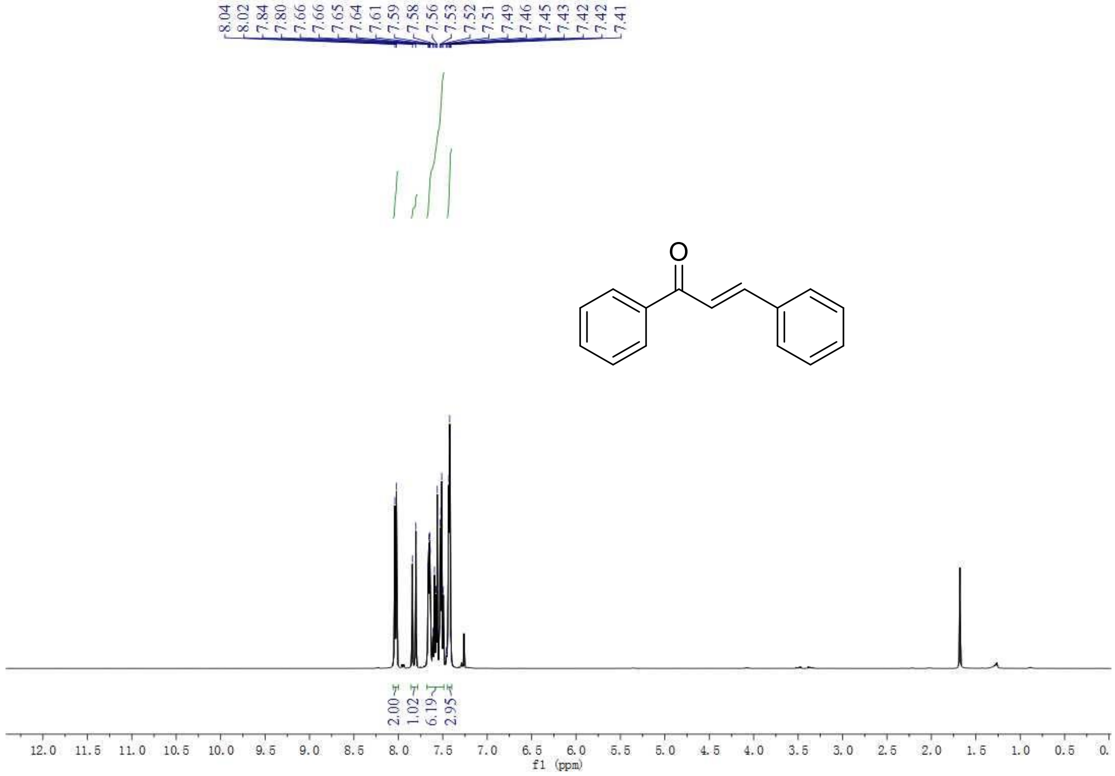

$^{13}C\{^1H\}$ NMR spectrum of (*E*)-chalcone (5d, 101 MHz, $CDCl_3$)

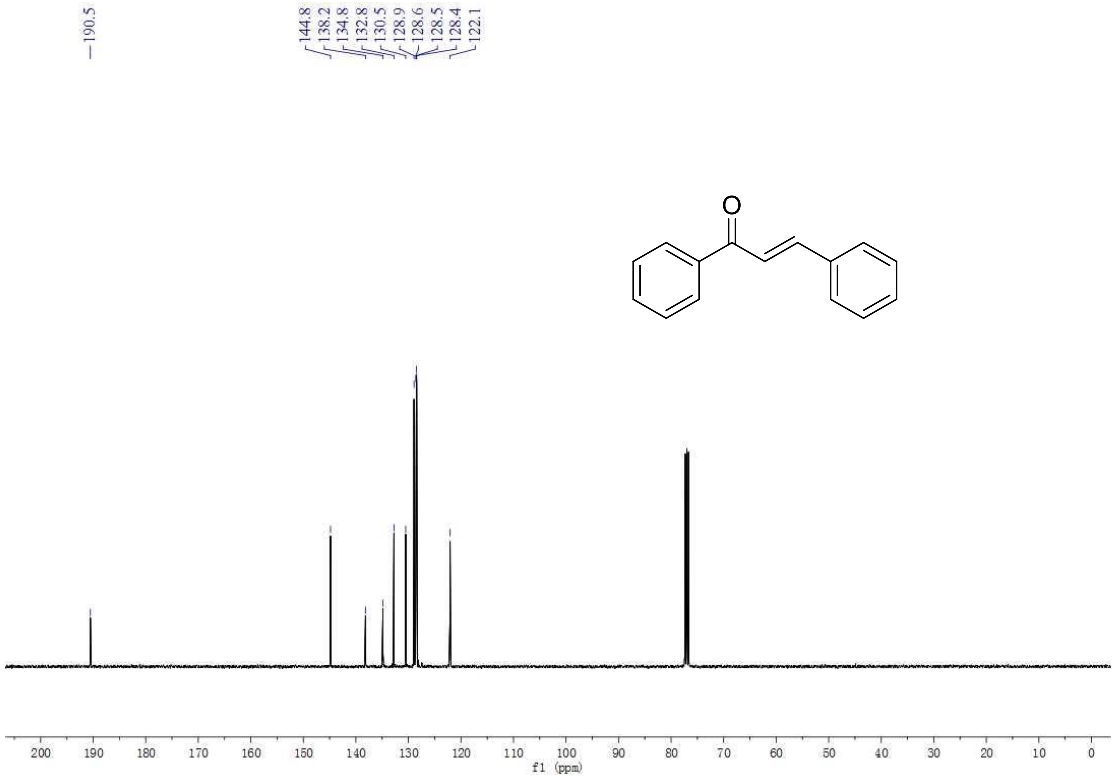

S5. References

1. Jiang, X.; Zhang, J.; Ma, S., Iron Catalysis for Room-Temperature Aerobic Oxidation of Alcohols to Carboxylic Acids. *J. Am. Chem. Soc.* **2016,** *138*, 8344-8347.
2. Dai, Z.; Qin, L.; Jiang, H.; Qi, L.; Hua, L.; Jing, Z.; Peng, T., Ni(II)–N′NN′ pincer complexes catalyzed dehydrogenation of primary alcohols to carboxylic acids and H2 accompanied by alcohol etherification. *Catal. Sci. Technol.* **2017,** *7*, 2506-2511.
3. Zhao, M.; Zhang, X.-W.; Wu, C.-D., Structural transformation of porous polyoxometalate frameworks and highly efficient biomimetic aerobic oxidation of aliphatic alcohols. *ACS Catal.* **2017,** *7*, 6573-6580.
4. Rafiee, M.; Konz, Z. M.; Graaf, M. D.; Koolman, H. F.; Stahl, S. S., Electrochemical oxidation of alcohols and aldehydes to carboxylic acids catalyzed by 4-acetamido-TEMPO: An alternative to “Anelli” and “Pinnick” oxidations. *ACS Catal.* **2018,** *8*, 6738-6744.
5. Xiao, C.; Zhang, L.; Hao, H.; Wang, W., High selective oxidation of benzyl alcohol to benzylaldehyde and benzoic acid with surface oxygen vacancies on W18O49/holey ultrathin g-C3N4 nanosheets. *ACS Sustain. Chem. Eng.* **2019,** *7*, 7268-7276.
6. Kim, S.; Lee, H. E.; Suh, J. M.; Mi, H. L.; Min, K., Sequential Connection of Mutually Exclusive Catalytic Reactions by a Method Controlling the Presence of an MOF Catalyst: One-Pot Oxidation of Alcohols to Carboxylic Acids. *Inorg. Chem.* **2020,** *59*, 17573-17582.
7. Ou, J.; Tan, H.; He, S.; Wang, W.; Hu, B.; Yu, G.; Liu, K., 1, 2-Dibutoxyethane-Promoted Oxidative Cleavage of Olefins into Carboxylic Acids Using O2 Under Clean Conditions. *J. Org. Chem.* **2021**, 14974−14982.
8. Shi, H.; Li, J.; Wang, T.; Rudolph, M.; Hashmi, A. S. K., Catalyst-and additive-free sunlight-induced autoxidation of aldehydes to carboxylic acids. *Green Chem.* **2022,** *24*, 5835-5841.
